# Gene regulatory innovations from transposable elements in primate cerebellum development

**DOI:** 10.1038/s41467-026-75700-7

**Published:** 2026-07-30

**Authors:** Tetsuya Yamada, Mari Sepp, Ioannis Sarropoulos, Henrik Kaessmann

**Affiliations:** 1 Center for Molecular Biology of Heidelberg University (ZMBH), DKFZ-ZMBH Alliance, Heidelberg, Germany; 2 Present Address: Centre of Genomics, Evolution and Medicine (cGEM), Institute of Genomics, University of Tartu, Tartu, Estonia; 3 Present Address: Wellcome Sanger Institute, Cambridge, UK; 4 Present Address: Cambridge Stem Cell Institute and Department of Medicine, University of Cambridge, Cambridge, UK

**Keywords:** Gene regulation, Evolutionary genetics, Transcriptomics, Machine learning, Genetics of the nervous system

## Abstract

Transposable elements are hypothesized to have driven gene regulatory innovation, yet their contributions to primate brain development at the cell type level remain underexplored. Here, we use single-cell multiomics data from human, macaque, marmoset, and mouse cerebella to show that transposable element contributions to different cell types are shaped by varying degrees of constraints across cell types, as well as the preferential co-option of certain transposable elements in specific cell states. Using a sequence-based deep-learning model that predicts cell-type-specific chromatin accessibility, we systematically assess the co-option potential of transposable elements into cerebellar gene regulatory networks, identifying twelve transposable element subfamilies with complex regulatory sequences in their ancestral states that facilitate their co-option as cell-type-specific cis-regulatory elements. Preservation of these ancestral regulatory sequences, as well as the active chromatin environment surrounding the insertion site, is the major determinant of the accessibility of extant copies. Lineage-specific accessible copies contribute to human-specific gene expression. Broadly, we demonstrate how transposable elements can be flexibly co-opted into cell-type-specific gene regulatory networks, and introduce a generalizable analytical framework for dissecting their contribution to mammalian regulatory evolution.

## Abstract (web summary)

The roles of transposable elements in primate brain development remain underexplored. Here, the authors identify transposable elements with ancestral regulatory potential that have been co-opted as cis-regulatory elements in primates.

## Introduction

The genetic basis of brain evolution, particularly in the primate lineage, remains to be fully elucidated. The cerebellum has undergone significant expansion in neuron numbers alongside the neocortex during primate evolution, with cerebellar granule cells—which develop from the upper rhombic lip in the hindbrain and constitute ~80% of all neurons in the human brain—playing a central role in this expansion^1,2^. Gene regulatory changes affecting gene expression are considered central to such evolutionary innovations^3^, as they can modify specific cellular processes without disrupting essential functions^4^. These regulatory changes occur predominantly through modifications to cis-regulatory elements (CREs)^5^, such as promoters and enhancers, which are bound by transcription factors (TFs) and regulate genes in a cell-type-specific manner^6^. Many evolutionary changes arise from single-nucleotide substitutions and small insertions or deletions (indels).

Transposable elements (TEs) offer another mechanism for regulatory innovation^7–9^. These DNA sequences, capable of self-replication and genomic translocation, have long been implicated in transcriptional regulation by rewiring gene regulatory networks through the expansion of copies harboring TF binding sites. This model of TE-driven regulatory evolution was already postulated in the 1950s by Barbara McClintock, who first identified TEs in maize and referred to them as ‘controlling elements’^10^, a concept that was later expanded as the ‘gene-battery’ model by Britten and Davidson^11–13^. Indeed, recent genome-wide studies on CREs across different tissues have revealed that TEs overlap approximately one-quarter of CREs^14–16^. Furthermore, some TEs are enriched for regulatory sequences, likely due to pre-existing TF binding sites in their ancestral sequences^14,17–20^. These lines of evidence suggest an important role of TEs in gene regulatory innovation.

However, the extent and mechanisms of TE contributions to regulatory innovations remain debated. First, despite comprising 45–70% of the human genome^21,22^, TEs appear to be underrepresented in CREs compared to their overall genomic abundance^23^. Second, the observed association between TEs and regulatory regions may partly reflect their inherent insertion site preferences, given that TEs from certain families tend to insert into open chromatin regions^24–26^. Third, TE insertions that affect gene expression often face rapid elimination through purifying selection, suggesting that their large-scale genomic alterations may be too disruptive to be readily co-opted for regulatory innovation^27^. Finally, most previous studies, with some recent exceptions^28,29^, relied on bulk tissue or isolated cell populations^14,16–20,30–32^ or lacked insights into the mechanisms of cell-type-specific TE co-option^33–36^, limiting our understanding of TE contributions to gene regulation, which fundamentally operates at the level of individual cell types within tissues. This limitation is particularly relevant for tissues with high cellular complexity, such as the developing brain.

Our previous work characterized CRE and gene expression innovations across different cell types in mammalian cerebellar development^37–39^. We developed a deep-learning model, DeepCeREvo, which learned the sequence grammar of cerebellar cell-type-specific CREs and applied it across the genomes of 240 mammalian species to understand the genetic basis of CRE and gene expression evolution in the lineage leading to humans^39^. This analysis identified hundreds of CRE innovations in each cell type, which occurred at different times points during evolution and include human-specific events. It also characterized single nucleotide substitutions and small indels potentially underlying these innovations. However, the contributions of TEs to these CRE innovations and the overall regulatory landscape in cerebellar cell types remains unexplored.

In this study, we combined single-cell multi-omics datasets of developing cerebellar cell types from human, macaque, marmoset, and mouse with our sequence-based deep-learning model to systematically investigate how TEs shape gene regulatory evolution in cerebellar development.

## Results

### Factors shaping TE contributions to cerebellar cis-regulatory elements

**Fig. 1:**
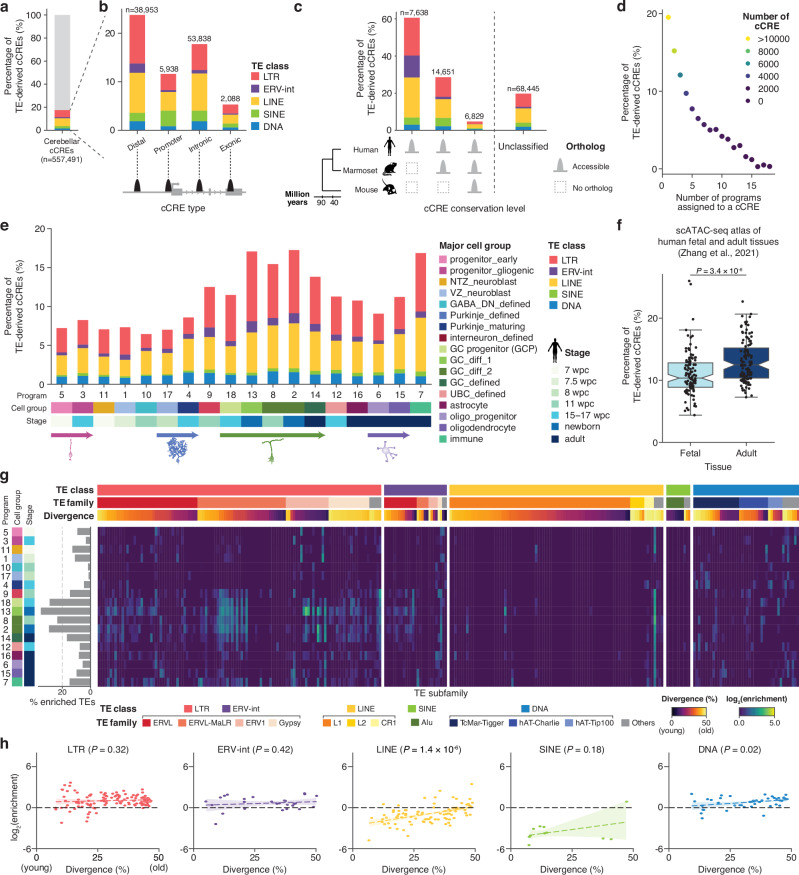
Contributions of transposable elements to cCREs in human cerebellar development. Percentage of TE-derived cCREs in human cerebellar development (**a**) across genomic contexts (**b**) or across accessibility conservation categories between human, marmoset, and mouse (**c**). **d** Relationship between cCRE accessibility specificity across programs and percentage of TE-derived cCREs. **e** Percentage of program-specific cCREs that originate from TEs across human cerebellar cell types and developmental stages. For each program, the cell group and developmental stage of highest activity are indicated, along with differentiation trajectories within each cell lineage. **f** Percentage of TE-derived cCREs in cell types from fetal (*n* = 111) or adult (*n* = 111) tissues across the human body, with each point representing a cell type. Box plots show the median (centre line), interquartile range (IQR; box bounds), and 1.5× IQR from the box bounds (whiskers). Comparisons were performed using two-sided Mann–Whitney *U* tests. Data from Zhang et al., 2021^48^ reanalyzed. **g** Subfamily-specific TE enrichment within program-specific cCREs. (Left) bar plot indicates the proportion of each enriched TE subfamily contributing to TE-derived cCREs. **h** Correlation between evolutionary age (median sequence divergence from the consensus) and TE enrichment in program-specific cCREs (maximum log_2_ enrichment across programs) for each TE subfamily, stratified by TE class. Linear regression equations and *P*-values are shown with 95% confidence intervals. *P*-values were estimated using two-sided *t*-tests from ordinary least squares linear regression. DN, deep nuclei neuron; ERV-int, internal sequences of endogenous retrovirus; GC, granule cell; LTR, long terminal repeat of endogenous retrovirus; NTZ, nuclear transitory zone; UBC, unipolar brush cell; VZ, ventricular zone; wpc, weeks post conception. Source data are provided as a Source Data file.

To assess the contributions of transposable elements (TEs) to the cis-regulatory landscape in mammalian cerebellum development, we intersected 557,491 candidate cis-regulatory elements (cCREs) accessible in the human cerebellum^39^ with annotated TEs^40^. Overall, 17.6% of human cCREs overlap with TEs (>250 bp overlap, representing >50% of cCRE length) (Fig. 1a). To determine whether this represents TE depletion compared to genomic abundance of TEs (~45%)^21^, we compared cCREs to length-matched random genomic regions using logistic regression, controlling for GC content, mappability, and distance to the transcription start site of the nearest gene. After accounting for these genomic features, cCREs showed significant TE depletion (odds ratio [OR] = 0.38, *P* < 10^−15^), representing a 62% reduction in the odds of TE overlap relative to the genomic background (Supplementary Data 1). Within cCREs, TE overlap varied significantly by regulatory element type. Distal cCREs exhibited the highest TE overlap (23.8%) (Fig. 1b). Relative to distal elements, exonic cCREs showed the strongest TE depletion (OR = 0.27, *P* < 10^−15^), followed by promoters (OR = 0.67, *P* < 10^−15^) and intronic elements (OR = 0.78, *P* < 10^−15^) (Supplementary Data 1). These patterns reflect the differential functional constraints of different regulatory element classes^41^.

To investigate the relationship between TE contributions to regulatory elements and evolutionary constraint, we used cerebellar cCREs from marmoset (n = 505,724) and mouse (n = 498,119)^39^ to classify human cCREs as human-specific, primate-specific, mammalian-conserved, or unclassified. Using logistic regression controlling for peak type and GC content, lineage-specific cCREs showed strong TE enrichment relative to unclassified cCREs: human-specific cCREs had 62% TE overlap (OR = 6.25, *P* < 10^−15^) and primate-specific cCREs had 29% TE overlap (OR = 1.82, *P* < 10^−15^) (Fig. 1c; Supplementary Data 1). Conversely, mammalian-conserved cCREs showed only 5% TE overlap (OR = 0.24, *P* < 10^−15^), though the contributions of older TEs to conserved cCREs may be underestimated due to difficulty of annotating highly degenerated TEs^42^. This pattern of TE enrichment in lineage-specific elements generalizes to marmoset and mouse cCREs. Specifically, 63% of marmoset-specific cCREs (OR = 5.86, *P* < 10^−15^) and 34% of mouse-specific cCREs (OR = 5.89, *P* < 10^−15^) overlap with TEs, whereas fewer than 5% of cCREs with conserved accessibility across species overlap with TEs in both marmoset and mouse (Supplementary Fig. 1a,b; Supplementary Data 1). The overall lower contribution of TEs in mouse cCREs likely reflects a lower genomic TE background in the mouse genome, faster TE sequence decay due to higher substitution rate in this lineage, and longer divergence time since the split from the common ancestor of the lineage encompassing primates and glires. These findings confirm that TE-derived cCREs are enriched in more recently emerged, evolutionarily less constrained regulatory elements across mammals^43–46^.

To characterize cell-state-specific patterns of TE integration into cCREs, we analyzed cCREs associated with 18 programs capturing cell-state- and time-specific chromatin accessibility patterns based on dimensionality reduction by non-negative matrix factorization^39^ (Table 1; Methods). We previously showed that these programs capture major regulatory activities characteristic of particular cell populations at defined developmental timepoints^39^, enabling systematic analysis of cell-state-specific TE contributions throughout cerebellar development. Using logistic regression to model TE overlap while controlling for peak type, conservation, and GC content, we found that TE content decreases progressively as cCREs are shared across more cell types. Elements shared across 2 programs showed 15% lower odds of TE overlap than cell-type-specific elements (OR = 0.85, *P* < 10^−15^), with the reduction increasing to 70% for elements shared across more than 9 programs (OR = 0.30, *P* < 10^−15^) (Fig. 1d; Supplementary Data 1). This trend, consistent with previous multi-tissue epigenomic studies^15,16,23,47^, may reflect the possibility that regulatory elements active in many cell types face stronger constraints and require more complex regulatory sequences to coordinate function across diverse cellular contexts, making them more challenging to evolve.

**Table 1:** Programs of chromatin accessibility in the developing cerebellum

Program	Major cell group	Contributing cell groups, unabbreviated (Developmental stage)
1	VZ_neuroblast	Ventricular zone neuroblast (CS18–19; CS20; CS22–23)
2	GC_diff_2	Differentiating granule cell 2 (newborn; infant); Defined granule cell (infant)
3	progenitor_gliogenic	Early progenitor (11 wpc); Bipotent progenitor (11 wpc; 15–17 wpc); Gliogenic progenitor (11 wpc; 15–17 wpc)
4	Purkinje_maturing	Defined Purkinje cell (11 wpc); Maturing Purkinje cell (15–17 wpc)
5	progenitor_early	Early progenitor (CS18–19; CS20; CS22–23)
6	oligo_progenitor	Oligodendrocyte progenitor cell (15–17 wpc; adult)
7	immune	Immune cell/microglia (adult)
8	GC_diff_2	Differentiating granule cell 1 (15–17 wpc); Differentiating granule cell 2 (11 wpc; 15–17 wpc)
9	interneuron_defined	Differentiating interneuron (11 wpc; 15–17 wpc); Defined interneuron (15–17 wpc)
10	GABA_DN_defined	Defined GABAergic deep nuclei neuron (CS20; CS22–23; 11 wpc)
11	NTZ_neuroblast	Differentiating isthimic neuron (CS18–19); Nuclear transitory zone neuroblast (CS18–19; CS20); Defined glutamatergic deep nuclei neuron (CS22–23)
12	UBC_defined	Differentiating unipolar brush cell (15–17 wpc); Defined unipolar brush cell (15–17 wpc)
13	GC_diff_1	Granule cell progenitor (newborn); Differentiating granule cell (newborn; infant)
14	GC_defined	Defined granule cell (infant; adult)
15	oligodendrocyte	Oligodendrocyte (adult)
16	astrocyte	Astrocyte (adult)
17	Purkinje_defined	Differentiating Purkinje cell (CS20; CS22–23); Defined Purkinje cell (CS22–23)
18	GCP	Granule cell progenitor (11 wpc; 15–17 wpc)

Annotation of 18 chromatin accessibility programs across cell types and developmental stages in the developing cerebellum, as identified by non-negative matrix factorization in Sarropoulos et al., 2026^39^.

CS, Carnegie stage; wpc, weeks post conception.

The contributions of TEs to cell-type-specific cCREs vary markedly across developmental stages (Fig. 1e). After controlling for peak type, GC content, and conservation category, cCREs accessible during embryonic development (~8 weeks post conception) showed 58% lower odds of TE overlap than adult-accessible elements (OR = 0.42, *P* < 10^−15^), and fetal-accessible cCREs (9–40 weeks post conception) showed 41% lower odds of TE overlap (OR = 0.59, *P* < 10^−15^) (Supplementary Data 1). To determine whether this pattern applies more broadly across cell types and organs, we re-analyzed data from a previously published single-cell chromatin accessibility dataset^48^ using logistic regression controlling for peak type, GC content, and sequence conservation. This analysis confirmed that fetal-accessible cCREs exhibit 18% lower odds of TE overlap than adult-accessible elements (OR = 0.82, *P* < 10^−15^) (Fig. 1f; Supplementary Data 1). Within fetal tissues, we observed tissue-specific variation, with relative underrepresentation of TE-derived cCREs in neural cell types and overrepresentation in placental cell types, consistent with the important role of TEs in placental function^49,50^ (Supplementary Fig. 2). Together, our results suggest that the differential abundance of TEs across cCREs reflects varying degrees of constraints across different cell types, with greater TE co-option occurring in more specialized, later-developing cell types in which regulatory innovation is facilitated by reduced selective constraints^37,51^.

TEs are classified hierarchically into classes, families, and subfamilies based on replication mechanisms and sequence similarities^40,52^ (Methods). The varied enrichment of TEs in cCREs across cell types may also be explained by preferential co-option of specific TE subfamilies into cell-type-specific regulatory elements^53,54^. To investigate general trends of subfamily-specific co-option, we quantified the enrichment of each TE subfamily in cCREs relative to its genomic abundance across each program (Fig. 1g; Supplementary Data 2). The proportion of TE-derived cCREs originating from overrepresented TE subfamilies (log_2_ enrichment > 1) varied substantially among cell types, ranging from 1.3% in Purkinje cells (program 17) to 35% in differentiating granule cells (program 13) (Fig. 1g). The proportion of putative co-option among TE-derived cCREs varied across cell lineages: consistently high in granule cell lineage (17–36%), followed by microglia (program 7) and interneurons (program 9) (~15%), and neural progenitors and neuroblasts (programs 1, 5, and 11) (9–11%). These lineage-specific patterns are reflected in the correlated enrichment of TE subfamilies across related programs: pairwise correlation analysis of TE enrichment scores revealed clustering within the granule cell lineage and among neural progenitors and neuroblasts (Supplementary Fig. 3), suggesting that individual TE subfamilies are frequently co-opted across multiple programs within related cell groups.

Subfamily-specific co-option also varies by TE class and subfamily age. TE subfamily ages were inferred from the median divergence of individual genomic copies from their consensus sequence, with higher divergence reflecting older insertions^55^ (Methods). Older TE subfamilies in LINE, SINE, and DNA classes show higher cCRE overlap than younger ones (Fig. 1h), likely reflecting more time for inserted copies to acquire regulatory function and for non-functional copies to degenerate. In contrast, younger LTR and ERV-int subfamilies are as enriched as older ones (Fig. 1h), suggesting these TE classes are more readily co-opted as regulatory elements, in line with previous reports^14,44,46^. Collectively, our results suggest that TE contributions to cCREs in different cell types are shaped by both constraints acting at multiple levels and preferential co-option of specific TE subfamilies in each cell type. This cell-type-specific co-option raises the question of what sequence features enable such preferential recruitment.

### Complex cis-regulatory sequences in ancestral TE sequences facilitate co-option of TEs

**Fig. 2:**
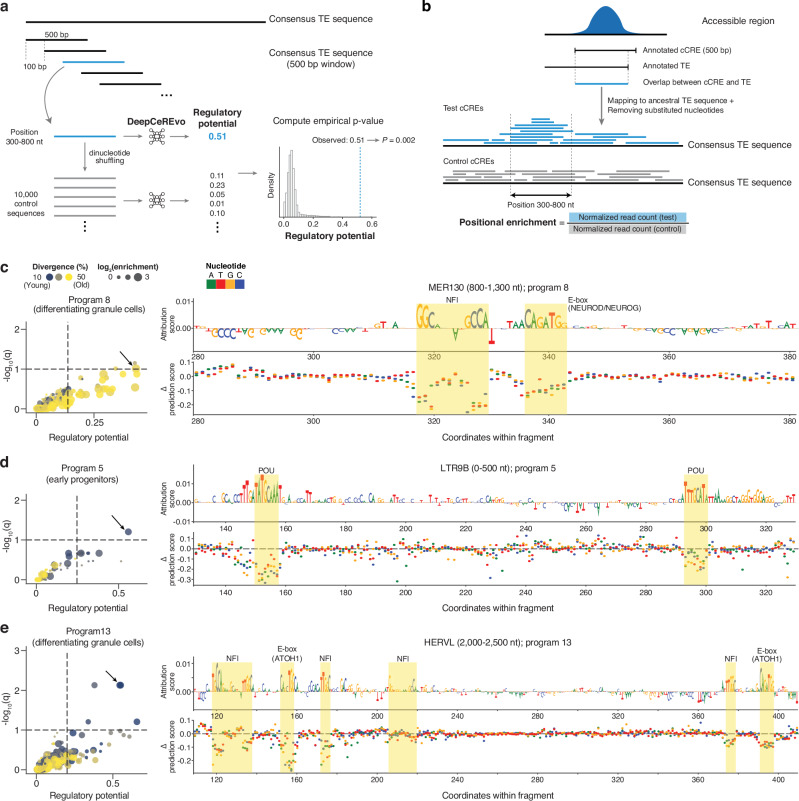
In silico screening for transposable elements with high regulatory potential. **a**, **b** Schematic overview of the workflows for evaluating the regulatory potential of consensus TE fragments using DeepCeREvo (**a**) and for assessing enrichment of cell-type-specific cCREs within specific regions of TE fragments compared to background cCRE sets (**b**). The workflows were applied separately for each program/cell state. **c**–**e** Identification of TE subfamily fragments with high regulatory potential and high contribution to cell-type-specific cCREs. Left: Screening of TE fragments based on regulatory potential scores and associated *P*-values, alongside enrichment in cell-type-specific cCREs for programs 8, 5, and 13, respectively. The threshold for regulatory potential was set at the 95th percentile. The *P*-value threshold was determined using the Benjamini–Hochberg procedure to control the false discovery rate (FDR) at 10%, adjusted for the effective number of tests. Right: DeepExplainer attribution and in silico mutagenesis profiles for top candidates (indicated by arrows in left panels). Source data are provided as a Source Data file.

The preferential co-option of certain TE subfamilies into cell-type-specific cCREs can be driven by pre-existing sequence features capable of functioning as regulatory elements, such as TF binding sites, as demonstrated in several TE subfamilies in specific tissues using reporter assays^17,19,20^. However, systematically assessing the cis-regulatory potential—the propensity to be co-opted as CREs based on pre-existing sequence features in their ancestral form—of diverse TE subfamilies across cell types requires more scalable approaches. To address this, we leveraged recent advances in sequence-to-function models predicting cCRE accessibility from DNA sequence^56–59^. Specifically, we applied DeepCeREvo, a deep-learning model trained on human and mouse cCRE sequences that predicts program-specific cCREs based solely on 500 bp DNA sequences^39^ (Fig. 2a). For each TE subfamily, we generated 500 bp sliding windows with 100 bp steps across the consensus sequence and analyzed 123 subfamilies that showed at least 10 overlaps with highly variable cCREs in at least one cell type, prioritizing those with high contributions to cell-type-specific chromatin accessibility. We computed cell-type-specific regulatory potential for each fragment using DeepCeREvo and compared it against dinucleotide-shuffled control sequences to determine statistical significance (Fig. 2a; Methods). Additionally, to quantify preferential co-option of these TE fragments, we compared their overlap enrichment with program-specific cCREs versus background cCREs from the whole human body at both fetal and adult stages^48^ (Fig. 2b).

Our screening analysis identified twelve TE subfamilies with high cis-regulatory potential for specific cell types in cerebellum development, with enriched overlap between extant copies and cCREs accessible in the corresponding cell types (Supplementary Fig. 4; Supplementary Data 3). These include MER130 in differentiating granule cells, previously identified as a DNA transposon co-opted into developmental enhancers in the mouse neocortex^28,32^ (Fig. 2c). Attribution analysis of DeepCeREvo revealed that the reconstructed ancestral sequence of this TE fragment contains an E-box (CAGATGG; NEUROD/NEUROG) and an NFI binding motif (GCCA) instances, both known to be important for brain development, including the differentiation of cerebellar granule cells^28,32,37,60,61^ (Fig. 2c). The other eleven TE subfamilies also contain TF binding motif instances corresponding to their cell type enrichment. These include LTR9B in early progenitors and MER41B, MER49, and MER72 in ventricular zone neuroblasts with multiple POU motif instances (ATAT(T/G)CA) (Fig. 2d; Supplementary Fig. 5a–c). The POU TFs are known to be active during early embryonic development and their binding motif instances are enriched in LTR sequences, facilitating transcription of proviral sequences in germ lines for intergenerational inheritance^54,62,63^. We also identified HERVL, ERVL-B4, LTR1A2, MER52D, and MamRTE1 in the granule cell lineage with E-box (CAG(C/A)TG; ATOH1) and NFI binding motif instances (Fig. 2e; Supplementary Fig. 5d–g). ATOH1 regulates genes essential for the development of cerebellar granule cells^64–66^. Finally, MamTip2b in mature Purkinje cells contains homeodomain motif (TAATTNNNAT; LHX1/5) instances and MLT1M in microglia contains PU.1 binding motif (GGAA) instances (Supplementary Fig. 5h,i)—TFs known to specify their respective cell lineages^67,68^. Collectively, using a deep-learning-based approach, we identified TE subfamilies with complex cis-regulatory sequences in their ancestral states that facilitate their preferential co-option as CREs in specific cell types.

### Lineage-specific co-option of TE subfamilies as regulatory elements

**Fig. 3:**
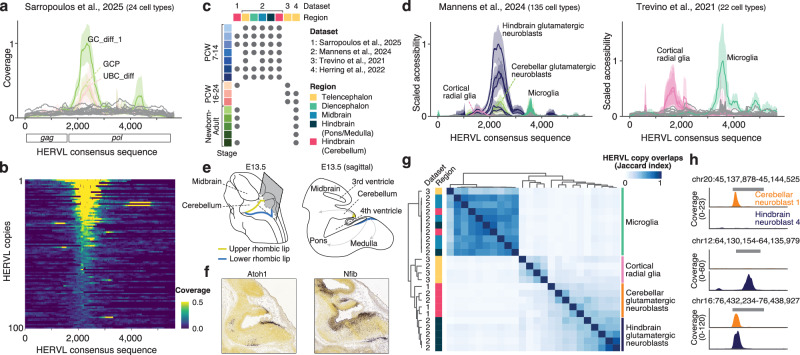
HERVL co-option in cCREs specific to rhombic lip–derived neuroblasts. **a** Mean chromatin accessibility profiles of the 50 most accessible HERVL copies across 24 cell groups in human cerebellar development, aligned to the consensus sequence, with the 95% confidence intervals based on bootstrapping analysis for the highlighted cell types. Regions homologous to gag (group antigen) and pol (polymerase) genes of the HERVL provirus are indicated below the coordinates. **b** Chromatin accessibility patterns of the 100 most accessible HERVL copies in the 2000–2500 nt position in differentiating granule cells (GC_diff_1), aligned to the consensus sequence. **c** Overview of chromatin accessibility datasets spanning multiple brain regions across developmental stages. **d** Mean accessibility profiles of the 50 most accessible HERVL copies across diverse cell types from different brain regions at various developmental stages (data from Mannens et al., 2024^61^ and Trevino et al., 2021^73^), aligned to the HERVL consensus sequence, with the 95% confidence intervals based on bootstrapping analysis for the highlighted cell lineages. **e** Schematic view of a mouse embryo highlighting the cerebellum at E13.5 (left) and sagittal section of the hindbrain along the plane indicated on the left (right). Arrows indicate the migration paths of rhombic lip–derived neuroblasts. **f** In situ hybridization images from the Allen Developing Mouse Brain Atlas (https://developingmouse.brain-map.org) showing expression of Atoh1 (left) and Nfib (right) in E13.5 mouse hindbrain. **g** Matrix of pairwise Jaccard similarity indices for the 50 most accessible HERVL copies across cell types highlighted in (**a** and **d**).** h** Examples of HERVL-overlapping cCREs specific to cerebellar neuroblasts 1 (top), hindbrain glutamatergic neuroblasts 4 (middle), or shared between these two cell types (bottom). Annotated HERVL copies are shown as gray bars. E, embryonic day.

The vast diversity of neural cell types is generated from a small pool of progenitors through spatiotemporal patterning cues and their downstream transcriptional programs^69,70^. Cell types originating from the same anatomical region or developmental window therefore share portions of their transcriptional programs, driven by common TFs^60,71,72^. Consistently, the screened TE subfamilies showed specific accessibility in their respective cell lineages. For instance, by projecting the accessibility profiles of all 2006 annotated HERVL copies in each cell type onto the corresponding consensus sequences, we found that HERVL copies are predominantly accessible in differentiating granule cells (GC_diff_1) and developmentally related cell groups, including granule cell progenitors (GCP) and differentiating unipolar brush cells (UBC_diff), with accessibility concentrated at the 2000–2500 nt position of the HERVL consensus sequence (Fig. 3a,b).

To investigate the specificity of TE co-option beyond the cerebellum, we analyzed chromatin accessibility across cell types in different brain regions during development using multiple datasets, such as a comprehensive atlas of the whole brain during the first trimester of human development^61^ (Fig. 3c). The 2000–2500 nt position of HERVL is exclusively accessible in glutamatergic neuroblasts in the hindbrain (pons, cerebellum, and medulla)—a pattern that holds even when including cerebral cortex datasets covering the second and third trimesters and postnatal development^73,74^ (Fig. 3d). All these glutamatergic lineages, including cerebellar granule cells, are derived from the upper and lower rhombic lips, based on the expression of orthologous marker genes (e.g., *Atoh1*, *Nfib*, *Barhl1*, and *Btbd11*) in mice (Fig. 3e,f; Supplementary Fig. 6a–d) and consistent with ATOH1 being a key regulator of these cell lineages^64,75–77^. The other TE subfamilies identified as co-opted in the granule cell lineage in our screening (ERVL-B4, LTR1A2, MER52D, MER130, and MamRTE1) also exhibit accessibility specific to rhombic lip–derived glutamatergic neuroblasts, though lineage specificity varies between subfamilies (Supplementary Fig. 6b). Evolutionarily older elements (MER130 and MamRTE1) showed broader enrichment across different cell lineages, particularly in glutamatergic neurons and late neural progenitors. The other TE subfamilies identified in our screening also show lineage-specific accessibility corresponding to their TF binding motifs instances. LTR9B, MER41B, MER49, and MER72, which contain POU binding motif instances and are enriched in cCREs specific to early cerebellar progenitors or ventricular zone neuroblasts (Fig. 2d; Supplementary Fig. 5a–c), are accessible in radial glia and glioblasts across brain regions, as well as in telencephalic glutamatergic neurons (Supplementary Fig. 7a,b). These patterns are consistent with the established roles of POU family TFs, particularly POU3 TFs, in neural progenitors and cortical neurons^78,79^. Among older TEs that predate the mammalian common ancestor, MamTip2b and MLT1M are specifically accessible in the Purkinje cell and microglial lineages, respectively, matching the lineage-specific expression of LHX1 and SPI1, respectively (Supplementary Fig. 7a,b). These results indicate that the identified TE subfamilies are co-opted in specific cell lineages where TFs that recognize their corresponding motifs are active.

The same TE subfamily can also be co-opted as regulatory elements in distinct cell types^29^. Microglia and cortical radial glia exhibit distinct accessibility patterns at different positions within the HERVL consensus sequence, a pattern consistent across datasets (Fig. 3d). Indeed, the 3300–3800 nt position of HERVL, where microglia show enriched accessibility, displays high regulatory potential in microglia and contains two PU.1 binding motif instances (Supplementary Fig. 8a,b). Similarly, LTR1A2 shows enriched accessibility not only in rhombic lip-–derived neuroblasts but also in oligodendrocyte precursor cells (Supplementary Fig. 6a,b), and its ancestral sequence exhibits high regulatory potential in the corresponding program and contains binding motif instances for SOX TFs, which are known to be important for oligodendrocyte differentiation^80^ (Supplementary Fig. 8c,d). These results suggest that, although we focused on twelve TE subfamilies based on conservative criteria, additional instances of TE co-option likely exist, including multiple co-option events within the same subfamily driven by distinct sequence features.

To investigate whether different cell types utilize the same or distinct copies, we computed pairwise Jaccard similarity indices based on the 50 most accessible HERVL copies across cell types. We identified three distinct clusters corresponding to microglia, cortical radial glia, and rhombic lip–derived neuroblasts (Fig. 3g), indicating that cell types co-opting different positions of the HERVL consensus sequence utilize largely non-overlapping sets of HERVL copies. We also observed variability within rhombic lip–derived neuroblasts (Fig. 3g,h), suggesting that these neuronal lineages utilize partially distinct sets of HERVL copies depending on their developmental state and lineage identity, which could contribute to cell state–specific gene expression patterns associated with differentiation timing and distinct migratory pathways from the rhombic lips^64,75–77^. Similar patterns were observed in LTR1A2, with distinct clusters identified for microglia, oligodendrocyte precursors, and rhombic lip–derived neuroblasts, and finer heterogeneity observed within the rhombic lip–derived neuroblasts (Supplementary Fig. 9a,b). Collectively, these results illustrate the flexibility of TE co-option: the same TE subfamily can be co-opted by multiple cell states in the same cell lineage sharing regulatory programs, or by distinct cell states through different motif instances within the ancestral sequence, with variation also observed at the level of individual genomic copies.

### Subfamily-specific sequence substitutions create high regulatory potential in hindbrain neuroblasts

**Fig. 4:**
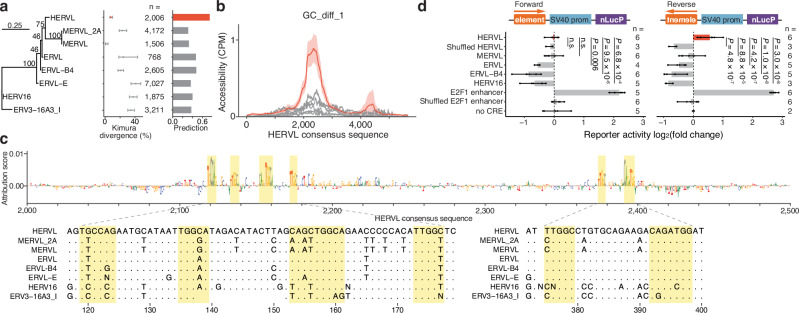
HERVL-specific sequence substitutions create unique enhancer activity. **a** Phylogenetic relationship of HERVL and subfamilies closely related to HERVL (left) alongside their median divergence (with interquartile range) of extant copies and DeepCeREvo prediction scores of the consensus sequences for differentiating granule cells (program 13) (right), based on regions orthologous to the 2000–2500 nt position in HERVL. Numbers in the internal nodes of the phylogenetic tree indicate bootstrap confidence values (%). **b** Mean accessibility profile of the 50 most accessible copies of internal regions from HERVL and other transposon subfamilies in the ERVL family, with the 95% confidence intervals based on bootstrapping analysis for HERVL. **c** Multiple sequence alignment of consensus sequences of internal regions of other transposon subfamilies in the ERVL family compared to HERVL, highlighting regions with high DeepExplainer attribution scores. **d** Luciferase reporter assays in mouse primary granule cells testing the enhancer activity of regions homologous to HERVL 2000–2500 nt position in other transposon subfamilies in the ERVL family. The E2F1 fragment is a conserved enhancer active in differentiating granule cells^39^. Shuffled fragments were obtained by shuffling sequences while keeping the dinucleotide frequency. Candidate fragments were placed in front of the SV40 promoter in forward (left) and reverse (right) orientation. Bars and error bars display the mean normalized and scaled reporter activity and its range; points denote independent experiments. *P*-values against the HERVL fragment were estimated using two-sided linear mixed models, corrected for multiple testing using the Benjamini–Hochberg method. Exact *P*-values are provided in Supplementary Data 4. Source data are provided as a Source Data file.

Long terminal repeats (LTRs) of endogenous retroviruses function as transcriptional regulatory elements for retroviral genes and harbor a high density of TF binding sites, making them a major source of TE-derived regulatory elements^45,46^. Consistent with this, the majority of TE subfamilies identified in our screening (7 of 12) are LTR elements. The internal protein-coding regions of endogenous retroviruses, by contrast, have received less attention as potential sources of co-opted regulatory elements, given their original protein-coding function, yet our screening identified two such cases: HERVL and ERVL-B4, whose internal sequences show highly specific accessibility in differentiating granule cells. HERVL is of particular interest given its evolutionary novelty, being shared only within the primate lineage, and its highly specific co-option in rhombic lip–derived neuroblasts (Figs. 3a–d; 4a). Moreover, the co-opted 2000–2500 nt position lies within the reverse transcriptase domain of the polymerase gene, one of the most conserved regions in otherwise fast-evolving endogenous retroviruses and frequently used to establish phylogenetic relationships among retroviruses^81,82^. These features prompted us to investigate the sequence evolution of TE subfamilies associated with HERVL to understand how this enhancer potential in HERVL emerged.

Comparative analysis of the consensus sequences of HERVL and seven closely related TE subfamilies revealed that only HERVL exhibits significantly higher chromatin accessibility in differentiating granule cells at the 2000–2500 nt position (Fig. 4b). Consistent with this, DeepCeREvo analysis for differentiating granule cells (program 13) showed that other TE subfamilies in the ERVL family are assigned 36–58% lower DeepCeREvo prediction scores compared to HERVL (0.31–0.48 vs. 0.74) at regions homologous to the HERVL 2000–2500 nt position (Fig. 4a). We identified multiple HERVL-specific substitutions in regions with high attribution scores that correspond to putative ATOH1 and NFI binding sites (Fig. 4c). These patterns were observed not only in the consensus sequences but also in individual genomic copies of the TE subfamilies associated with HERVL, confirming that they are not artifacts of ancestral sequence reconstruction (Supplementary Fig. 10). Our findings indicate that the enhancer potential of HERVL in rhombic lip–derived neuroblasts arises from sequence substitutions unique to this subfamily.

To assess whether the high regulatory potential specific to HERVL translates to enhancer activity, we performed luciferase reporter assays in primary cultures of mouse granule cells isolated from P7 mouse cerebella and cultured ex vivo for 3 days. We previously showed that this system resembles the trans-regulatory environment of human granule cells, observing similar TF expression and enhancer activity for 1:1 orthologous human and mouse CRE sequences^39^. We tested consensus sequences from four TE subfamilies closely related to HERVL and a dinucleotide-shuffled HERVL control, focusing on regions homologous to the HERVL 2000–2500 nt position. Among the tested sequences, HERVL exhibited significantly higher luciferase activity than dinucleotide-shuffled HERVL and other closely related subfamilies when tested in the reverse orientation, but not in the forward orientation (Fig. 4d; Supplementary Data 4). This suggests directional effects on gene regulatory activity, consistent with earlier reports that enhancers are not fully orientation-independent^83^. These results suggest that subfamily-specific sequence substitutions in HERVL contribute to its enhancer activity in hindbrain neuroblasts, distinguishing it from closely related elements.

### Determinants of copy-level TE co-option

**Fig. 5:**
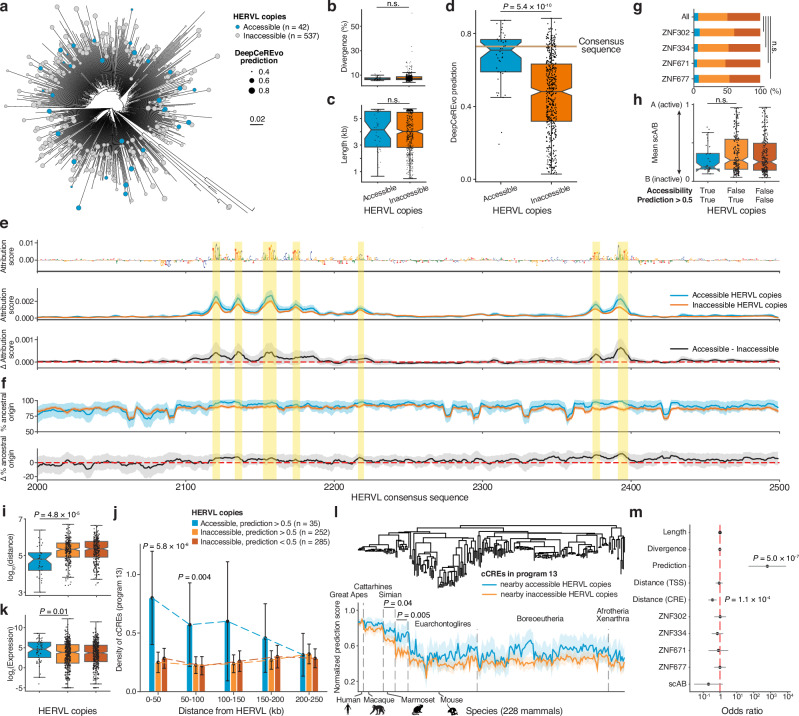
A subset of HERVL copies with high enhancer potential are accessible and involved in gene regulation in differentiating granule cells. **a** Phylogenetic relationship of HERVL copies in the human genome, annotated with chromatin accessibility status in differentiating granule cells (GC_diff_1) and corresponding DeepCeREvo prediction scores (program 13). Sequence divergence from the consensus (**b**), length (**c**), and DeepCeREvo prediction scores for differentiating granule cells (program 13) (**d**) of accessible (*n* = 42) and inaccessible (*n* = 537) HERVL copies. Comparisons were performed using two-sided Mann–Whitney *U* tests. **e** DeepExplainer attribution profile of the HERVL consensus sequence (top); mean DeepExplainer attribution profiles of accessible and inaccessible HERVL copies (middle) and the difference between them (bottom), with the 95% confidence interval shown. TF binding motif instances in the consensus sequence are highlighted. **f** Mean percentage of nucleotides identical to the consensus sequence in accessible versus inaccessible HERVL copies (top), and the difference between them (bottom), with the 95% confidence interval shown. **g** Percentage of HERVL copies within binding sites of the respective KZFPs. Enrichment was assessed using two-sided Fisher’s exact test. Compartment score (**h**), log_2_ distance to the closest non-TE-derived cCRE specific to differentiating granule cells (**i**), mean density of surrounding non-TE-derived cCREs with the 95% confidence intervals (**j**), and expression levels of nearby genes (**k**) for accessible HERVL copies with DeepCeREvo prediction score higher than 0.5 (*n* = 35), and inaccessible HERVL copies with DeepCeREvo prediction score higher (*n* = 252) or lower (*n* = 285) than 0.5. Comparisons were performed using two-sided Mann–Whitney *U* tests. **l** Mean DeepCeREvo prediction scores (program 13) for human cCREs proximal to accessible and inaccessible HERVL copies, and their orthologous genomic regions across 227 placental mammalian species with the 95% confidence intervals. Comparisons were performed using two-sided Mann–Whitney *U* tests. **m** Point estimates of odds ratios and 95% confidence intervals for each covariate in logistic regression models predicting accessibility of individual HERVL copies. *P*-values for individual covariates were estimated using two-sided Wald tests. No adjustments were made for multiple comparisons. Box plots show the median (centre line), interquartile range (IQR; box bounds), and 1.5× IQR from the box bounds (whiskers). Source data are provided as a Source Data file.

Although the screened TE subfamilies harbor regulatory potential in their ancestral sequences, individual genomic copies vary in whether they become co-opted as regulatory elements. For instance, despite having high regulatory potential in their ancestral state, only 2.1% of HERVL copies are accessible in differentiating granule cells, raising the question of what distinguishes accessible copies from inaccessible ones. Using HERVL as a case study, we first examined whether accessible copies derive from a distinct sublineage. Phylogenetic analysis revealed that accessible and inaccessible copies are distributed throughout the reconstructed evolutionary tree without forming distinct clusters (Fig. 5a; Supplementary Fig. 10), and we found no significant differences in sequence divergence or element length (Fig. 5b,c). In contrast, DeepCeREvo prediction scores were significantly higher for accessible copies (Fig. 5d; *P* < 10^−10^, Mann–Whitney *U* test). The median score of accessible copies (0.72) was comparable to that of the consensus sequence (0.74), suggesting that HERVL accessibility reflects preservation of pre-adaptive sequences from the ancestral element rather than convergent acquisition of regulatory motif instances through mutations after insertion. Consistently, sequences at positions corresponding to ancestral TF binding motif instances are recurrently used across copies and significantly more likely to be preserved in accessible copies (Fig. 5e,f; Supplementary Fig. 11; *P* < 10^−13^, linear regression), with no consistently acquired high-attribution positions beyond ancestral motifs. Together with the luciferase assay results (Fig. 4d), these findings indicate that HERVL harbored “ready-to-use” pre-adaptive regulatory sequences at the time of insertion.

However, 47% of inaccessible HERVL copies received DeepCeREvo prediction scores greater than 0.5, indicating that sequence features alone cannot explain all cases of inaccessibility. We hypothesized that epigenetic silencing mechanisms might suppress these high-potential elements. To test this, we examined two candidate mechanisms: KRAB domain-containing zinc finger proteins (KZFPs), which are known to target specific TE families and induce heterochromatin formation^84^, and higher-order chromatin organization reflected in megabase-scale A/B compartments, where B compartments are generally associated with transcriptional repression^85^. Analysis of KZFP binding sites using published datasets revealed no significant enrichment in inaccessible copies (Fig. 5g; Methods), and megabase-scale A/B compartments showed no differences between accessible and inaccessible copies (Fig. 5h; Methods). Although neither silencing mechanism explained inaccessibility, the surrounding regulatory landscape showed significant differences. Accessible HERVL copies are located significantly closer to non-TE-derived cell-type-specific cCREs (median 64 kb versus 221–234 kb; *P* < 10^−4^, Mann–Whitney *U* test) (Fig. 5i), with the strongest enrichment within 50 kb (*P* < 10^−5^, Mann–Whitney *U* test) (Fig. 5j). This proximity effect persists after controlling for genomic and sequence features including distance to TSS (*β* = −0.52, P < 10^−6^, linear regression). Consistently, genes nearby accessible copies show higher expression levels than those near inaccessible copies (Fig. 5k). To assess whether accessible copies activate neighboring chromatin upon insertion or reside in pre-existing permissive environments, we examined the evolutionary history of surrounding cCREs using DeepCeREvo prediction scores across 228 mammalian species^39^. cCREs near accessible HERVL copies have significantly older evolutionary origins than those near inaccessible copies (Fig. 5l), suggesting that accessible copies tend to reside in chromatin environments that were already active prior to HERVL insertion. To integrate these observations and quantify the relative contributions of all factors examined above, we performed multivariate logistic regression. This analysis confirmed that DeepCeREvo prediction score (OR = 1205, *P* < 10^−6^) and proximity to cell-type-specific cCREs (OR = 0.28 per log-unit increase in distance, *P* < 10^−4^) are the dominant predictors of HERVL accessibility, while other factors showed no significant independent effects (Fig. 5m; Supplementary Data 5). These results suggest that, in addition to local sequence features, proximity to active regulatory elements also matters for the co-option of TE subfamilies at the individual copy level^20^.

We next examined whether these patterns extend to other screened TE subfamilies. Multivariate logistic regression revealed that DeepCeREvo prediction scores are the strongest predictors for all subfamilies, that proximity to non-TE cCREs contributes significantly in 75% of subfamilies, and that KZFP binding shows significant effects in only 25% (Supplementary Fig. 12; Supplementary Data 5). The predictive power of DeepCeREvo scores reflects ancestral motif preservation: in young TE subfamilies, sequences at ancestral TF binding motif positions are recurrently used and significantly more preserved in accessible copies (Supplementary Fig. 13a–e; *P* < 0.05, linear regression), whereas in older subfamilies, these positions show significantly stronger purifying selection across 447 mammalian genomes^86–88^ (Supplementary Fig. 14a–d, *P* < 0.05, linear regression; Supplementary Data 6). Notably, not all subfamilies identified in our screening follow the HERVL pattern of “ready-to-use” pre-adaptive sequences. Comparison of DeepCeREvo prediction scores between accessible copies and the ancestral sequence revealed that extant LTR1A2 copies frequently possess significantly higher prediction scores than the ancestral sequence (Supplementary Figs. 15, 16a; *P* < 10^−10^, Mann–Whitney *U* test). Attribution analysis further showed that accessible copies frequently acquired additional TF binding motif instances (Supplementary Fig. 16b). Moreover, some of these additional motif instances were acquired at orthologous positions across independent copies that are distributed throughout the LTR1A2 phylogenetic tree, confirming that the motifs arose independently through convergent evolution (Supplementary Fig. 16c). These convergently acquired motif sequences in extant copies can be explained by transition mutations, which are known to occur at higher rates than transversions^89^, particularly at CpG sites via spontaneous deamination of methylated cytosine^90^ (Supplementary Fig. 16d). These findings suggest that the ancestral sequences of LTR1A2 provided proto-regulatory potential that was subsequently augmented through convergent acquisition of new motif instances, likely facilitated by mutation bias acting on proto-motif sequences in the ancestral TE^20^. Collectively, our results reveal that TE co-option is governed by two principal factors: preservation of ancestral TF binding motifs—with some subfamilies frequently acquiring additional substitutions that create new TF binding motif instances—and proximity to pre-existing active chromatin, with their relative contributions varying across subfamilies.

### Species-specific TE co-option drives regulatory divergence in primate cerebellar development

**Fig. 6:**
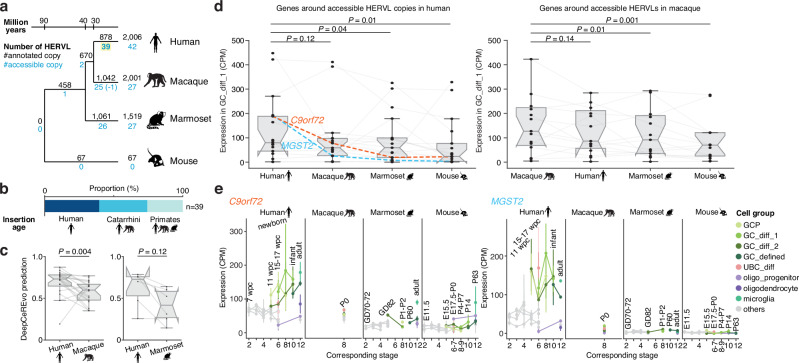
Species-specific accessible HERVL copies contributed to gene expression divergence during primate evolution. **a** Number of annotated and accessible HERVL copies in differentiating granule cells (GC_diff_1) across human, macaque, marmoset, and mouse. **b** Insertion age distribution of HERVL copies accessible in differentiating granule cells specifically in human. **c** DeepCeREvo prediction scores for differentiating granule cells (program 13) comparing human accessible HERVL copies with their corresponding orthologous sequences in macaque (*n* = 20) (left) and marmoset (*n* = 6) (right). Box plots show the median (centre line), interquartile range (IQR; box bounds), and 1.5× IQR from the box bounds (whiskers). Comparisons were performed using one-sided Wilcoxon rank-sum tests. **d** Expression levels of 1:1 orthologous genes located near HERVL copies accessible in differentiating granule cells (GC_diff_1) in human (*n* = 17) (left) and macaque (*n* = 15) (right). Comparisons were performed using one-sided Wilcoxon rank-sum tests. **e** Spatiotemporal expression profiles of *C9orf72* (left) and *MGST2* (right) across corresponding cell types and developmental stages in mammalian species. Points represent mean expression and bars indicate the range across biological replicates. CPM, counts per million. E, embryonic day; GD, gestational day; P, postnatal day; wpc, weeks post conception. Source data are provided as a Source Data file.

TEs with intrinsic regulatory potential provide a substrate for repeated, independent co-option events, raising the possibility that the same TE subfamily is deployed differently across species to drive lineage-specific gene expression. To test whether this occurs during primate brain development, we analyzed our previously generated single-cell transcriptome and chromatin accessibility datasets from macaque, marmoset, and mouse cerebellum, focusing on HERVL, LTR1A2, and MER52D—primate-specific elements enriched in differentiating granule cells, a cell state well-captured across all four species^39^. These TE subfamilies are largely primate-specific, present in comparable numbers across human, macaque, and marmoset but nearly absent in mouse (Fig. 6a; Supplementary Fig. 17a,b). In both macaque and marmoset, these elements show cell-type-specific accessibility in differentiating granule cells, matching the pattern observed in human (Supplementary Fig. 18). We therefore asked whether the same individual TE copies are co-opted across species, or whether different copies become accessible in each lineage. The presence of a TE copy in the genome does not guarantee its regulatory co-option: while 56–73% of human copies are genomically shared with macaque or marmoset, only 7–21% of accessible human copies are also accessible in these species (Fig. 6a; Supplementary Fig. 17a,b). This species-specific accessibility largely reflects differential co-option rather than differential insertion. Among copies specifically accessible in human, 24–41% represent human-specific insertions, while the remainder have orthologous but inaccessible copies in macaque or marmoset (Fig. 6b; Supplementary Fig. 17c,d). To understand what underlies these species-specific differences in accessibility, we compared DeepCeREvo prediction scores between accessible copies and their orthologous inaccessible copies. Human-specific accessible HERVL copies have significantly higher DeepCeREvo prediction scores than their orthologous inaccessible copies in macaque and marmoset (*P* < 0.05, Wilcoxon signed-rank test) and vice versa (Fig. 6c; Supplementary Fig. 19a,b). In addition to prediction scores, the presence of nearby cCREs specific to differentiating granule cells also contributes to HERVL accessibility in marmoset, with a similar trend observed in macaque (Supplementary Fig. 19c,d), suggesting that species-specific chromatin environments may also contribute to species-specific accessibility. LTR1A2 and MER52D showed similar patterns, with species-specific accessibility associated with both higher DeepCeREvo prediction scores and the presence of nearby cell-type-specific cCREs (Supplementary Fig. 17e–j). Together, these results indicate that accessible TE copies are predominantly species-specific, reflecting lineage-specific insertions, preservation of ancestral TF binding motifs, and local chromatin environment.

To determine whether species-specific TE co-option influences gene regulation, we examined expression patterns of genes near accessible copies in differentiating granule cells. Genes associated with human-specific accessible HERVL copies exhibit elevated expression in human compared to macaque and marmoset (Fig. 6d; Supplementary Data 7). These include *C9orf72*, whose dysfunction impairs neurodevelopment in human induced pluripotent stem cells and mouse models^91,92^, and *MGST2*, which produces a key signaling molecule in response to oxidative stress, to which cerebellar granule cells are particularly susceptible^93^. The increased expression is specific to or most prominent in differentiating granule cells (Fig. 6e), consistent with the cell-type-specific regulatory potential of HERVL. Macaque-specific accessible HERVL copies show the same pattern, with nearby genes exhibiting higher expression in macaque than in other species (Fig. 6d). Despite this elevated expression in species with accessible copies, these genes still show detectable expression in species where the orthologous TE copy is inaccessible, consistent with our earlier finding that accessible copies tend to reside in pre-existing active chromatin environments. This suggests that TE insertions augment rather than establish regulatory activity. Elevated expression around species-specific accessible copies was also observed for LTR1A2 and MER52D, and *C9orf72* is also located near a human-specific accessible LTR1A2 copy (Supplementary Fig. 20a,b). Collectively, these results indicate that TE subfamilies with intrinsic regulatory potential have been repeatedly and independently co-opted as species-specific enhancers during primate evolution, integrating into pre-existing active chromatin contexts to amplify divergent gene expression patterns in cerebellar granule cells.

## Discussion

In this study, we investigated the contributions of TEs to the chromatin accessibility landscape in cerebellum development and uncovered underlying evolutionary and genetic mechanisms using cross-species single-cell multi-omics atlases, along with a sequence-based deep-learning model predicting cell-type-specific chromatin accessibility.

Preferential co-option of TEs due to the presence of complex cis-regulatory sites in the ancestral forms of TEs has been widely documented, particularly in the LTR family during early embryogenesis^14,44,46^. LTR elements typically possess binding sites for TFs active during early development such as NANOG, SOX2, and OCT4 (POU5F1), which drive the expression of their proviral sequences in the germline lineage, facilitating amplification of these elements within genomes through vertical transmission^45,54,62,63,94,95^. While these TF binding sites can be co-opted in stem cells or progenitors where similar TFs are active, the preferential co-option of TEs in differentiating or differentiated cell types has remained less characterized. This knowledge gap exists primarily because detailed molecular profiles such as histone modifications or TF binding patterns across diverse cellular contexts have been limited, constraining most analyses to bulk tissues or isolated cell populations^14,16–20,30–32^.

Our study addresses this limitation by combining single-cell chromatin accessibility profiles with a sequence-based deep-learning model, DeepCeREvo, that predicts cell-type-specific chromatin accessibility to identify twelve TE subfamilies with high regulatory potential in specific cell types in developing cerebellum. Among these, HERVL and ERVL-B4 are internal sequences of endogenous retroviruses (ERV-int), suggesting that readily co-optable regulatory sequences can also arise in TE classes not canonically associated with regulatory activity. We also revealed that some of these preferentially co-opted TE subfamilies, as shown for HERVL and LTR1A2, harbor other sequence features that drive co-option in different cell types, as recently reported for the rodent-specific ORR1D2 and ORR1E subfamilies in immune cell types^29^. Furthermore, we used DeepCeREvo to show that preservation of ancestral sequences and proximity to pre-existing active chromatin contribute to co-option at the individual copy level, in line with observations of RSINE1 co-opted as a circadian enhancer in mouse^20^. Notably, this ancestral sequence preservation operates through distinct modes: HERVL harbors “ready-to-use” pre-adaptive sequences, whereas LTR1A2 possesses proto-regulatory potential that was subsequently augmented through convergent acquisition of new TF binding motif instances across independent copies. These lines of evidence further illustrate the multifaceted nature of TE co-option as regulatory elements, while also demonstrating the utility of sequence-based models in understanding this process at cell-type resolution across TE subfamilies.

In addition to these preferential co-option events of specific TE subfamilies, our results suggest that evolutionary constraints have a broad impact on shaping the overall distribution of TE-derived cCREs across cell types. This conclusion is supported by consistent patterns observed in our study and others showing that TE-derived cCREs are enriched in less-constrained genomic contexts: distal elements rather than promoters or exonic elements, species-specific rather than conserved regions, cell-type-specific rather than broadly accessible elements^23^, and elements active in later rather than earlier developmental stages^61^. These patterns are consistent with evolutionary processes—mutation, natural selection, and genetic drift—where inserted TE copies serve as raw material for regulatory evolution similarly to other non-TE-derived sequences, rather than providing a “shortcut” via pre-adaptive sequences in their ancestral state. This model is further supported by older TE subfamilies from LINE, SINE, and DNA classes showing higher cCRE enrichment than younger ones, while the absence of this age dependence in TE subfamilies from LTR and ERV-int classes points instead to preferential co-option via pre-existing regulatory sequences. Together, these observations highlight that understanding TE contributions to regulatory evolution requires considering both evolutionary constraint and preferential co-option driven by ancestral sequence features.

Our study has several limitations. First, our reliance on short-read sequencing makes it challenging to accurately profile chromatin accessibility in recently expanded long TEs with very low sequence divergence, such as L1HS, SINE-VNTR-Alu (SVA), and HERVK (HML-2)^96^. However, since these elements constitute only a fraction of annotated TEs, this limitation is unlikely to significantly affect our main conclusions. An additional limitation is the relatively small input window (500 bp) of our sequence-based model, DeepCeREvo. This input size is sufficient to capture local sequence features such as TF binding motif instances, but a longer input window would allow for incorporating longer stretches of ancestral sequence, making it possible to evaluate the regulatory potential of long TE classes (e.g., LINE, ERV-int) as a whole, as well as the surrounding genetic context of extant TE copies, which could shed light on the effect of surrounding regulatory elements more clearly. Applying a sequence-based model with a larger input window, such as Borzoi^97^ or AlphaGenome^98^ (524 kb and 1 Mb input windows, respectively), to TE studies represents a promising direction for future research.

Our work provides a framework to study TE contributions to cis-regulatory landscapes and to gene regulatory evolution across different cell types. This approach could extend beyond cerebellar development by integrating large-scale chromatin accessibility atlases with sequence-based deep-learning models^33,48,61,99^. Collectively, the framework and findings of this study may help inform future research into the contributions of TEs to the evolution of gene regulation and, ultimately, organismal phenotypes.

## Methods

### Ethics statement

All mouse procedures were performed in compliance with national and international ethical guidelines and regulations, and were approved by the local animal welfare authorities at Heidelberg University Interfaculty Biomedical Research Facility (T-08/24, T-37/24). The animals were housed under a 12 h/12 h dark/light cycle in a temperature (20–24 °C) and humidity (40–65%) controlled room with ad libitum access to food and water.

This study did not involve the collection of new data from humans, macaques, or marmosets. All human, macaque, and marmoset datasets used in this work were previously published^39^, and ethical approvals for the original data collection are described in the respective studies. Accordingly, no additional ethical approval was required for the present study.

### Single-cell multi-omics atlas of cerebellum development

The snRNA-seq and snATAC-seq datasets across cerebellum development in human, marmoset, and mouse used in this study are the same as our previous study^39^. For cross-species comparison of cell-type-specific chromatin accessibilities, cCREs specific to certain cell types in certain developmental stages in different species were grouped into programs based on non-negative matrix factorization (NMF), a dimensionality reduction approach that identifies recurring patterns of chromatin accessibility across cell types and developmental stages, as described in our previous study^39^. In short, standardized pseudobulk accessibility matrices of human and mouse cCREs for 45 sample groups [cCREs × samples] (combinations of cell types and developmental stages, which correspond in both human and mouse) were decomposed into two new matrices [cCREs × programs] and [programs × samples] via a predetermined number of programs. The number of programs was determined to be 18 based on the reconstruction error and the distance between cCREs from human and mouse in the same group. The order of the programs follows that of the original publication to maintain consistency between publications. We assigned human and mouse highly variable cCREs to each program based on the [cCREs × programs] matrix (cCRE loadings). For marmoset, the cCRE loading matrix ([cCREs × programs]) was obtained by using non-negative least squares on the [cCREs × samples] matrix in marmoset and the [programs × samples] matrix obtained in the human and mouse analysis. Similarly, all highly accessible peaks in human, marmoset, and mouse were assigned to programs based on the same transformation and the same cCRE loading threshold.

### Overlap between TEs and cCREs in mammalian cerebellum development

To identify the overlap between transposable elements and the cCREs accessible in cerebellum development, we intersected cCREs with annotated transposable elements in human, marmoset, and mouse genomes. We downloaded non-overlapping repeat annotations for human (hg38), macaque (rheMac8), marmoset (ASM275486v1), and mouse (mm10) from the Dfam database (v3.8)^40^. Repeat annotations for ASM275486v1 marmoset assembly were transferred to the calJac4 assembly using UCSC liftOver with a custom chain file generated by nf-LO^100^. Only repeat annotations whose classes belong to LTR, LINE, SINE, and DNA were retained. Since the LTR class contains both long terminal repeats and internal elements of endogenous retroviruses (ERVs), and these sequences show significant differences, this class was further classified into LTR and ERV-int subclasses. This classification was mostly based on the description in the Dfam database when available, which was the case in nearly 95% of the transposons in the LTR class. Otherwise, it was based on a length threshold of 2000 bp. We intersected these curated transposable element annotations with CRE annotations using bedtools (v2.30.0)^101^ and only retained overlaps longer than 250 bp (i.e., half of the cCRE length). This conservative ≥50% overlap threshold ensures that TE sequences constitute the majority of each TE-derived cCRE and is consistent with previous studies examining TE-derived regulatory elements^16,20^.

### Logistic regression analysis of TE–cCRE overlap

To assess whether CREs are enriched or depleted for TE-derived sequences after accounting for potential confounders, we performed logistic regression analyses using statsmodels (v0.14) in Python. We fitted two types of models: (1) a cCRE-vs-background model comparing cCREs to randomly generated genomic regions, and (2) within-cCRE models examining the properties associated with TE overlap among cCREs. In all models, odds ratios and 95% confidence intervals were derived by exponentiating the model coefficients. All models were fitted using maximum likelihood estimation. The detailed results of these regression analyses are reported in Supplementary Data 1.

For the cCRE-vs-background model, we generated a set of size-matched (500 bp) random genomic background regions equal in number to the cCREs for each species by sampling uniformly from the genome, weighted by chromosome size. We then computed the following covariates for each region: GC content, mappability (mean *k* = 50 Umap multi-track mappability score^102^), and log-transformed distance to the nearest transcription start site (TSS). A binary TE overlap variable was defined as 1 if a region overlapped an annotated TE by more than 250 bp, and 0 otherwise. The logistic regression model was specified as: TE_overlap ~ is_cCRE + GC_content + mappability + log_dist_TSS, where is_cCRE is a binary indicator distinguishing cCREs from background regions.

For the within-cCRE models, we examined factors associated with TE overlap among cCREs only. The base model was specified as: TE_overlap ~ peakType + Cons_group_label + GC_content, where peakType denotes the genomic context of each cCRE (distal, intronic, exonic, or promoter) and Cons_group_label denotes its evolutionary conservation category of cCRE accessibility (species-specific, primate-specific, conserved, or others). We additionally fitted extended models that included the number of programs in which a cCRE is accessible (n_NMF) and the developmental stage at which the cCRE is first accessible (developmental state: embryonic, fetal, postnatal, or adult) as additional predictors.

For the analysis of the human whole-body chromatin accessibility atlas dataset^48^, cCREs were classified by their genomic location (distal, promoter proximal, or promoter) and by their developmental accessibility (adult-specific or fetal-specific; cCREs accessible in both stages were excluded). The logistic regression model was specified as: TE_overlap ~ C(Class) + C(accessibility_class) + GC_content + phyloP, where phyloP is the mean phyloP 447-way conservation score^86,88^. A TE overlap threshold of 200 bp was used for this dataset, consistent with the 400 bp cCRE size.

### Enrichment of TEs in cCREs

Enrichment of TEs in cCREs was computed as described in the previous study^16^. Briefly, we calculated the enrichment by dividing the fraction of overlaps between TE annotations and cCREs of interest (relative to the total number of cCREs of interest) by the fraction of genomic bases covered by the TE annotations of interest relative to the total genome size. For visualization purposes, we assigned a log_2_ enrichment value of −10 to TE subfamilies with no overlap with cCREs, which is lower than the enrichment value of any subfamily that does exhibit cCRE overlap.

When analyzing TE subfamily enrichment across program-specific cCREs, we first filtered out TE subfamilies with no more than ten cCREs across programs, retaining 322 of 902 subfamilies. After computing enrichment scores, we noticed that some TE subfamilies showing high enrichment signals have only a small number of overlaps with cCREs. For these low-abundance subfamilies, even a few overlaps can substantially inflate enrichment scores, making them unreliable. To address this, we implemented an additional filtering step requiring TE subfamilies with high enrichment scores (log_2_ enrichment > 2) to have more than 10 overlaps with program-specific cCREs. This filtering step excluded 31 TE subfamilies, resulting in 291 TE subfamilies for enrichment analysis across programs.

### Inferring the age of transposon subfamilies

We inferred the age of transposon subfamilies based on the divergence of individual genomic copies from the consensus sequence. Since each copy in the same subfamily accumulates mutations after its insertion, the divergence from its original form (inferred through consensus sequence reconstruction) increases over time. The level of copy divergence from the consensus sequence can be estimated by using the sequence distance computed using Kimura substitution model^103^. We used the Kimura divergence score of individual copies in the Dfam database, computed the median score for each subfamily and used this as a measure of the age of each TE subfamily.

### DeepCeREvo training and architecture

DeepCeREvo is a deep-learning model previously developed to predict CRE assignment to programs based on DNA sequence^39^. The model is a multiclass multilabel classifier with a hybrid convolutional and recurrent neural network architecture. The model takes 500 bp one-hot encoded DNA sequences as input, which are processed through: (i) a convolutional layer with 512 kernels (size 24), of which 285 were initialized with JASPAR 2020 motifs; (ii) a max-pooling layer; (iii) a time-distributed dense layer combined with a bidirectional LSTM layer (256 neurons); and (iv) final dense layers with sigmoid activation to predict probabilities for each program.

The original model was trained on a mixed human-mouse dataset containing highly variable CREs assigned to programs, supplemented with putatively inactive intergenic regions and lowly variable CREs, using an 80:10:10 training-validation-test split. Training employed data augmentation via sliding windows (500 bp windows, 50 bp stride) and was performed using the Adam optimizer with binary crossentropy loss. Model performance was evaluated using auROC and auPR metrics. In this study, we applied the trained DeepCeREvo model to systematically assess the regulatory potential of ancestral TE sequences (described in the section ‘*Assessing regulatory potential of TE subfamilies*’).

### Assessing regulatory potential of TE subfamilies

We assessed the regulatory potential of TE subfamilies using the deep-learning model DeepCeREvo, which predicts chromatin accessibility in specific cell states (programs) in cerebellum development by detecting combinations of cell-type-specific TF binding sites^39^. We obtained consensus sequences of all TE subfamilies in human and mouse using famdb.py (v1.0.2) on the FamDB HDF5 database (partition 0) from Dfam database (v3.8). We only considered TE subfamilies with at least 10 overlaps with highly variable cCREs in at least one cell group, resulting in 123 subfamilies. Since DeepCeREvo requires 500-bp input sequences, we created sliding windows of 500 bp with 100-bp steps to generate TE fragments for assessment.

Given that TE-derived cCREs are enriched in cell-type-specific elements, we developed an approach to compute the regulatory potential specific to one or a few cell groups. First, we transformed the DeepCeREvo outputs by applying the inverse sigmoid function (logit) and then the softmax function to convert values into a probability distribution across cell groups. This transformation enhances cell-type-specificity by generating high values for elements specific to certain cell types while assigning low values to elements with ubiquitously high accessibility. Next, we masked CpG dinucleotides from input sequences. This step was necessary because consensus TE fragments typically contain excess CpG dinucleotides compared to non-CpG-island genomic sequences, which is one of sequence features of promoters^104^. Finally, to account for sub-optimal proto-motif sequences in TE fragments, we computed regulatory potential by averaging transformed prediction scores from the original sequence and 25 sequences which had single nucleotide variants introduced to the original sequence and showed the highest regulatory potential. Specifically, we generated all possible single nucleotide variants of the original sequence, evaluated their regulatory potential, and selected the top 25 highest-scoring variants to include in our averaging calculation.

To determine statistical significance, we shuffled each TE fragment 1000 times and computed regulatory potential for these shuffled sequences. By comparing regulatory potential between original and shuffled fragments, we calculated empirical *P*-values and applied the Benjamini–Hochberg procedure to compute *Q*-values. Because our screening examines overlapping 500-bp windows tiled at 100-bp intervals along TE consensus sequences, adjacent fragments share 80% (400 bp) of their input sequence, resulting in highly correlated *P*-values between neighboring tests. Applying standard multiple testing correction to all tests would therefore be overly conservative, as many tests are not independent^105,106^.

To estimate the effective number of independent tests (${m}_{{eff}}$), we used the classical autocorrelation-based formula for temporally or spatially correlated data^107^, which has been applied to genomic analyses with similar correlation structure^106^. This method exploits the natural linear ordering of fragments along the TE consensus sequence and does not require multiple observations per test to estimate pairwise correlations. The formula is:

1 $${m}_{{eff}}=M/(1+2\Sigma \rho (k)),$$

where M is the total number of tests and ρ(k) is the autocorrelation at lag k. For each program, we ordered TE fragments by their position along the consensus sequence and computed the autocorrelation of −log_10_ *P*-values. Following standard practice, we summed autocorrelations over positive lags until the first non-positive value, which serves as a natural truncation point^108^. The resulting correction factors ($M/{m}_{{eff}}$) ranged from 1.6 to 7.4 across programs.

We incorporated ${m}_{{eff}}$ into the Benjamini–Hochberg procedure following the previous study^105^. Briefly, in the standard procedure, ordered *P*-values are compared against thresholds that increase linearly from α/M to α, where α is the target false discovery rate (FDR) level (e.g., 0.10 for 10% FDR). To account for correlated tests, we replaced the starting threshold α/M with $\alpha /{m}_{{eff}}$ while keeping the endpoint at α, thereby relaxing the correction to reflect the reduced number of truly independent tests. We called fragments significant at FDR < 20%, though eleven of the twelve identified TE subfamilies also meet the more stringent threshold of FDR < 10%.

### Computing positional enrichment of TE contributions to cCREs

We evaluated how much sequences derived from ancestral TE copies contribute to cell-type-specific cCREs. This analysis complements our regulatory potential screening, as we would expect TE fragments with high regulatory potential to contribute more significantly to cCREs of their corresponding cell types. To quantify this contribution, we first intersected highly variable cCREs with TE annotations using bedtools intersect (v2.30.0)^101^. We then mapped these intersections to corresponding consensus sequences using MAFFT (v7.505) with the --addfragments option^109^. For each TE fragment, we computed the number of bases aligned to cCREs and counted it as an overlap if ≥ 100 bases were aligned. To establish background expectations, we applied the same procedure to a chromatin accessibility atlas containing cell types from the whole human body at both fetal and adult stages^48^. Finally, we calculated positional enrichment of each TE fragment by dividing the proportion of highly variable cCREs in cerebellum development overlapping each TE fragment by the proportion of background cCREs overlapping the same fragment.

### In silico screening for TE subfamilies co-opted due to high regulatory potential

To identify TE subfamilies that have been co-opted as regulatory elements during cerebellar development, we combined two complementary metrics: regulatory potential assessed by DeepCeREvo (after FDR correction at 10% or 20%) and positional enrichment of TE contributions to highly variable cCREs. We identified 500-bp windows that showed both statistically significant regulatory potential for specific cell types and enriched overlap with cCREs at corresponding positions in extant copies. This combined approach identified seventeen non-overlapping 500-bp window–program combinations from twelve TE subfamilies as candidates for co-option as cell-type-specific regulatory elements. For simplicity, we focused on one 500-bp window–program combination per TE subfamily by selecting the combination with the highest −log_10_ *Q*-value. The regulatory potential and other screening results for all 500-bp window–program combinations are described in Supplementary Data 3.

### Mapping chromatin accessibility to the consensus sequences and identifying accessible copies of the screened TE subfamilies

To analyze the chromatin accessibility patterns of the screened TE subfamilies, we first constructed a multiple sequence alignment (MSA) for each subfamily by aligning all annotated copies in the human genome to their respective consensus sequence using MAFFT (v7.505) with the --addfragments option^109^. For each TE copy, we mapped chromatin accessibility data from various cell types to its corresponding position in the MSA using pyBigWig (v0.3.18) on accessibility tracks in bigWig format, enabling us to generate consensus-based accessibility profiles for each subfamily across different cell types. To identify copies with accessible chromatin at a defined region of interest within the consensus sequence in the corresponding cell states, we computed the total accessibility score for each copy within that region. Based on the distribution of these scores, we established a subfamily-specific threshold to classify TE copies as accessible or inaccessible in this cell type.

### Accessibility of the screened TE subfamilies across developmental stages and brain regions

To investigate chromatin accessibility patterns of TE subfamilies throughout brain development and across different neuroanatomical regions, we integrated data from multiple sources. We obtained chromatin accessibility tracks for cell types in the first trimester of human brain development from the CATlas database^48,61^. Recognizing that different brain regions develop at varying rates, with hindbrain structures typically maturing earlier than other regions, we incorporated additional chromatin accessibility datasets spanning the second trimester to adult stages of cerebral cortex development^73,74^. Since the accessibility data from Herring et al.^74^. were originally mapped to the hg19 assembly, we converted these files to hg38 coordinates using CrossMap (v0.7.0)^110^ to ensure consistency across all analyses. For each cell type represented in these datasets, we computed relative accessibility profiles of TE copies based on their alignment positions within the corresponding consensus sequence, following the same approach described above.

To quantify uncertainty in the mean accessibility profiles, we employed a bootstrap resampling approach. For each cell type, we selected the top 50 copies of each TE subfamily ranked by mean accessibility within the region of interest. We then resampled these 50 copies with replacement 1000 times, computing the mean accessibility profile across the consensus for each bootstrap iteration. The 95% confidence interval at each position was defined by the 2.5th and 97.5th percentiles of the resulting bootstrap distribution. All profiles and confidence intervals were smoothed using a centered rolling mean with a window size of 25 bp.

### Constructing phylogenetic tree of HERVL-related TE

We constructed a multiple sequence alignment (MSA) of consensus sequences from the ERV-int region of 14 TE subfamilies: 12 from the human ERVL family plus MERVL and MERVL-2A from mouse, using MAFFT^108^. We identified regions homologous to positions 2000–2500 nt of the HERVL consensus and retained only subfamilies with ≥250 bp overlap in this region. Using these aligned sequences, we constructed a refined MSA, removed poorly aligned regions using trimAl (v1.4) with the -gappyout parameter^110^ and built a phylogenetic tree using IQ-TREE2 (v2.3.4) with default parameters^111^. We excluded 6 subfamilies (HERVL74, ERVL40, HERVL66, HERVL32, HERVL1, and ERVL47) that showed high sequence divergence from HERVL and repeated the tree construction to obtain the final phylogenetic tree of 8 closely related subfamilies: HERVL, ERVL, ERVL-B4, ERVL-E, HERV16, ERV3-16A3-I, MERVL, and MERVL-2A. Trees were visualized using treeio (v1.18.1) and ggtree (v3.2.1)^112,113^.

### Constructing phylogenetic tree of HERVL-related TE copies in the human genome

To construct a phylogenetic tree of individual TE copies, we obtained genomic coordinates of the 6 human HERVL-related subfamilies identified above (HERVL, ERVL, ERVL-B4, ERVL-E, HERV16, and ERV3-16A3-I). For the multi-subfamily tree, we retained only annotated copies >2000 bp to ensure analysis of structurally intact elements, and required ≥400 bp overlap with the HERVL 2000–2500 nt region for reliable phylogenetic inference. We constructed MSAs and phylogenetic trees using the same methodology as for consensus sequences. For a HERVL-specific phylogenetic analysis, we applied more lenient criteria to increase sample size: we included annotated HERVL copies >250 bp with ≥250 bp overlap with the HERVL 2000–2500 nt region. The phylogenetic tree was constructed using the same pipeline.

### Luciferase reporter assays

Reporter assays were performed and analyzed essentially as described previously^39^. In all experiments, granule cells cryopreserved in CryoStor CS10 (Stemcells Technologies) after pre-culturing, were used. To obtain the cryopreserved cell stocks, P7 pups of RjOrl:SWISS mice (Janvier Labs) were sacrificed by decapitation, cerebella were dissected, cells were dissociated using Papain Dissociation System Kit (Worthington), subjected to 35%/60% Percoll density gradient centrifugation, and pre-cultured for 2 times 45 min on uncoated cell culture plates to remove the highly adherent glial cells^39^. The sex of the pups was not tracked or analyzed as a variable, as the experiments measured sequence-specific regulatory activity in vitro, which is not expected to vary by sex. All pups from each litter (12–18 pups per litter) were used to generate the cryopreserved stocks. After thawing, granule cells were plated on 96-well plates coated with poly-D-lysine (0.1 mg/ml, Thermo Fisher Scientific) and Matrigel Growth Factor Reduced Basement Membrane Matrix (Corning) diluted 1:75 in HBSS (Thermo Fisher Scientific) at a density of 1.5–3 × 10^5^ live cells/cm^2^. Cells were cultured for 3 days in vitro (DIV) in Neurobasal Plus Medium supplemented with 100 U/ml penicillin-streptomycin, 2 mM GlutaMAX, 4.5 g/l glucose, B-27 Plus (all Thermo Fisher Scientific), SPITE and Linoleic Acid-Oleic Acid-Albumin supplements, 0.16 mg/ml N-Acetyl-L-cysteine, and 200 nM InSolution Smoothened Agonist (all Sigma-Aldrich).

For HERVL and 5 human TE subfamilies closely related to HERVL (ERVL, ERVL-B4, ERVL-E, HERV16, ERV3-16A3-I), as well as mouse MERVL, consensus sequences corresponding to the 2000–2500 nt region of HERVL were synthesized. MERVL-2A was not included due to its high sequence similarity to MERVL. As a positive control, we included a region nearby *E2F1* (hg38_chr20:33685056-33685556), which was previously shown to have enhancer activity in this experimental system^39^. As negative controls, we included dinucleotide-shuffled versions of both the HERVL consensus sequence and the *E2F1* enhancer sequence. Sequences with suitable homology arms were synthesized as eBlocks at IDT and cloned into pNL1.2[NlucP]-based (Promega) vectors–pNL1.2_adaptersF_SV40_NlucP and pNL1.2_adaptersR_SV40_NlucP^39^–upstream of an SV40 promoter in both forward and reverse orientations, using In-Fusion Snap Assembly Master Mix (Takara). Cloning of ERVL-E and ERV3-16A3-I in the reverse orientation failed repeatedly and they were excluded from subsequent analyses. All constructs were verified by Sanger sequencing (Azenta Life Sciences).

Cells were transfected at DIV2 using FuGENE HD (Promega) with 95 ng of test constructs and 30 ng of firefly luciferase normalizer plasmid pGL4.15[luc2P/EF1α/Hygro]. Luciferase activities were measured 28–30 h post-transfection using Passive Lysis Buffer and the Nano-Glo Dual-Luciferase Reporter Assay System (Promega). Data were collected from 3 to 6 independent experiments, each conducted in triplicate, with data from at least two independent experiments retained after quality control (Supplementary Data 4), which excluded wells with firefly luciferase signals less than 3-fold above background. Signals from untransfected wells were subtracted, NanoLuc values were normalized to firefly signals, and ratios were log_2_-transformed. To standardize across experiments, values were scaled using experiment-specific standard deviations and adjusted to a common global spread. Statistical analysis was performed using linear mixed models with R packages lme4 (v1.1-36)^114^, lmerTest (v3.1-3)^115^, and pbkrtest (v.0.5-0.1)^116,117^, with element, orientation, and their interaction as fixed variables and independent experiments as random variables. The data did not support inclusion of a random intercept for experiment (singular fit), resulting in a model equivalent to a fixed-effects linear model. *P*-values were corrected for multiple comparisons using the Benjamini–Hochberg method. All sequences and values are provided in Supplementary Data 4.

### Regression analysis of sequence conservation at high attribution sites

To test whether sequences at high-attribution sites are significantly more preserved in accessible copies relative to inaccessible copies, we performed a linear regression analysis. For each TE subfamily, we first computed per-position attribution scores on the ancestral consensus sequence using DeepExplainer with 100 shuffled background sequences. Positions were classified as high-attribution (attribution score ≥ 90th percentile) or low-attribution (absolute attribution score ≤ 90th percentile). Sequence conservation at each position was defined as the proportion of copies matching the ancestral nucleotide, and the conservation difference between accessible and inaccessible copies was computed as the outcome variable. We then fit an ordinary least squares regression model with the conservation difference as the dependent variable, a binary indicator for high- versus low-attribution positions as the primary predictor, and one-hot encoded nucleotide identity as a covariate to control for base composition effects. A significant positive coefficient for the high-attribution indicator would indicate that accessible copies preferentially preserve ancestral sequences at positions with high model attribution compared to positions with low attribution.

### KZFP binding site analysis

We used a public database on KZFP binding sites from chromatin immunoprecipitation and lambda exonuclease digestion (ChIP-exo) experiments on HEK293T cell lines overexpressing different KZFPs with a hemagglutinin (HA) tag^118,119^. For each TE subfamily, we obtained the list of KZFPs whose binding sites are enriched in copies containing the region of interest (padj.binomial < 0.05). KZFPs that are barely expressed (CPM < 10) in the corresponding cell type were excluded. ChIP-exo peaks for each remaining KZFP were intersected with the coordinates of the relevant TE copies. KZFPs with fewer than 5 binding sites within these copies were also excluded from further analysis. We then counted the number of TE copies with different features that have binding sites for each KZFP and computed the fraction.

### Single cell chromosomal compartment analysis

We analyzed the chromatin compartmentalization of TE elements in human cerebellar development using single-cell chromatin A/B compartment (scA/B) datasets obtained from^120^. A/B compartments represent regions of open euchromatin and closed heterochromatin, respectively. For each TE subfamily, we determined the midpoints of the annotated copies and extracted the mean scA/B score across all five granule cell structural stages (S1–S5) at each midpoint location.

### Local chromatin environment analysis

To investigate how the local chromatin environment influences TE copy accessibility, we analyzed the spatial relationship between TE copies and cCREs. For each TE subfamily, we focused on cCREs specifically accessible in the corresponding program that did not overlap with any annotated TEs ≥ 250 bp in length. For each TE copy, we determined the distance to the nearest non-TE-derived, program-specific cCRE using bedtools closest (v2.30.0) with the pybedtools wrapper (v0.9.0)^101,121^.

To characterize the broader regulatory landscape, we calculated the density of these non-TE-derived cCREs around TE copies using bedtools window (v2.30.0) with a window size of 250 kb^101,121^. We divided this window into 50 kb bins and counted the number of cCREs in each bin. The density was calculated by dividing these counts by the number of TE copies in each class, providing a normalized measure of regulatory element distribution around different categories of TE elements.

### Logistic regression analysis of TE copy accessibility

To assess the relative contribution of multiple genomic features to TE copy accessibility, we performed logistic regression for each TE subfamily using statsmodels (v0.14) in Python. The dependent variable was a binary indicator of whether a given TE copy is accessible in the corresponding cell type (i.e., overlaps a cCRE in the relevant program). The independent variables included: GC content of the TE copy sequence, copy length, sequence divergence from the consensus (Kimura divergence), predicted accessibility score from DeepCeREvo, log_10_-transformed distance to the nearest TSS, log_10_-transformed distance to the nearest non-TE-derived program-specific cCRE, the presence of binding sites for each KZFP with enriched binding in the subfamily, and the mean single-cell A/B compartment score across granule cell structural stages. Odds ratios and 95% confidence intervals were derived by exponentiating the model coefficients. All models were fitted using maximum likelihood estimation.

### Analysis of gene expression in proximity to the screened TE subfamilies

To examine the potential regulatory influence of TE insertions on nearby genes, we assessed the relationship between TE accessibility and the expression of genes within a 500 kb window (±250 kb) centered on each TE copy. We extended the center position of each element using bedtools slop (v2.30.0) and identified genes with transcription start sites (TSS) within this window using bedtools intersect (v2.30.0). For each identified gene, we computed the mean expression level across all samples classified as differentiating granule cells (GC_diff_1), enabling us to evaluate whether accessible TE copies were associated with differential expression of neighboring genes.

### Identification of orthologous TE copies between species

To investigate the evolutionary impact of TE insertions on chromatin accessibility and gene expression in differentiating granule cells across primate lineages, we identified orthologous TE elements between species. Using the UCSC liftOver tool with default parameters, we mapped human TE genomic coordinates to their corresponding 1:1 orthologous regions in macaque, marmoset, and mouse genomes. We then used bedtools intersect (v2.30.0) to determine which of these syntenic regions contained annotated copies belonging to the same TE subfamily in the target species^101^. To robustly define orthology, we retained only elements that were explicitly annotated as members of the same TE subfamily in the non-human genome and had a length at least 20% of that of their human counterparts, thereby filtering out severely truncated or degraded elements.

### Evaluation of the contributions of species-specific TE copies to cross-species gene expression divergence

To investigate whether species-specific accessible TE insertions contribute to evolutionary divergence in gene expression patterns, we analyzed a set of 1:1:1:1 orthologous protein-coding genes conserved across human, macaque, marmoset, and mouse genomes. We quantile-normalized the mean expression values of these genes in differentiating granule cells (GC_diff_1) across all four species to facilitate direct cross-species comparisons. To identify genes potentially influenced by human-specific TE insertions, each TE copy was associated with the most highly expressed gene within a predefined genomic window. Because gene density and transcriptional activity varied among loci, window sizes were adjusted to limit the inclusion of distant, lowly expressed genes that were unlikely to represent direct regulatory targets. For HERVL, a ±250 kb window was used to focus on the local genomic neighborhood and improve the specificity of candidate-gene assignment. For LTR1A2 and MER52D, a broader ±1 Mb window was used to ensure that candidate genes were captured in loci with sparser gene distributions or where nearby genes were weakly expressed. As a result, candidate-gene assignments were determined independently for each TE subfamily, and the resulting statistics should not be interpreted as directly comparable across subfamilies.

### Reporting summary

Further information on research design is available in the Nature Portfolio Reporting Summary linked to this article.

## Supplementary information

Supplementary Information file

Description of Additional Supplementary Files

Supplementary Data 1

Supplementary Data 2

Supplementary Data 3

Supplementary Data 4

Supplementary Data 5

Supplementary Data 6

Supplementary Data 7

Reporting Summary

Transparent Peer Review file

## Source data

Source Data file

## Data Availability

Previously published datasets used in this study are available in the ArrayExpress database under accession codes E-MTAB-9765 and E-MTAB-10533^37^, and in the heiData repository under accession codes QDOC4E^38,122^ and GDSZG9^39,123^. Source data are provided with this paper.

## Code availability

All original code is available on GitLab [https://gitlab.com/kaessmannlab/cerebellum_te]. An archived snapshot corresponding to the manuscript is deposited on Zenodo [10.5281/zenodo.19354896]^124^.
