# Supplementary material for: Gene regulatory innovations from transposable elements in primate cerebellum development
Source: Nat Commun. 2026 Jul 30;17:7598. doi: 10.1038/s41467-026-75700-7 (PMC13421694; doi:10.1038/s41467-026-75700-7)
Supplement: Supplementary file 1 — Supplementary Information file [file 41467_2026_75700_MOESM1_ESM.pdf]

Supplementary Information for:

Gene regulatory innovations from transposable elements in primate cerebellum development

Tetsuya Yamada[1]*, Mari Sepp[1,2], Ioannis Sarropoulos[1,3,4,5]*, Henrik Kaessmann[1,5]*

[1]Center for Molecular Biology of Heidelberg University (ZMBH), DKFZ-ZMBH Alliance, Heidelberg, Germany.
[2]Present address: Centre of Genomics, Evolution and Medicine (cGEM), Institute of Genomics, University of Tartu, Tartu, Estonia.
[3]Present address: Wellcome Sanger Institute, Cambridge, UK.
[4]Present address: Cambridge Stem Cell Institute and Department of Medicine, University of Cambridge, Cambridge, UK.
[5]These authors jointly supervised this work: Ioannis Sarropoulos, Henrik Kaessmann.

*Correspondence:
t.yamada@zmbh.uni-heidelberg.de; is606@cam.ac.uk; h.kaessmann@zmbh.uni-heidelberg.de

The PDF file includes:

- Supplementary Figures 1–20
- Supplementary References

Supplementary Figures

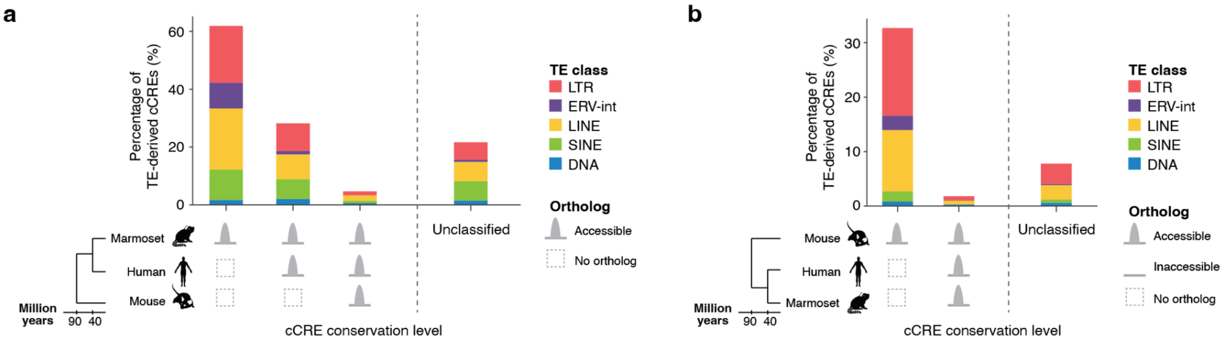

Supplementary Fig. 1 | Transposable element contributions to cCREs during marmoset and mouse cerebellar development. a, b, Percentage of TE-derived cCREs during marmoset (**a**) and mouse (**b**) cerebellar development across accessibility conservation categories between human, marmoset, and mouse.

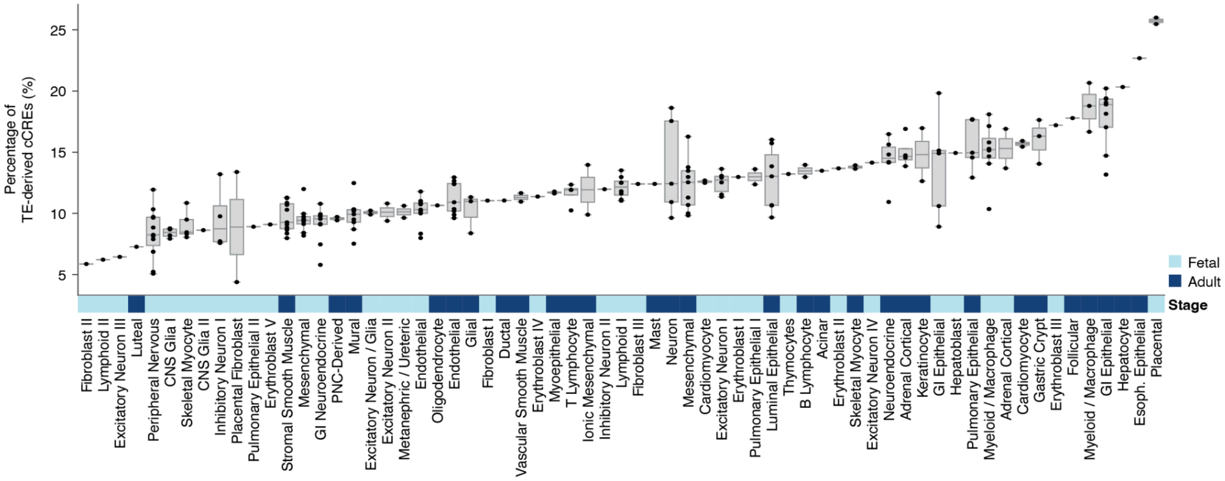

Supplementary Fig. 2 | Fraction of TE-derived cCREs varies across fetal and adult human cell types. Percentage of TE-derived cCREs across individual cell types (subclusters, dots) grouped by major cell type clusters. Samples are separated into fetal and adult developmental stages. Data from Zhang et al., 2021[1] reanalyzed. Source data are provided as a Source Data file.

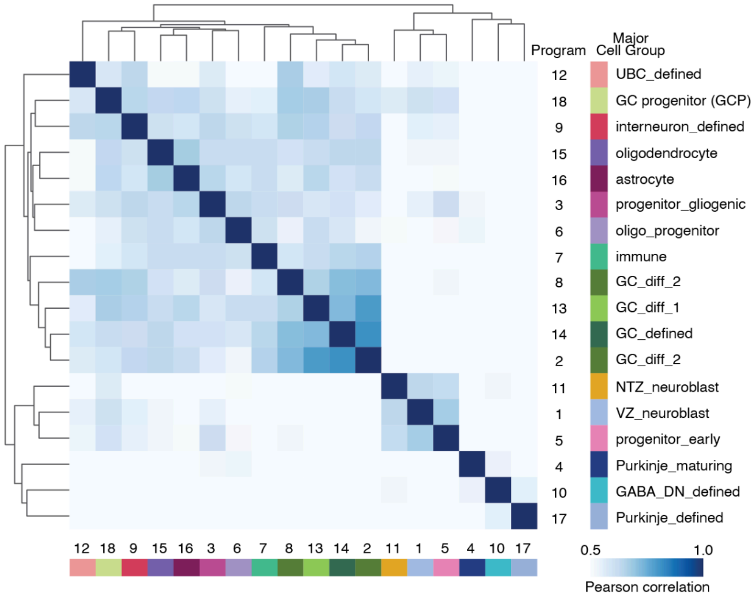

Supplementary Fig. 3 | Pairwise correlation analysis of TE subfamily enrichment in program-specific cCREs. Pairwise correlation matrix of $\log_2$ enrichment of TE overlaps with cCREs specific to individual programs. Rows and columns are ordered by hierarchical clustering of the correlation matrix.

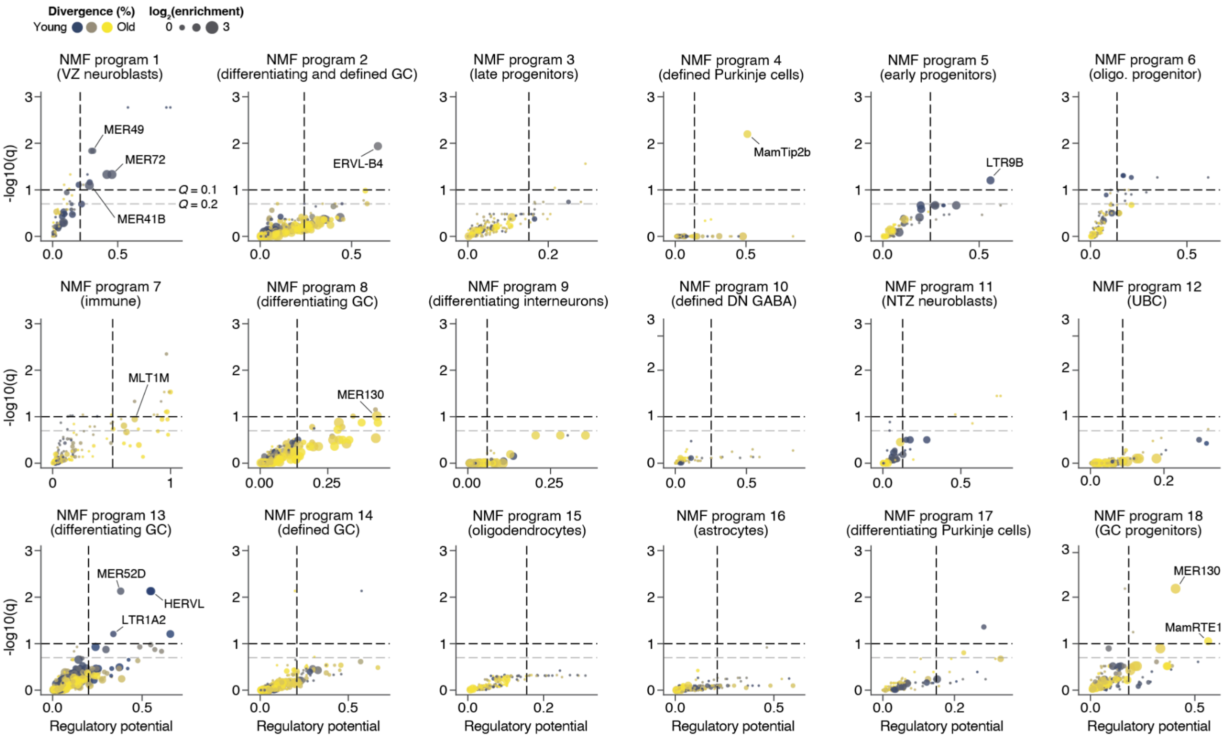

Supplementary Fig. 4 | *In silico* screening of transposable element fragments with high regulatory potential in human cerebellar development. Screening results for TE fragments showing regulatory potential scores and associated $-\log_{10}$ Q-values (P-values corrected by Benjamini–Hochberg procedure), alongside enrichment in cell-type-specific cCREs for each program. The regulatory potential threshold was set at the 95th percentile. The Q-value threshold was set at 0.10 and 0.20, corresponding to a false discovery rate (FDR) of 10% and 20%, respectively. The screened TE subfamilies are highlighted by the fragment with the highest $-\log_{10}$ Q-value. VZ, ventricular zone; NTZ, nuclear transitory zone; GC, granule cell; UBC, unipolar brush cell; DN GABA, deep nuclei GABAergic neuron. Source data are provided as a Source Data file.

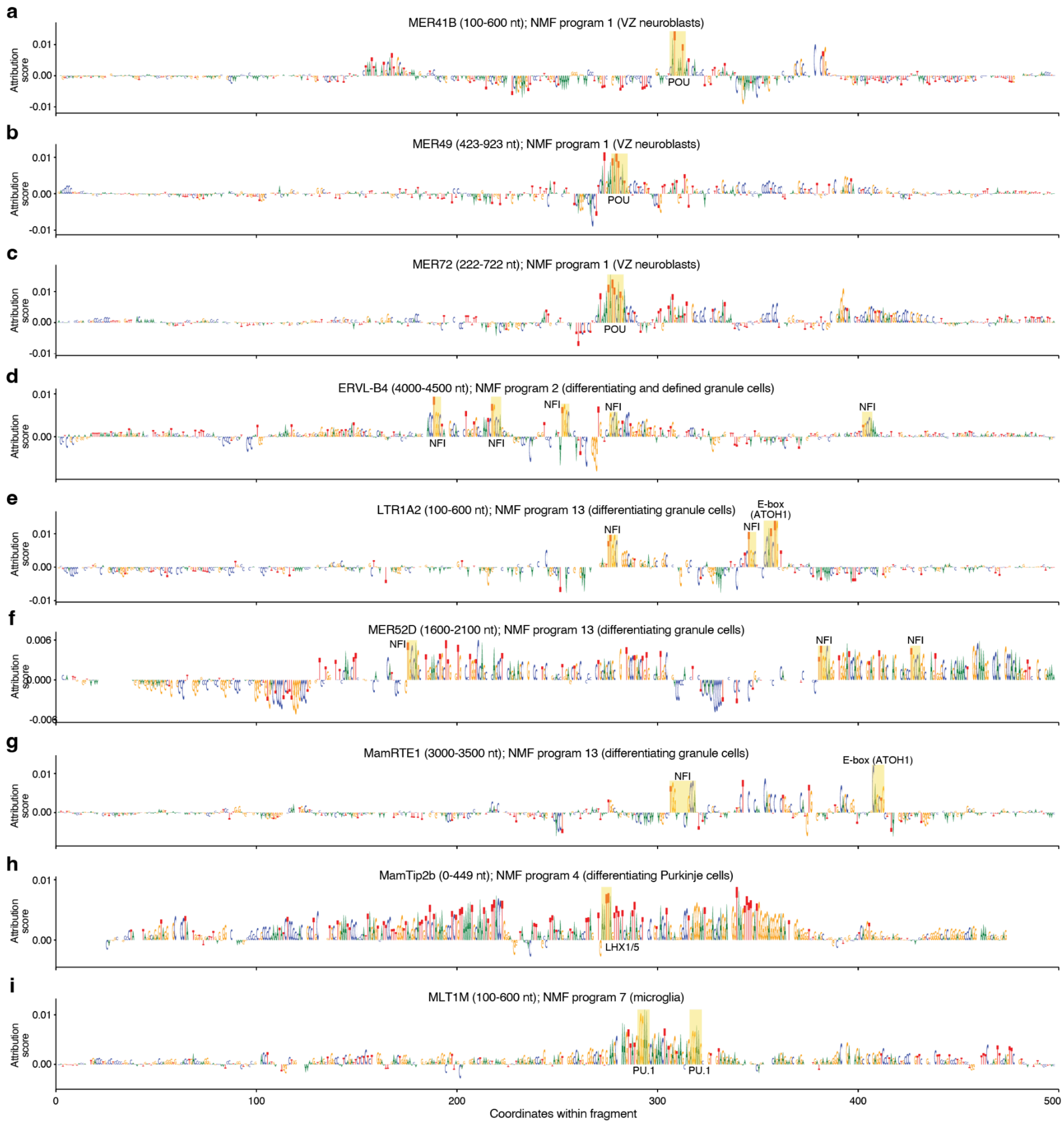

Supplementary Fig. 5 | Attribution profiles of consensus sequences from the screened TE subfamilies. a–i, DeepExplainer attribution profiles of the consensus sequences from each screened TE subfamily highlighted in Supplementary Fig. 4.

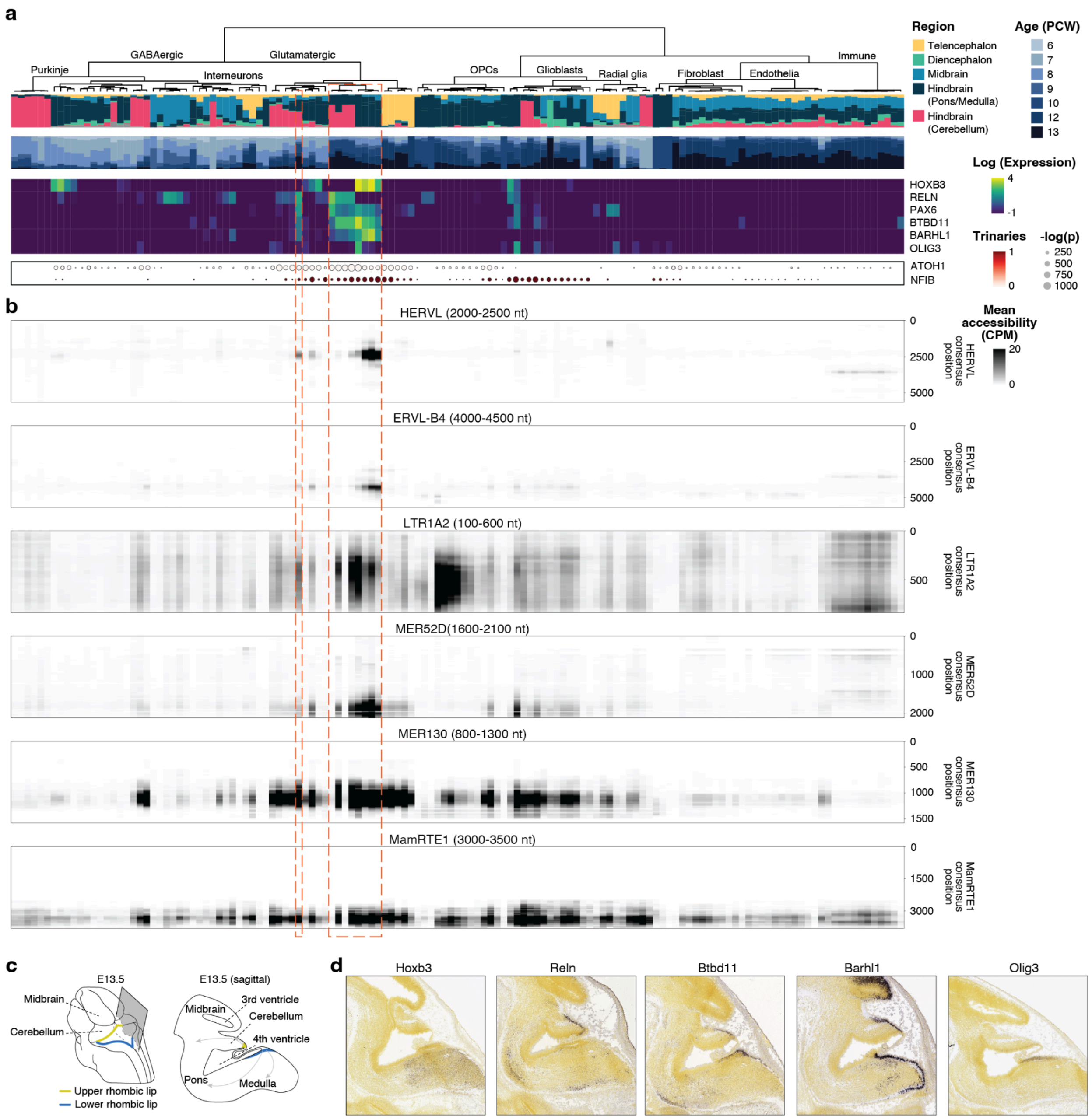

Supplementary Fig. 6 | Hindbrain glutamatergic neuroblast-specific accessibility of the screened TE subfamilies. a, Top to bottom: hierarchical clustering dendrogram showing relationships between cell types based on highly variable cCREs; anatomical region distribution showing the proportion of each cell type across brain regions; developmental age distribution showing temporal profiles of each cell type; expression heatmap of selected marker genes across cell types; TF binding motif enrichment analysis with dot size representing statistical significance (*P*-values) and color intensity representing expression levels of the corresponding TFs (trinarization score represents a probabilistic measure of gene expression ranging from 0-1). Highlighted cell types show HERVL accessibility (Fig. 3d). Data from Mannens et al., 2024[2] reanalyzed. **b**, Mean accessibility profiles of the 50 most accessible copies of each of the six TE subfamilies identified as specifically co-opted in the granule cell lineage (Supplementary Fig. 4), across

diverse cell types from different brain regions at various developmental stages (data from Mannens et al., 2024[2]), aligned to the consensus sequence of the respective TE subfamily. **c,** Schematic view of a mouse embryo highlighting the cerebellum at E13.5 (left) and sagittal section of the hindbrain along the plane indicated on the left (right). Arrows indicate the migration paths of rhombic-lip-derived neuroblasts. **d,** *In situ* hybridization images from the Allen Developing Mouse Brain Atlas[3] (https://developingmouse.brain-map.org) showing expression of marker genes in E13.5 mouse hindbrain. OPC, oligodendrocyte precursor cell.

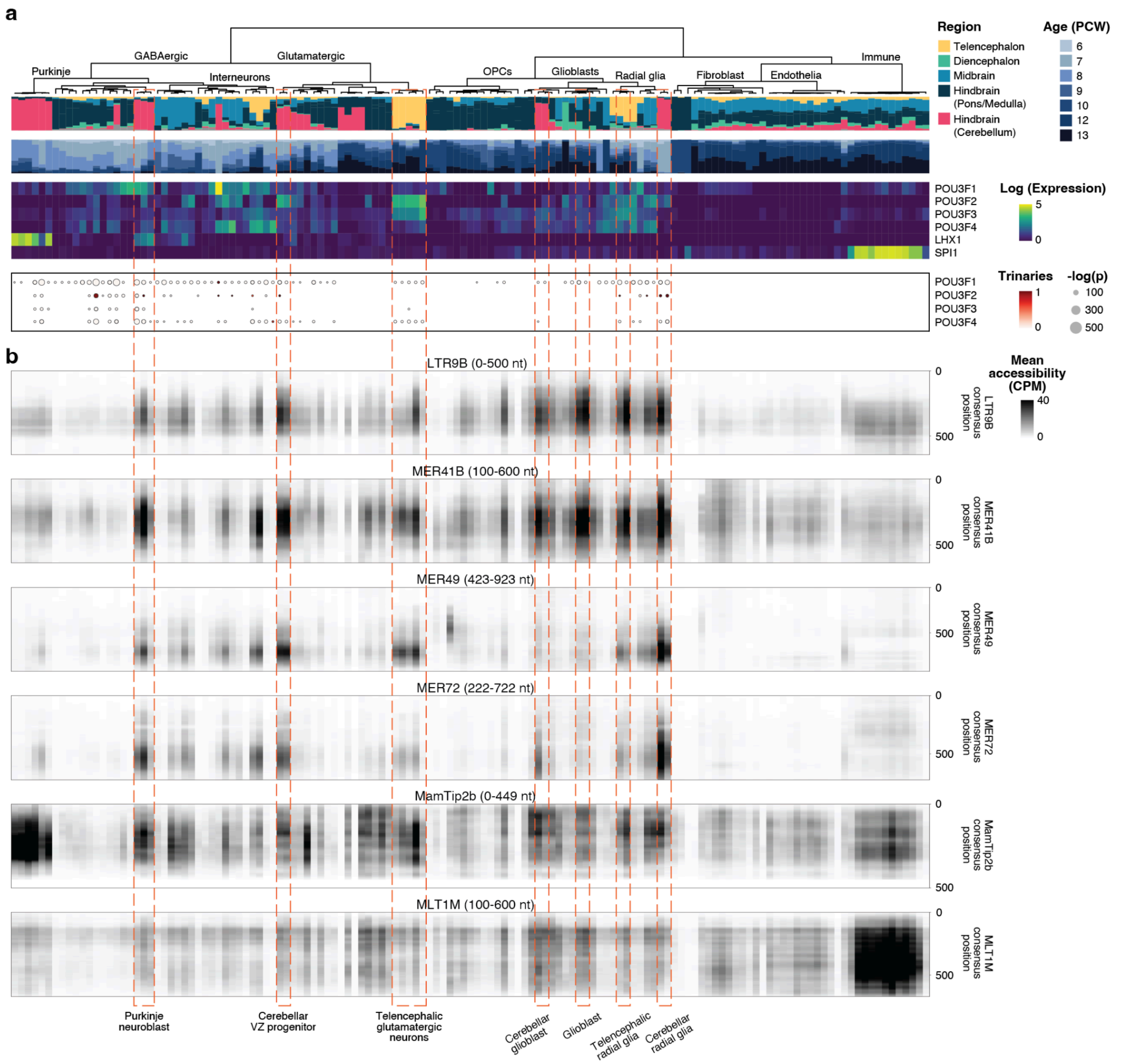

Supplementary Fig. 7 | Lineage-specific accessibility of the screened TE subfamilies. a, Cell type characterization as in Supplementary Fig. 6a. **b**, Mean accessibility profiles of the 50 most accessible copies of each of the six TE subfamilies identified as specifically co-opted in neural progenitors/neuroblasts (LTR9B, MER41B, MER49, and MER72), Purkinje cells (MamTip2b), and microglia (MLT1M) (Supplementary Fig. 4), across diverse cell types from different brain regions at various developmental stages (data from Mannens et al., 2024[2]), aligned to the consensus sequence of the respective TE subfamily.

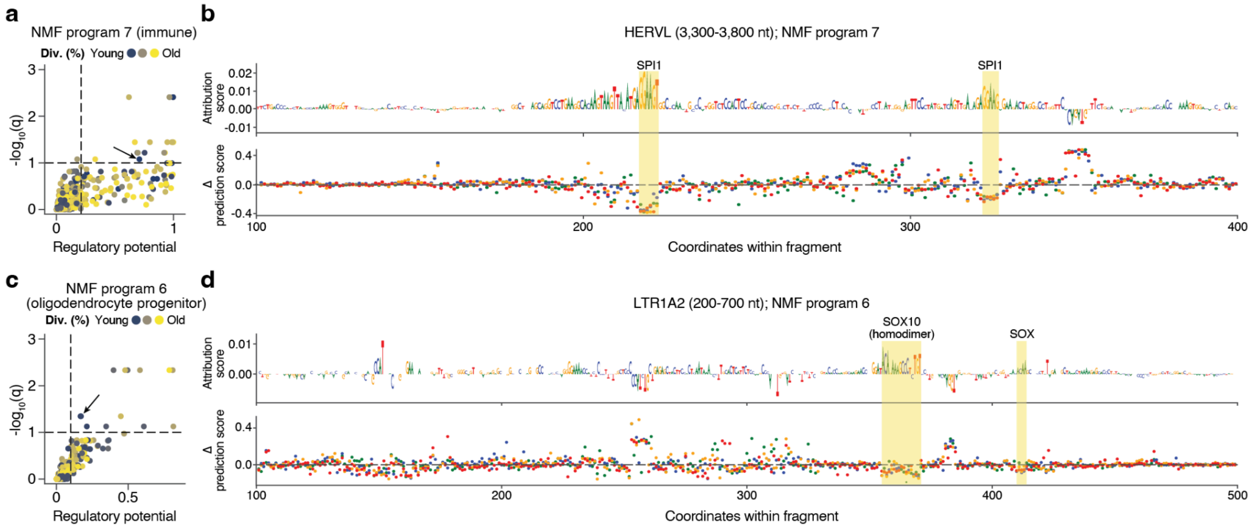

Supplementary Fig. 8 | Sequence features explaining increased accessibility in distinct cell types. a, c, Regulatory potential scores and associated *Q*-values in microglia (program 7) (**a**) and oligodendrocyte progenitors (program 6) (**c**) for all TE fragments. In contrast to Supplementary Fig. 4, TE fragments with fewer than 10 overlaps with highly variable cerebellar cCREs are also shown. **b, d,** DeepExplainer attribution profiles in microglia for HERVL at the 3,300–3,800 nt position (**b**) and in oligodendrocyte progenitors for LTR1A2 at the 200–700 nt position (**d**), which are indicated by an arrow in (**a)** and (**c**), respectively.

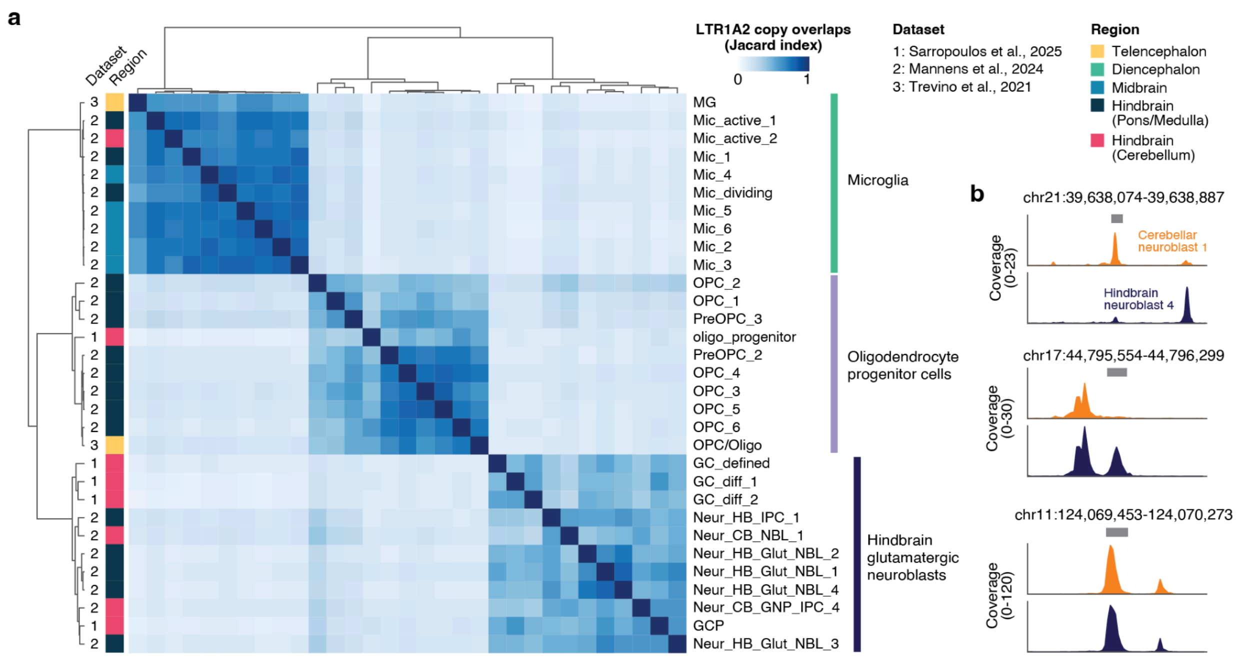

Supplementary Fig. 9 | Heterogeneity of LTR1A2 accessibility at the copy level. a, Pairwise Jaccard similarity index matrix of the 50 most accessible LTR1A2 copies across cell types. **b,** Examples of LTR1A2-overlapping cCREs specific to cerebellar neuroblasts 1 (Neur_CB_NBL_1; top), hindbrain glutamatergic neuroblasts 4 (Neur_HB_Glut_NBL_4; middle), or shared between these two cell types (bottom). Annotated LTR1A2 copies are shown as gray bars.

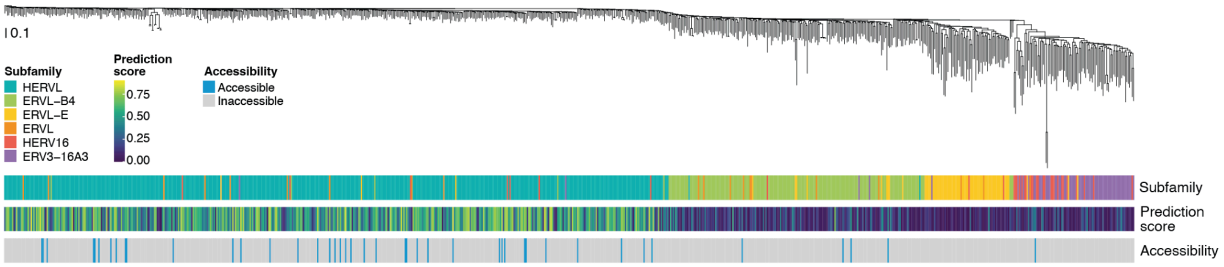

Supplementary Fig. 10 | Phylogenetic relationships of HERVL-related subfamily copies in the human genome. Phylogenetic tree of 852 copies belonging to subfamilies closely related to HERVL, constructed using regions orthologous to the HERVL 2,000–2,500 nt position. For each copy, the annotated subfamily, DeepCeREvo prediction score for program 13 (differentiating granule cells), and accessibility in differentiating granule cells (GC_diff_1) are shown.

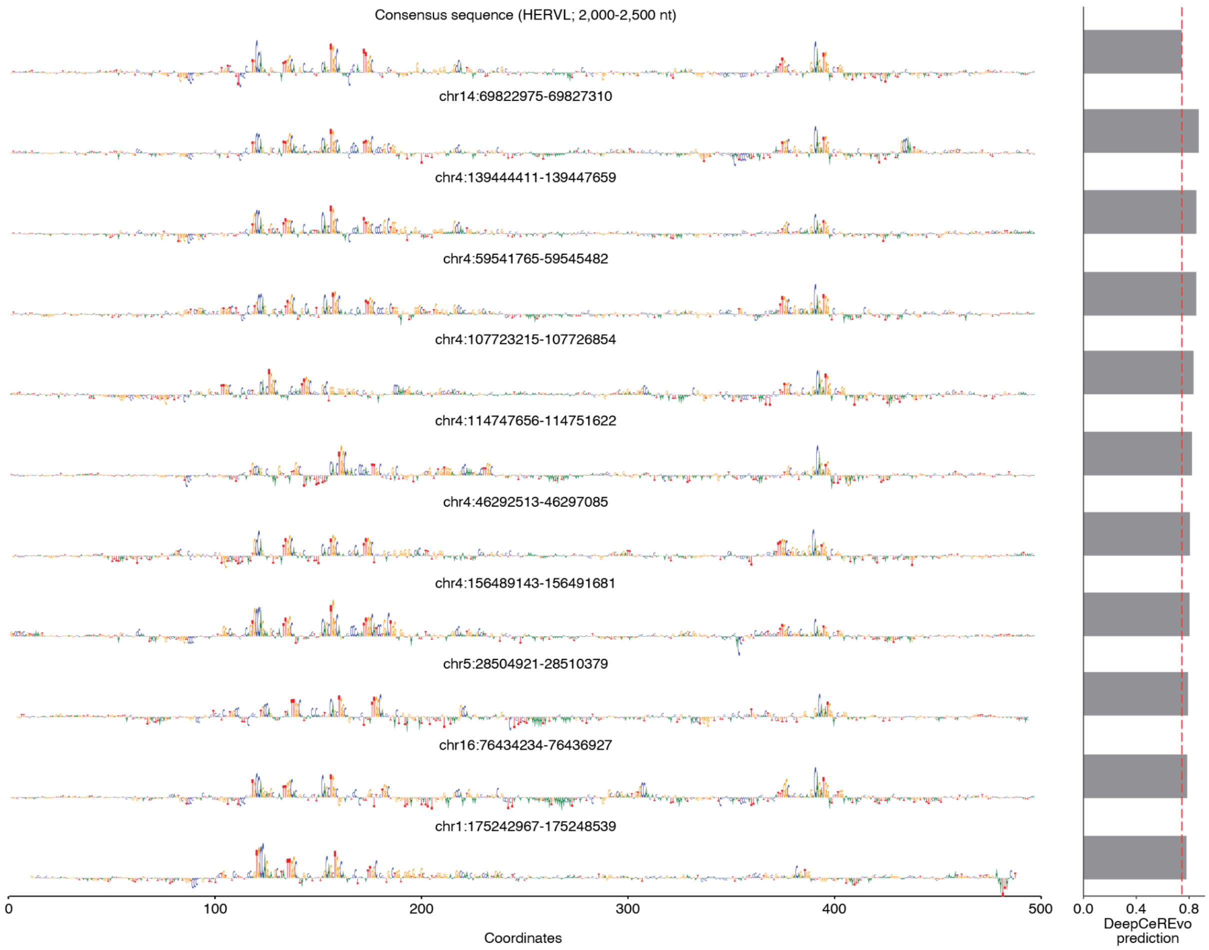

Supplementary Fig. 11 | Example attribution profiles of accessible HERVL copies in the human genome. DeepExplainer attribution profiles (left) and DeepCeREvo prediction scores (right) for the HERVL consensus sequence and the 10 HERVL copies accessible in differentiating granule cells (GC_diff_1) with the highest prediction scores. The DeepCeREvo prediction score of the consensus sequence is indicated for reference (dotted red line).

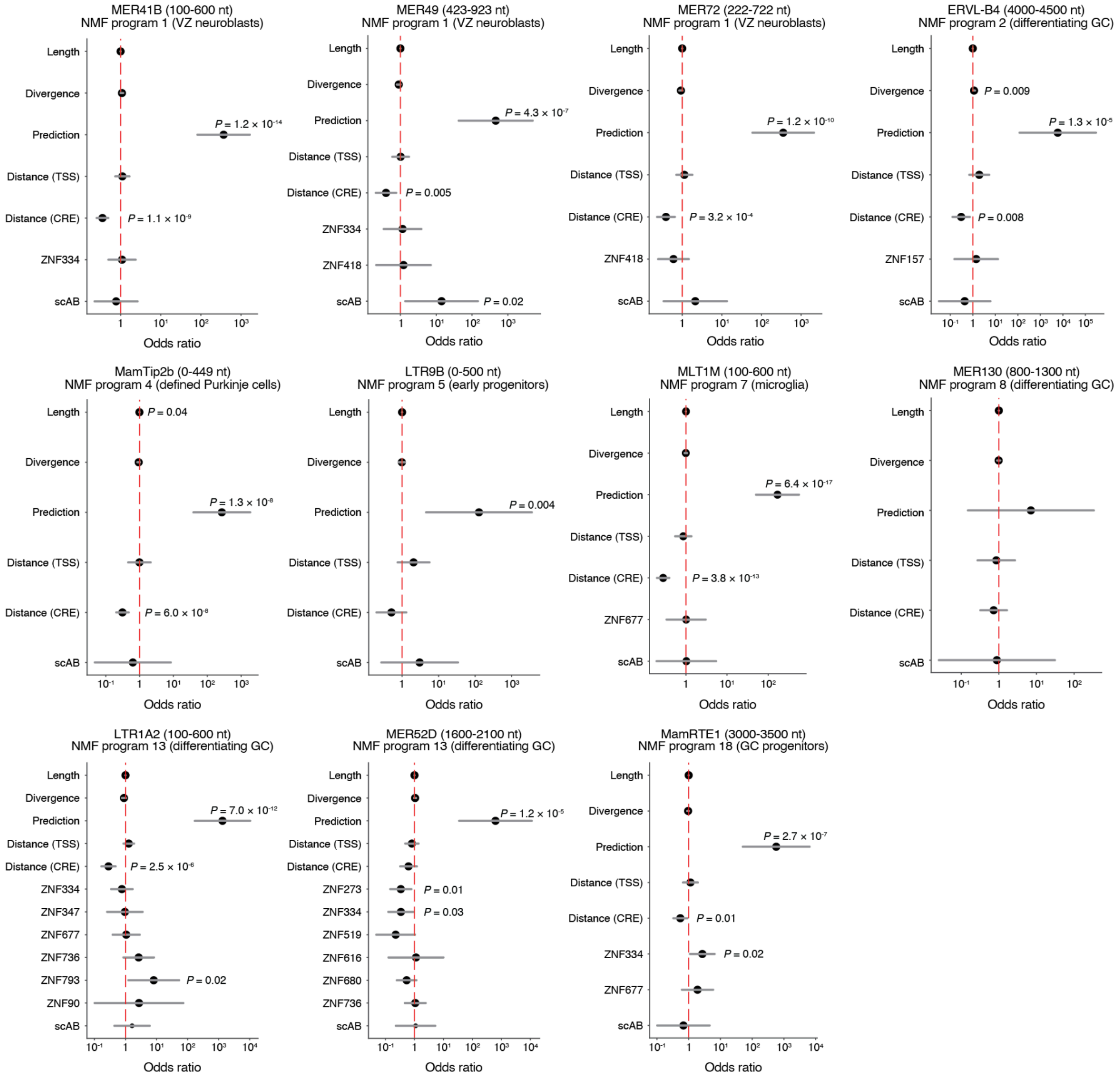

Supplementary Fig. 12 | Determinants of copy-level TE co-option for the screened TE subfamilies. Point estimates of odds ratios and 95% confidence intervals for each covariate in logistic regression models predicting accessibility of individual copies of the individual screened TE subfamilies. *P*-values for individual covariates were estimated using two-sided Wald tests. No adjustments were made for multiple comparisons.

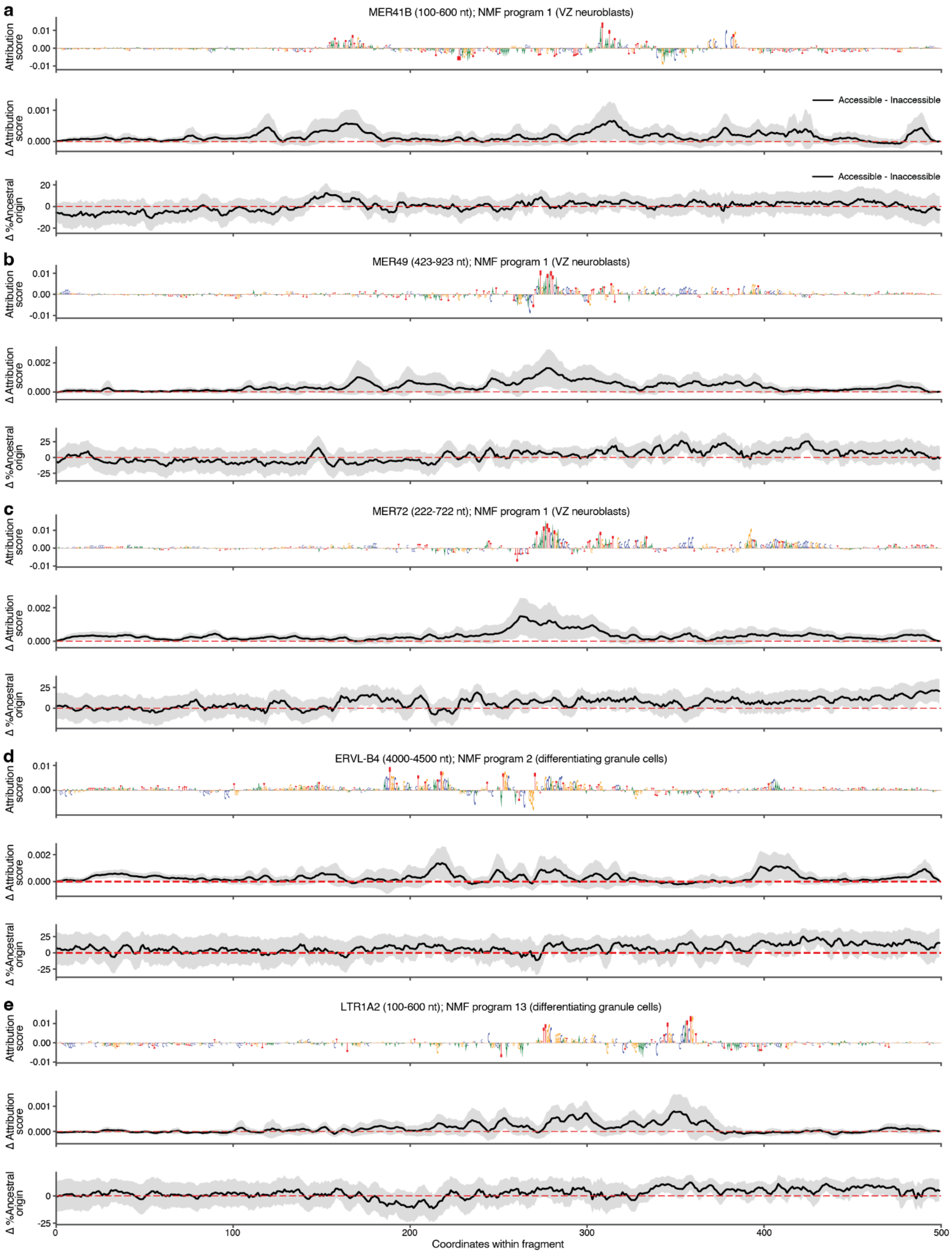
a
MER41B (100-600 nt); NMF program 1 (VZ neuroblasts)
b
MER49 (423-923 nt); NMF program 1 (VZ neuroblasts)
c
MER72 (222-722 nt); NMF program 1 (VZ neuroblasts)
d
ERVL-B4 (4000-4500 nt); NMF program 2 (differentiating granule cells)
e
LTR1A2 (100-600 nt); NMF program 13 (differentiating granule cells)
Attribution score
Δ Attribution score
Δ %Ancestral origin
Accessible - Inaccessible
Coordinates within fragment

Supplementary Fig. 13 | Recurrent usage of ancestral TF motif instances and preservation of ancestral nucleotides in accessible copies. a–e, DeepExplainer attribution profile of the consensus sequence of the TE subfamily (top). Difference in attribution profiles between accessible and inaccessible copies, aligned to the consensus sequence, with the 95% confidence interval shown (middle). Difference in the percentage of nucleotides identical to the consensus sequence between accessible and inaccessible copies, aligned to the consensus sequence (bottom). Plots are shown for MER41B (**a**), MER49 (**b**), MER72 (**c**), ERVL-B4 (**d**), and LTR1A2 (**e**).

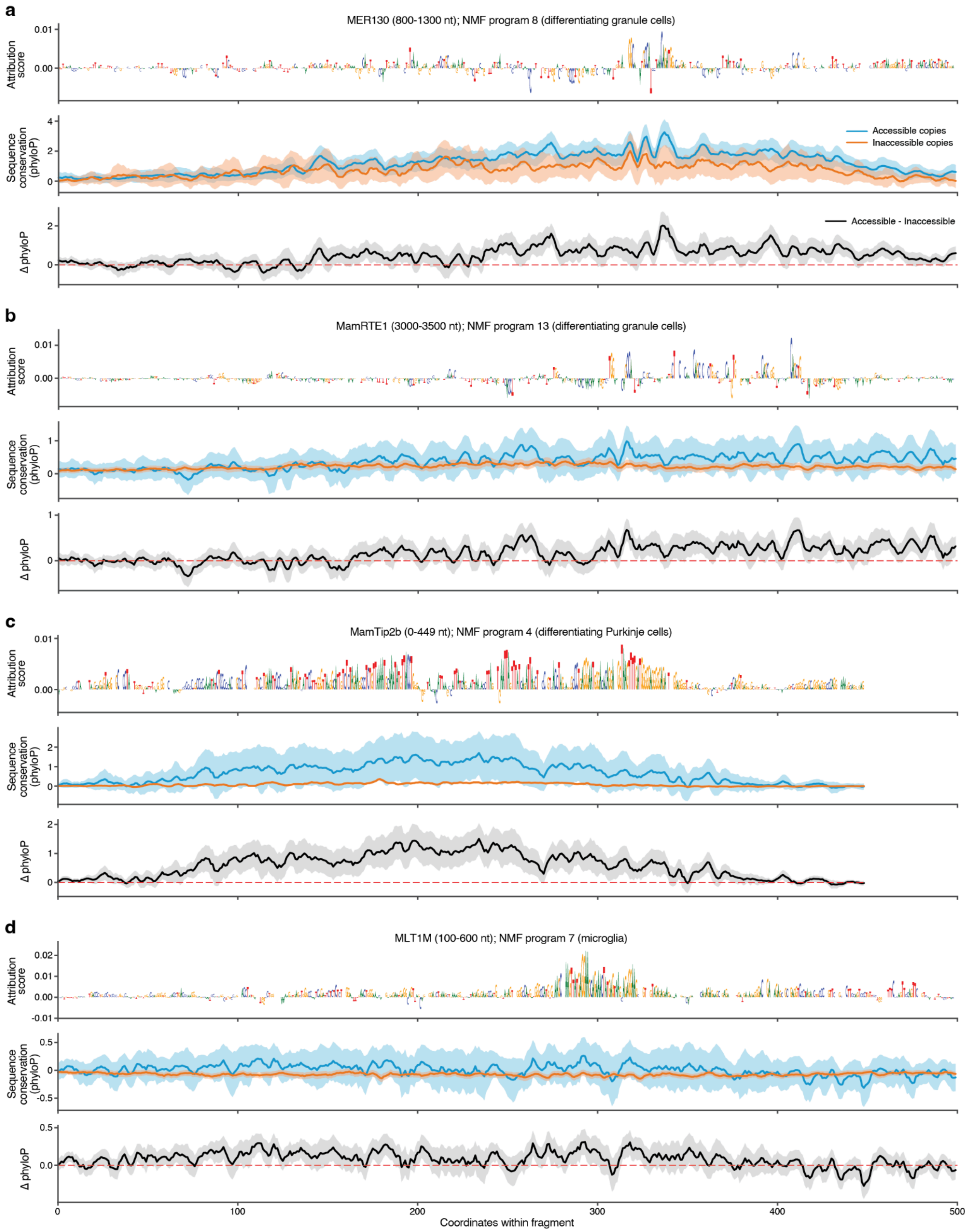

Supplementary Fig. 14 | Cross-mammalian conservation of sequences at ancestral TF binding motif instances in older screened TE subfamilies. a–d, DeepExplainer attribution profile of the consensus

sequence of the TE subfamily (top). Attribution profiles of accessible and inaccessible copies (middle) and the difference between them (bottom), aligned to the consensus sequence, with the 95% confidence interval shown. Plots are shown for MER130 (**a**), MamRTE1(**b**), MamTip2b (**c**), and MLT1M (**d**).

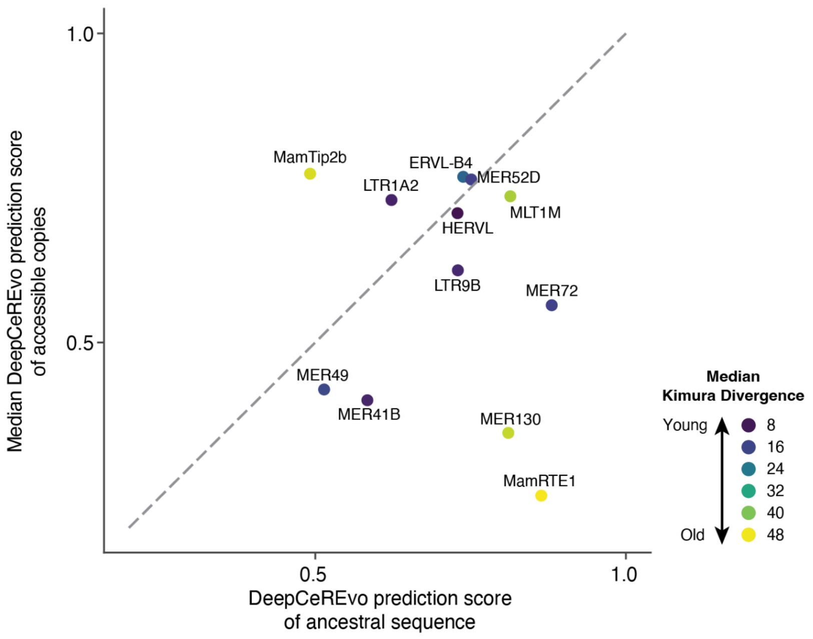

Supplementary Fig. 15 | Comparison of DeepCeREvo prediction scores between extant copies and ancestral sequences. Scatter plot showing the DeepCeREvo score of the ancestral sequence (x-axis) against the median DeepCeREvo score of extant accessible copies (y-axis) for each TE subfamily. The dotted line indicates where ancestral and extant scores are equal; subfamilies falling above it have extant accessible copies with higher median DeepCeREvo scores than their ancestral sequence.

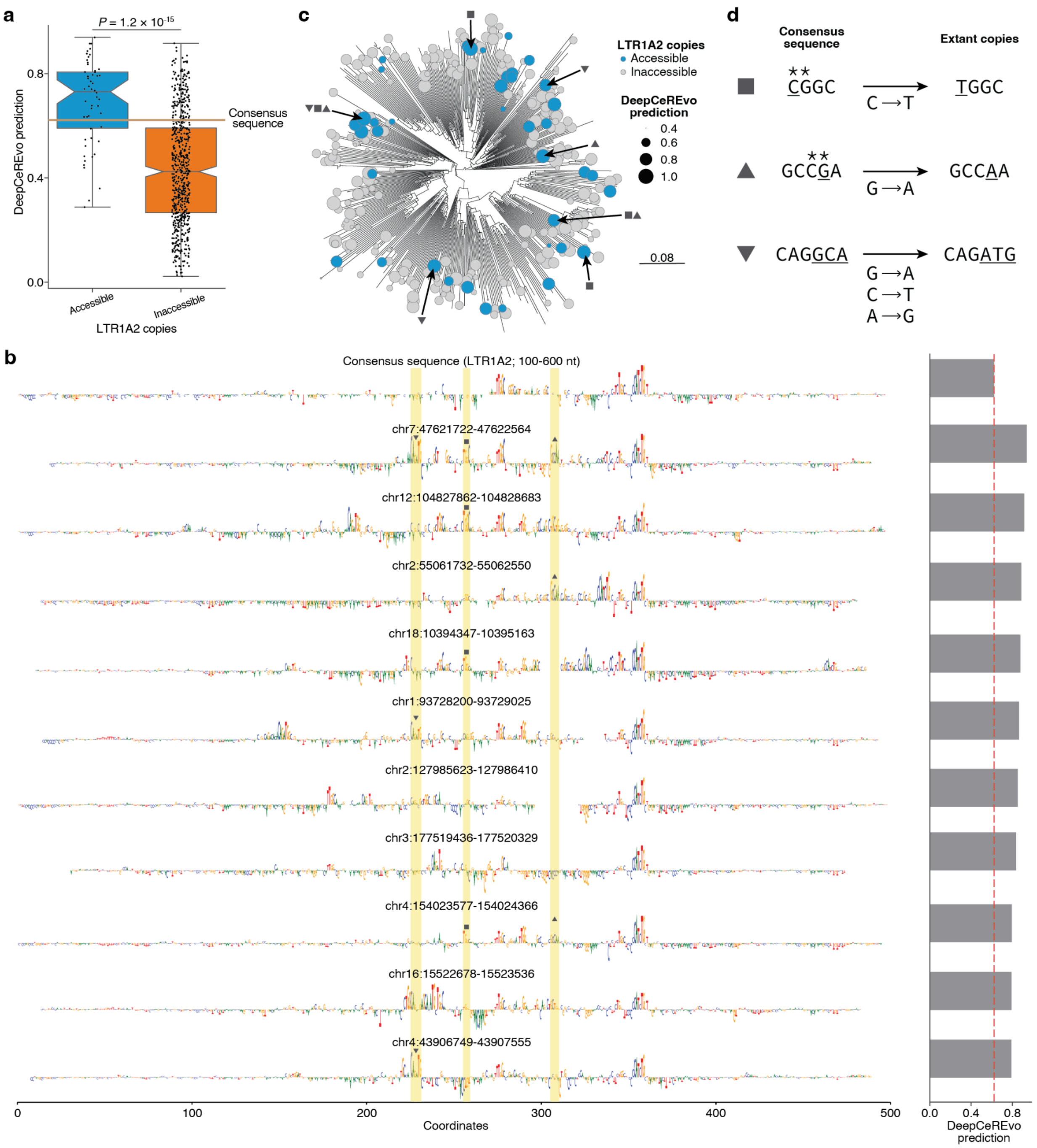

Supplementary Fig. 16 | Convergent acquisition of TF binding motif instances in accessible LTR1A2 copies. **a**, Comparison of DeepCeREvo prediction scores (program 13) between accessible (n = 54) and inaccessible (n = 629) LTR1A2 copies in differentiating granule cells, relative to the score of the consensus sequence. Comparisons were performed using two-sided Mann-Whitney *U* tests. Box plots show the median (centre line), interquartile range (IQR; box bounds), and 1.5× IQR from the box bounds (whiskers). **b**, DeepExplainer attribution profiles (left) and DeepCeREvo prediction scores (right) for the LTR1A2 consensus sequence and the 10 LTR1A2 copies accessible in differentiating granule cells (GC_diff_1) with the highest prediction scores among those that preserved the ancestral TF binding motif

instance. The DeepCeREvo prediction score of the consensus sequence is indicated for reference (dotted red line). TF binding motif instances absent from the consensus sequence but shared across different copies are highlighted and marked. **c**, Phylogenetic relationship of LTR1A2 copies in the human genome, annotated with chromatin accessibility status in differentiating granule cells (GC_diff_1) and corresponding DeepCeREvo prediction scores (program 13). Accessible copies with TF binding motif instances marked in (**a**) are indicated by arrows and their corresponding marks. **d**, For each convergently acquired TF motif instance, the consensus sequence and the corresponding sequences in extant copies are shown, along with the substitution events underlying acquisition of each motif instance. Substituted nucleotides are underlined, and CpG dinucleotides are marked by asterisks. Source data are provided as a Source Data file.

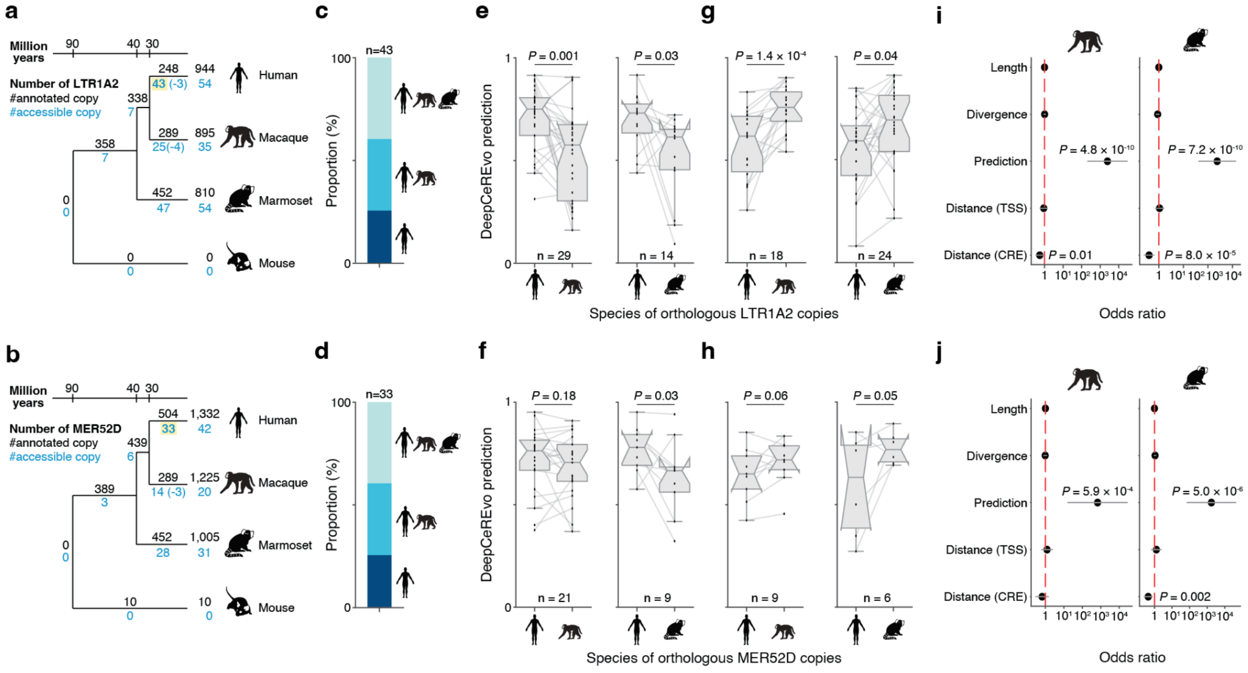

Supplementary Fig. 17 | Species-specific co-option of LTR1A2 and MER52D in differentiating granule cells. a, b, Number of annotated and accessible copies of LTR1A2 (**a**) and MER52D (**b**) in differentiating granule cells (GC_diff_1) across human, macaque, marmoset, and mouse. **c, d,** Insertion age distribution of LTR1A2 (**c**) and MER52D (**d**) copies that are accessible in differentiating granule cells specifically in human. **e, f,** DeepCeREvo prediction scores for differentiating granule cells (program 13) comparing accessible copies of LTR1A2 (**e**) and MER52D (**f**) in human with their corresponding orthologous sequences in macaque (left) and marmoset (right). Comparisons were performed using one-sided Wilcoxon rank-sum tests. **g, h,** DeepCeREvo prediction scores for differentiating granule cells (program 13) comparing accessible copies of LTR1A2 (**g**) and MER52D (**h**) in macaque (left) and marmoset (right) with their corresponding orthologous sequences in human. Comparisons were performed using one-sided Wilcoxon rank-sum tests. **i, j,** Point estimates of odds ratios and 95% confidence intervals for each covariate in logistic regression models predicting accessibility of individual LTR1A2 (**i**) and MER52D (**j**) copies in macaque (left) and marmoset (right). *P*-values for individual covariates were estimated using two-sided Wald tests. No adjustments were made for multiple comparisons. Box plots show the median (centre line), interquartile range (IQR; box bounds), and 1.5× IQR from the box bounds (whiskers). Source data are provided as a Source Data file.

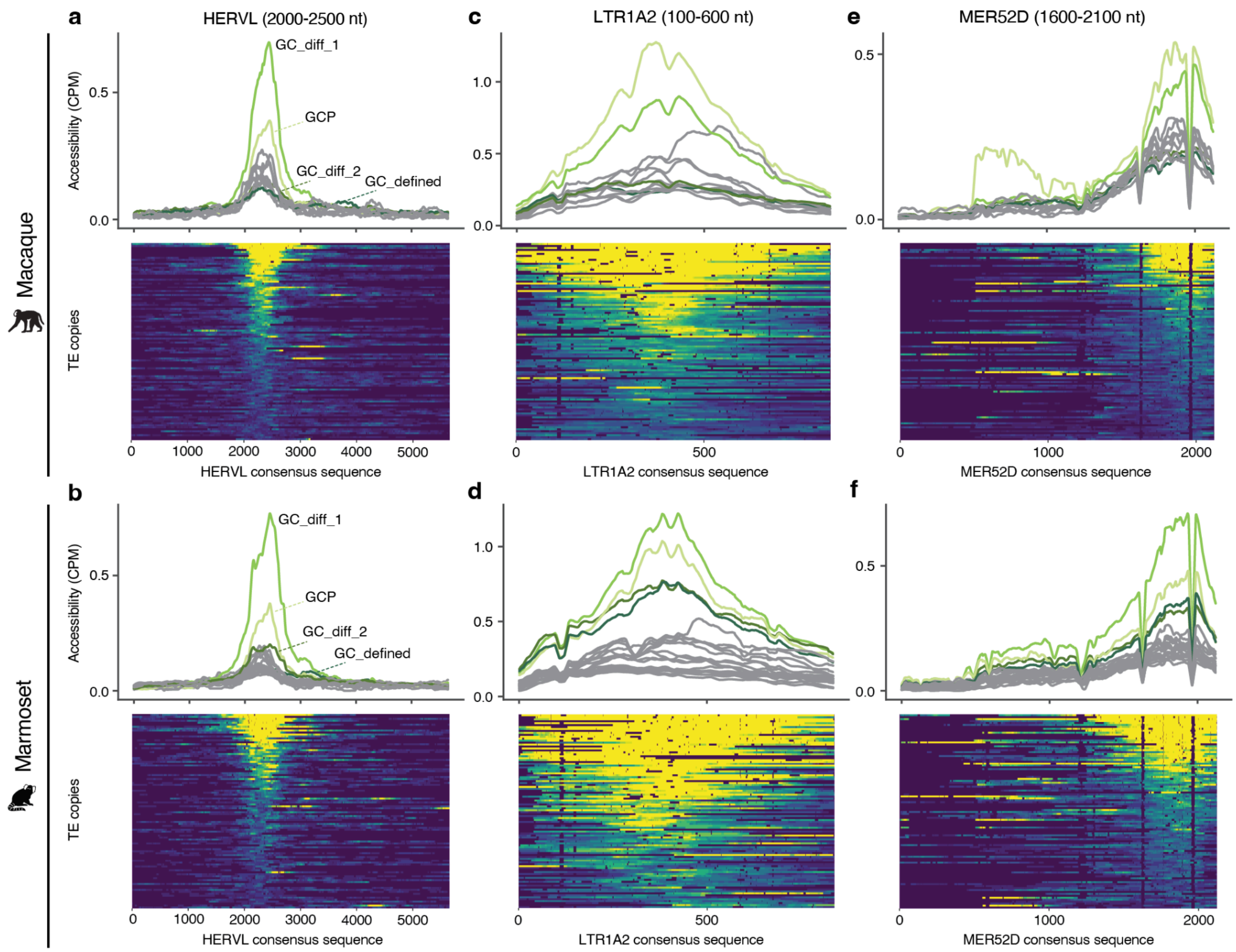

Supplementary Fig. 18 | Accessibility patterns of HERVL, LTR1A2, and MER52D across cerebellar cell types in macaque and marmoset. a–f, Top: Mean chromatin accessibility profiles of the 30 most accessible HERVL (**a, b**), LTR1A2 (**c, d**), and MER52D (**e, f**) copies across cell groups in macaque (**a, c, e**) and marmoset (**b, d, f**) cerebellar development, aligned to the respective consensus sequence. Bottom: Chromatin accessibility patterns of the 100 most accessible HERVL (**a, b**), LTR1A2 (**c, d**), and MER52D (**e, f**) copies in differentiating granule cells (GC_diff_1) in macaque (**a, c, e**) and marmoset (**b, d, f**), aligned to the consensus sequence.

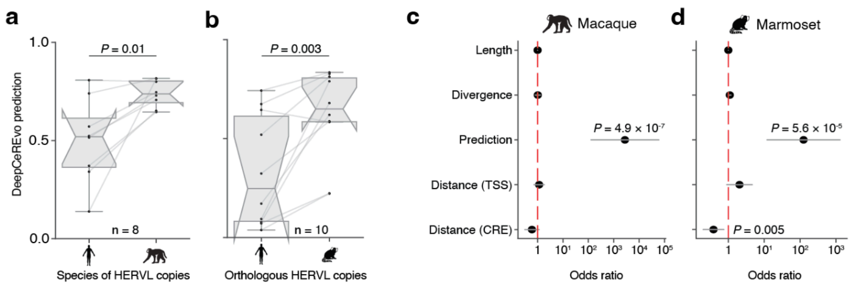

Supplementary Fig. 19 | Species-specific co-option of HERVL in differentiating granule cells. a, b, DeepCeREvo prediction scores for differentiating granule cells (program 13) comparing accessible HERVL copies in macaque (**a**) and marmoset (**b**) with their corresponding orthologous sequences in human. Comparisons were performed using one-sided Wilcoxon rank-sum tests. Box plots show the median (centre line), interquartile range (IQR; box bounds), and 1.5× IQR from the box bounds (whiskers). **c, d,** Point estimates of odds ratios and 95% confidence intervals for each covariate in logistic regression models predicting accessibility of individual HERVL copies in macaque (**c**) and marmoset (**d**). *P*-values for individual covariates were estimated using two-sided Wald tests. No adjustments were made for multiple comparisons. Source data are provided as a Source Data file.

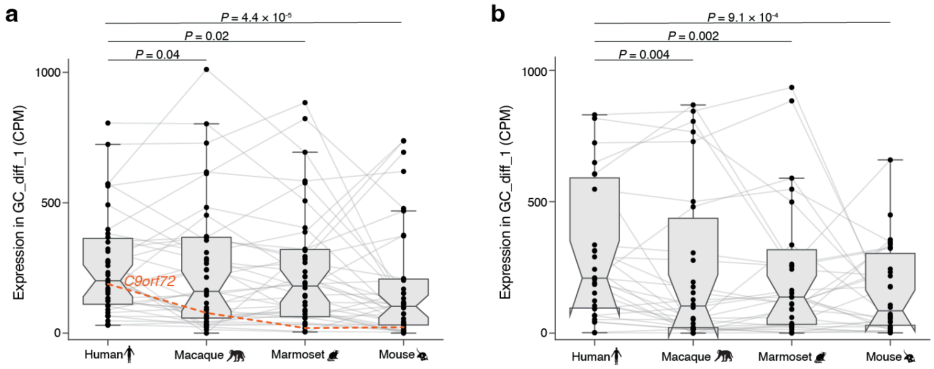

Supplementary Fig. 20 | Human-specific LTR1A2 and MER52D copies contribute to human-specific gene expression. a, b, Expression levels of 1:1 orthologous genes located near LTR1A2 (n = 42) (**a**) and MER52D (n = 30) (**b**) copies that are accessible in differentiating granule cells (GC_diff_1) in human. Comparisons were performed using one-sided Wilcoxon rank-sum tests. Box plots show the median (centre line), interquartile range (IQR; box bounds), and 1.5× IQR from the box bounds (whiskers). Source data are provided as a Source Data file.

Supplementary References

1. Zhang, K. *et al.* A single-cell atlas of chromatin accessibility in the human genome. *Cell* **184**, 5985–6001.e19 (2021).
2. Mannens, C. C. A. *et al.* Chromatin accessibility during human first-trimester neurodevelopment. *Nature* 1–8 (2024).
3. Thompson, C. L. *et al.* A high-resolution spatiotemporal atlas of gene expression of the developing mouse brain. *Neuron* **83**, 309–323 (2014).
