# Supplementary material for: Gene regulatory innovations from transposable elements in primate cerebellum development
Source: Nat Commun. 2026 Jul 30;17:7598. doi: 10.1038/s41467-026-75700-7 (PMC13421694; doi:10.1038/s41467-026-75700-7)
Supplement: Supplementary file 2 — Description of Additional Supplementary Files [file 41467_2026_75700_MOESM2_ESM.docx]

File Name: Supplementary Data 1

Description: Logistic regression analysis of overlaps between transposable element and candidate cis-regulatory elements.

File Name: Supplementary Data 2

Description: Enrichment of candidate cis-regulatory elements derived from transposable elements across cell states (programs) in human cerebellum development.

File Name: Supplementary Data 3

Description: Regulatory potential of 500 bp windows of transposable element subfamilies assessed using DeepCeREvo.

File Name: Supplementary Data 4

Description: Reporter construct sequences and luciferase assay results for selected transposable element fragments.

File Name: Supplementary Data 5

Description: Logistic regression analysis of the accessibility of transposable element copies.

File Name: Supplementary Data 6

Description: Linear regression analysis of sequence conservation in positions with high attribution scores.

File Name: Supplementary Data 7

Description: Expression levels of genes neighboring accessible and inaccessible HERVL copies.
