# Supplementary material for: Gene regulatory innovations from transposable elements in primate cerebellum development
Source: Nat Commun. 2026 Jul 30;17:7598. doi: 10.1038/s41467-026-75700-7 (PMC13421694; doi:10.1038/s41467-026-75700-7)
Supplement: Supplementary file 10 — Reporting Summary [file 41467_2026_75700_MOESM10_ESM.pdf]

nature portfolio

Corresponding author(s): Tetsuya Yamada, Ioannis Sarropoulos, Henrik Kaessmann

Last updated by author(s): Jun 12, 2026

Reporting Summary

Nature Portfolio wishes to improve the reproducibility of the work that we publish. This form provides structure for consistency and transparency in reporting. For further information on Nature Portfolio policies, see our Editorial Policies and the Editorial Policy Checklist.

Statistics

For all statistical analyses, confirm that the following items are present in the figure legend, table legend, main text, or Methods section.

n/a	Confirmed	
☐	☒	The exact sample size (*n*) for each experimental group/condition, given as a discrete number and unit of measurement
☐	☒	A statement on whether measurements were taken from distinct samples or whether the same sample was measured repeatedly
☐	☒	The statistical test(s) used AND whether they are one- or two-sided<br>*Only common tests should be described solely by name; describe more complex techniques in the Methods section.*
☐	☒	A description of all covariates tested
☐	☒	A description of any assumptions or corrections, such as tests of normality and adjustment for multiple comparisons
☐	☒	A full description of the statistical parameters including central tendency (e.g. means) or other basic estimates (e.g. regression coefficient) AND variation (e.g. standard deviation) or associated estimates of uncertainty (e.g. confidence intervals)
☐	☒	For null hypothesis testing, the test statistic (e.g. *F*, *t*, *r*) with confidence intervals, effect sizes, degrees of freedom and *P* value noted<br>*Give P values as exact values whenever suitable.*
☒	☐	For Bayesian analysis, information on the choice of priors and Markov chain Monte Carlo settings
☒	☐	For hierarchical and complex designs, identification of the appropriate level for tests and full reporting of outcomes
☐	☒	Estimates of effect sizes (e.g. Cohen's *d*, Pearson's *r*), indicating how they were calculated

Our web collection on statistics for biologists contains articles on many of the points above.

Software and code

Policy information about availability of computer code

Data collection	No software was used to collect data.
Data analysis	Analyses were performed using bedtools (v2.30.0), MAFFT (v7.505), trimAl (v1.4), IQ-TREE2 (v2.3.4), CrossMap (v0.7.0), pyBigWig (v0.3.18), pybedtools (v0.9.0), famdb.py (v1.0.2), statsmodels (v0.14), and R packages including lme4 (v1.1-36), lmerTest (v3.1-3), pbkrtest (v0.5-0.1), treeio (v1.18.1), and ggtree (v3.2.1).<br>All custom code is available at https://gitlab.com/kaessmannlab/cerebellum_te. An archived snapshot is deposited at Zenodo (10.5281/ZENODO.19354895).

For manuscripts utilizing custom algorithms or software that are central to the research but not yet described in published literature, software must be made available to editors and reviewers. We strongly encourage code deposition in a community repository (e.g. GitHub). See the Nature Portfolio guidelines for submitting code & software for further information.

Data

Policy information about availability of data

All manuscripts must include a data availability statement. This statement should provide the following information, where applicable:

- Accession codes, unique identifiers, or web links for publicly available datasets
- A description of any restrictions on data availability
- For clinical datasets or third party data, please ensure that the statement adheres to our policy

Previously published datasets used in this study are available at Array Express (E-MTAB-9765 and E-MTAB-10533) and the heiData repository (https://doi.org/10.11588/data/QDOC4E and https://doi.org/10.11588/DATA/GDSZG9).

Research involving human participants, their data, or biological material

Policy information about studies with human participants or human data. See also policy information about sex, gender (identity/presentation), and sexual orientation and race, ethnicity and racism.

Reporting on sex and gender	No human data were generated in this study.
Reporting on race, ethnicity, or other socially relevant groupings	No human data were generated in this study.
Population characteristics	No human data were generated in this study.
Recruitment	No human data were generated in this study.
Ethics oversight	No human data were generated in this study.

Note that full information on the approval of the study protocol must also be provided in the manuscript.

Field-specific reporting

Please select the one below that is the best fit for your research. If you are not sure, read the appropriate sections before making your selection.

☒ Life sciences ☐ Behavioural & social sciences ☐ Ecological, evolutionary & environmental sciences

For a reference copy of the document with all sections, see nature.com/documents/nr-reporting-summary-flat.pdf

Life sciences study design

All studies must disclose on these points even when the disclosure is negative.

Sample size	No statistical methods were used to predetermine sample size.
Data exclusions	For reporter assays, three subfamilies were not included: MERVL-2A (high similarity to MERVL), ERVL-E and ERV3-16A3-I (cloning failure in reverse orientation). Wells with firefly luciferase signals <3-fold above background were excluded. All other data were included.
Replication	Data were collected in six independent experiments with three replicates per data point. After quality control, at least two independent experiments per data point remained.
Randomization	Randomization was not applicable. Reporter assays compared sequence variants in a defined set of constructs, where treatment allocation is determined by the experimental design rather than random assignment.
Blinding	Blinding was not relevant. Experiments used automated quantitative measurements (luciferase activity), which are objective and not subject to observer bias.

Reporting for specific materials, systems and methods

We require information from authors about some types of materials, experimental systems and methods used in many studies. Here, indicate whether each material, system or method listed is relevant to your study. If you are not sure if a list item applies to your research, read the appropriate section before selecting a response.

Materials & experimental systems

n/a	Involved in the study
☒	☐ Antibodies
☒	☐ Eukaryotic cell lines
☒	☐ Palaeontology and archaeology
☐	☒ Animals and other organisms
☒	☐ Clinical data
☒	☐ Dual use research of concern
☒	☐ Plants

Methods

n/a	Involved in the study
☒	☐ ChIP-seq
☒	☐ Flow cytometry
☒	☐ MRI-based neuroimaging

Animals and other research organisms

Policy information about studies involving animals; ARRIVE guidelines recommended for reporting animal research, and Sex and Gender in Research

Laboratory animals

P7 RjOrl:SWISS mice (Janvier Labs) were used in this study. The animals were housed under a 12h/12h dark/light cycle in a temperature (20-24 °C) and humidity (40-65%) controlled room with ad libitum access to food and water.

Wild animals

No wild animals were included in this study.

Reporting on sex

Primary granule cell cultures were isolated from postnatal day 7 (P7) mouse pups of both sexes. Sex was not tracked or analyzed as a variable because the experiments measured sequence-specific regulatory activity in vitro, which is not expected to vary by sex.

Field-collected samples

No samples were collected in the field.

Ethics oversight

Animal procedures were performed in compliance with national and international ethical guidelines and regulations, and were approved by the local animal welfare authorities at Heidelberg University Interfaculty Biomedical Research Facility (T-08/24, T-37/24).

Note that full information on the approval of the study protocol must also be provided in the manuscript.

Plants

Seed stocks

Report on the source of all seed stocks or other plant material used. If applicable, state the seed stock centre and catalogue number. If plant specimens were collected from the field, describe the collection location, date and sampling procedures.

Novel plant genotypes

Describe the methods by which all novel plant genotypes were produced. This includes those generated by transgenic approaches, gene editing, chemical/radiation-based mutagenesis and hybridization. For transgenic lines, describe the transformation method, the number of independent lines analyzed and the generation upon which experiments were performed. For gene-edited lines, describe the editor used, the endogenous sequence targeted for editing, the targeting guide RNA sequence (if applicable) and how the editor was applied.

Authentication

Describe any authentication procedures for each seed stock used or novel genotype generated. Describe any experiments used to assess the effect of a mutation and, where applicable, how potential secondary effects (e.g. second site T-DNA insertions, mosiacism, off-target gene editing) were examined.
