# Supplementary material for: Gene regulatory innovations from transposable elements in primate cerebellum development
Source: Nat Commun. 2026 Jul 30;17:7598. doi: 10.1038/s41467-026-75700-7 (PMC13421694; doi:10.1038/s41467-026-75700-7)
Supplement: Supplementary file 11 — Transparent Peer Review file [file 41467_2026_75700_MOESM11_ESM.pdf]

nature portfolio

Peer Review File

Gene regulatory innovations from transposable elements in primate cerebellum development

Corresponding Author: Professor Henrik Kaessmann

This file contains all reviewer reports in order by version, followed by all author rebuttals in order by version.

Version 0:

Reviewer comments:

Reviewer #1

(Remarks to the Author)
In this study Yamada et al. leverage multi-omics, single-cell data on cerebellum to investigate the interplay between cerebellar cell-type-specific cis-regulatory elements (CREs) and Transposable elements (TEs). They identify 17 TE families with ancestral sequences that predispose them to regulatory co-option. Notably, they focus on HERVL, showing its accessibility in differentiating granule cells and its contribution to species-specific expression programs. Along the manuscript, the authors do a good job describing the different steps taken during the study and the questions they are addressing with each analysis. Overall, the study is comprehensive and presents valuable findings and an innovative approach, but the manuscript is hard to read, and the biological conclusions are often hidden behind the results. We believe that the manuscript would benefit from substantial revision and editing. Tighter language and shorted sentences would also improve the manuscript. More detailed critiques are listed below:

• In general, the manuscript is rich in technical detail but could benefit from stronger narrative cohesion. Currently, the results are densely presented with many specialized terms. To reach a broader audience, consider simplifying some of the jargon and adding brief contextual explanations, e.g., how these TE-driven regulatory patterns compare to known principles of enhancer evolution or cell-type-specific chromatin remodeling.

• The manuscript includes specialized terms (e.g., Kimura divergence, NMF analysis, co-option potential) that may not be familiar to all readers, since Nature Communication reaches a broader audience. Briefly defining such terms in the Results section would improve clarity to a broader audience.

• We are not familiar with non-negative matrix factorization (NMF) analysis, but it appears that the numerical program identifiers may be arbitrary. Could the authors order them to correspond to developmental progression (e.g., smaller numbers = earlier stages)? This would make trends in Figures 1d and 1f easier to interpret visually.
Additionally, since the NMF programs are central to the study, I suggest including a main-text table summarizing each program's associated cell type and developmental stage. Figure 1c contains this information, but the color-coded labels are difficult to trace, and several colors are too similar—especially between developmental stages. Consider adjusting the palette (e.g., darker shades for later stages) to improve readability.

• The authors appear to be using HERVL and ERVL interchangeably, which is confusing since HERVL refers specifically to the human version of the ERVL family. This should be clarified throughout.

• In the section describing enhancer assays, the rationale for using mouse cells to test a human TE should be better justified, as using human or primate cells might provide a more relevant functional context.

• When referring to the "17 TE fragments," please clarify whether these represent individual insertions or TE families/subfamilies. Based on Figure 2, it seems they refer to TE families; if so, "family" or "subfamily" would be more accurate than "fragment." Also, while HERVL is described in very high level of detail, the other 16 TE families are not sufficiently discussed. Even a brief description of their biological relevance or cell-type-specific patterns would help readers appreciate the broader landscape of TE co-option.

• The discussion is short and doesn't really add much to the interpretation of the results.

(Remarks on code availability)

Reviewer #2

(Remarks to the Author)
The manuscript by Yamada et al. examines the contribution of transposable elements (TEs) to the gene regulatory evolution coordinated by cis-regulatory elements (CREs) within the mammalian cerebellum. These findings add to a growing body of cross-species single-cell multiomics studies evaluating TE regulatory activity (e.g., (Simon et al., Li et al., Zemke et al.). The work substantially leverages the authors' previous work with the DeppCeREvo model. The HERVL case study is the main empirical novelty. As written, the manuscript is quite well written and my concerns are only modest ones on two issues limit generality: (i) enrichment framing without full treatment of caveats, and (ii) heavy emphasis on HERVL relative to the stated aim of generalizable TE principles.
Major Points:

1) The central patterns rely on overlap/enrichment. Please foreground limitations and add controls. In particular, while it is not uncommon in the field to present simple depletion of enrichment as, itself, a signal (and this is quite plausible biologically), it really isn't a great practice from a statistical or even logical perspective. Perhaps that can be handled textually but there are some improved benchmarking or controls that could be done throughout. E.g., Define background for each comparison and report multiple backgrounds if possible with controls where appropriate (e.g., length matched, distance-to-TSS-matched, etc). Consider replacing some enrichment with regression where possible. Some of the multiple testing control is not clear enough.
2) As initially described, this is a general paper, but the narrative is unusually focused on a specific use case. That use case (HERVL) is interesting and well described but not prominent or well grounded enough to be a paper. I think the manuscript should probably be expanded beyond a single case study. There are other TE fragments suggested in the figures and the same minimal functional validation would be valuable for, perhaps, two more.
Minor:
The method is included somewhat by reference in this manuscript. Probably spelling out aspects of the training and evaluation needs to be more clearly explained.
Proximity of HERVL copies needs a permutation matched by distance control.
Missing confidence intervals on some of the reporting

(Remarks on code availability)

Reviewer #3

(Remarks to the Author)
Yamada et al. conducted a comprehensive analysis of the relationship between transposable elements (TEs) and cis-regulatory elements (CREs) during cerebellar development, focusing primarily on primates and including comparative analyses in mouse. Their study systematically examined how TEs contribute to the acquisition of cell-lineage-specific CREs and to regulatory innovation during human evolution.
In particular, they demonstrated that distinct sequence motifs within HERVL elements contribute to the emergence of transcription-factor (TF) binding sites in different cell lineages. The TF-binding motifs identified in the 2,000–2,500 nt region of HERVL are derived from proto-motifs present in ancestral sequences and were functionally reinforced through human-specific substitutions that enhance enhancer activity. The authors further showed that the regulatory activity of HERVL-derived CREs depends not only on the presence of TF-binding motifs but also on the density of neighboring non-TE-derived CREs, highlighting the role of local chromatin context in enabling HERVL activation. Finally, they demonstrated that the sets of active HERVL copies and their target genes differ across species, suggesting that HERVL insertions have contributed to the evolution of species- and cell-type-specific gene-expression programs in primates.
This study provides important insights into how TEs have been co-opted as cell-type-specific CREs and how these events have influenced gene-expression patterns in the primate cerebellum. It represents a significant contribution to our understanding of the interplay between TEs, genome evolution, and species diversification.
Overall, the manuscript is clearly written and presents a coherent and well-supported set of analyses. Addressing the points raised below would further refine the work and enhance its impact.

Major comments 1
The section "TE contributions to species- and cell-type-specific cis-regulatory elements in mammalian cerebellum development" compellingly shows that TE-derived elements are enriched among species-specific cCREs, with particularly strong contributions in primates (~60%) compared to mouse (~34%). This supports the general idea that TEs contribute to lineage-specific regulatory innovation. However, it remains unclear whether the authors interpret this as a broadly mammalian trend or as a phenomenon primarily driven by primate evolution. In primates, TE-derived cCREs are clearly enriched relative to the genomic TE background, whereas in mouse this proportion may be lower or even below the genomic expectation.
Moreover, the evidence for species-specific TE contributions in mouse appears comparatively limited throughout the study—largely serving as a reference or phylogenetic comparison rather than providing parallel mechanistic insights. If TE-driven

co-option in mouse is indeed weaker or not statistically enriched, the current framing and title ("mammalian cerebellum development") might slightly overstate the generality of the findings. Conversely, if the lower proportion in mouse reflects faster TE sequence decay or longer divergence time rather than a genuine biological difference, clarifying this point would substantially strengthen the interpretation.
I would encourage the authors to explicitly report TE enrichment values relative to genomic coverage in each species and to clarify whether the conclusions are intended to describe mammalian-wide mechanisms or primarily primate-specific innovations. Depending on this clarification, the title might be adjusted to more accurately reflect the scope of the presented evidence.

Major comments 2
In Figure 1f, several TE subfamilies—notably from the ERV1 class but also including certain LINE, SINE, and DNA transposon families—show enrichment across multiple NMF programs. This pattern appears to contrast with the notion that cell-type-specific cCREs are more frequently TE-derived. It would be helpful if the authors could clarify whether these shared enrichments reflect (i) recurrent co-option of distinct copies from the same TE subfamily in different cell types, (ii) the presence of broadly active promoter-like motifs within TEs, or (iii) background accessibility biases associated with ancient TE insertions. A short discussion of these possibilities would help reconcile the apparent overlap of TE enrichment across programs with the conclusion of cell-type-specific TE co-option.

Major comments 3
The authors conclude that pre-existing cis-regulatory motifs in ancestral TE sequences facilitate their preferential co-option as CREs. This interpretation is compelling, but it assumes that the relevant TF motifs were already present in ancestral states. However, convergent emergence of TF-binding motifs after insertion has also been documented for several TE families. It would therefore be informative to test whether ancestral TE fragments that already contained motifs show stronger cCRE enrichment than motif-lacking fragments, to distinguish between pre-adaptive and convergent modes of regulatory evolution.

Major comments 4
The authors propose that pre-existing TF binding motifs in ancestral TE sequences facilitate their co-option as cCREs. However, Figure 1g and related analyses show that older LINE and SINE subfamilies exhibit stronger enrichment in cCREs than younger ones. This pattern may be more consistent with post-insertion sequence evolution, whereby mutations accumulated over time created novel TF binding sites and regulatory potential, rather than preservation of ancestral motifs. It would be helpful if the authors could clarify whether such classes represent cases of motif emergence through sequence drift, as opposed to ancestral motif retention, and how this distinction fits within their overall model of TE-driven regulatory innovation.

Major comments 5
The attribution analysis in Fig. 2 suggests that specific TF binding motifs within ancestral TE fragments contribute to their predicted regulatory potential. However, attribution maps indicate sequence importance in silico and do not necessarily imply biological relevance. To support the conclusion that these motifs facilitate co-option, it would be valuable to assess whether the predicted motif sequences are evolutionarily conserved — either across genomic TE copies or between species. Demonstrating significant conservation of these motifs would strengthen the argument that they are functionally constrained and relevant for TE-derived CRE activity.

Major comments 6
In Figure 5, the authors conclude that HERVL accessibility depends not only on motif preservation but also on the local chromatin context, such as the density of nearby cCREs. While this is biologically plausible, it raises an important conceptual issue regarding the interpretation of DeepCeREvo prediction scores. If accessibility is largely determined by the surrounding regulatory landscape, then high DeepCeREvo scores for non-accessible HERVL copies might reflect an overestimation of intrinsic enhancer potential.
Clarifying whether DeepCeREvo captures purely sequence-intrinsic potential or integrates features that indirectly correlate with genomic context would help readers interpret the discrepancy between predicted and observed accessibility.

Major comments 7
Figure 6 suggests that species-specific accessible HERVL elements contribute to divergence in gene expression. However, given that accessibility appears to depend on insertion into pre-existing active chromatin regions enriched for non-TE cCREs (Fig. 5), it remains possible that these genomic neighborhoods were already primed for high activity before HERVL integration. In that case, HERVL insertions may have amplified rather than initiated species-specific expression programs. Clarifying whether HERVL served as a causal driver of regulatory divergence or acted as an enhancer of pre-existing regulatory trajectories would strengthen the interpretation.

Minor comment:
In Fig.1, the definition of TE-derived cCREs as requiring ≥50% (≥250 bp) overlap with a TE annotation seems quite conservative. Because many TE co-option events involve only partial fragments or individual motif remnants, this threshold might underestimate the true extent of TE-derived regulatory sequences. Please clarify the rationale for this choice, or comment on whether more relaxed thresholds (e.g., ≥50–100 bp) yield consistent trends.

In Figure 2b, the use of a whole-body chromatin accessibility atlas as background is conceptually sound. However, because TE enrichment can be influenced by genomic context and sequence composition, the analysis would benefit from additional

controls matched for GC content, CpG density, peak length, mappability, conservation, and regulatory context (e.g., promoter versus distal). Stratified or nearest-neighbor matching, or complementary permutation-based background models (e.g., circular chromosome randomization), would provide a more rigorous assessment that the observed positional enrichments reflect biologically specific co-option rather than underlying genomic biases.

(This point is related to Major comment 5.)
The authors conclude that HERVL-specific substitutions generated TF-binding motifs underlying enhancer activity. This is a compelling model; however, the evidence would be considerably strengthened by showing conservation of these motif sites across genomic HERVL-derived cCRE copies and among different primate species. A multiple sequence alignment or conservation plot demonstrating that the key motif nucleotides are consistently preserved among active copies would provide direct support for the functional relevance of these substitutions.

Recent work by Sekine et al. (Communications Biology, 2023) has also demonstrated that transposable elements shape cell-type-specific open chromatin landscapes in the mouse brain. Citing this study would help appropriately contextualize the novelty of the present findings within the broader literature on TE-mediated regulatory evolution.

Figure panel cross-references appear to be mismatched in several places. Please verify and correct the following:
• Lines 254–255: “Fig. 3a” ↔ “Fig. 3b” appear swapped
o l254: change Fig. 3a → Fig. 3b
o l255: change Fig. 3b → Fig. 3a
• Lines 298–301: References to Fig. 3e,f seem off
o l298: change Fig. 3e → Fig. 3g
o l301: change Fig. 3e,f → Fig. 3g,h
• Lines 320–324: Fig. 4a/b appear reversed
o l320: change Fig. 4a → Fig. 4b
o l324: change Fig. 4b → Fig. 4a

(Remarks on code availability)

Reviewer #4

(Remarks to the Author)
I co-reviewed this manuscript with one of the reviewers who provided the listed reports. This is part of the Nature Communications initiative to facilitate training in peer review and to provide appropriate recognition for Early Career Researchers who co-review manuscripts.

(Remarks on code availability)

Version 1:

Reviewer comments:

Reviewer #1

(Remarks to the Author)
The authors have addressed all our critique and the manuscript has been substantially revised. We are happy with the changes.

(Remarks on code availability)

Reviewer #2

(Remarks to the Author)
I thank the authors for a comprehensively satisfying set of revisions in answer to my originally raised questions.

(Remarks on code availability)

Reviewer #3

(Remarks to the Author)
The authors have responded carefully and convincingly to all of my comments. The revised manuscript is substantially improved by additional analyses that clarify the scope of the study, extend the findings beyond the original HERVL-focused case study, and strengthen the statistical support for the major conclusions. In particular, the analyses of additional TE subfamilies now better distinguish different modes of TE co-option, including ancestral regulatory potential and convergent

acquisition of new motif instances. I also appreciate the more nuanced interpretation that TE-derived elements may amplify pre-existing regulatory programs rather than simply create new regulatory circuits.

Overall, I am satisfied with the revision and recommend publication. I have only a few minor suggestions:
1. The LTR1A2 convergent evolution finding (Supp Fig. 16) is one of the most interesting additions—consider giving it slightly more prominence in the Discussion as it elegantly demonstrates that "TE co-option" is not a monolithic phenomenon.
2. I noticed that the colors in the TE family legend in Fig. 1g may not fully match the colors shown in the corresponding plot, although this could be a PDF conversion issue. I suggest that the authors check the final figure carefully during production.

(Remarks on code availability)

Reviewer #4

(Remarks to the Author)
I co-reviewed this manuscript with one of the reviewers who provided the listed reports. This is part of the Nature Communications initiative to facilitate training in peer review and to provide appropriate recognition for Early Career Researchers who co-review manuscripts.

(Remarks on code availability)

Open Access This Peer Review File is licensed under a Creative Commons Attribution 4.0 International License, which permits use, sharing, adaptation, distribution and reproduction in any medium or format, as long as you give appropriate credit to the original author(s) and the source, provide a link to the Creative Commons license, and indicate if changes were made.
In cases where reviewers are anonymous, credit should be given to 'Anonymous Referee' and the source.
The images or other third party material in this Peer Review File are included in the article's Creative Commons license, unless indicated otherwise in a credit line to the material. If material is not included in the article's Creative Commons license and your intended use is not permitted by statutory regulation or exceeds the permitted use, you will need to obtain permission directly from the copyright holder.
To view a copy of this license, visit https://creativecommons.org/licenses/by/4.0/

Response to the referees

We thank the referees for thoroughly reviewing our manuscript and for their positive feedback and constructive criticism. We have made every effort to fully address each of their concerns, which we believe has resulted in a substantially improved manuscript.

Below we provide a summary of the major changes to the manuscript, followed by detailed point-by-point responses to each referee's comments. To facilitate readability, we use various font emphases and colors, as outlined below:

The comments from the referees are in black;
our responses are in blue;
text cited from the manuscript is in italics, *with specific changes highlighted by underline*

Page and line numbers refer to the current revised manuscript. Figures exclusively addressed to the reviewers are termed "Reviewer Figures".

The key revisions are summarized as follows:

- We have extended our detailed characterization of HERVL to additional TE subfamilies identified as preferentially co-opted for regulatory activity, including the characterization of their accessibility profiles in the developing human brain, the determinants of accessibility of their individual copies, and their contributions to gene regulatory divergence between primate species.
- We have performed rigorous statistical tests based on multivariate logistic and linear regression to strengthen support for (1) the enrichment of TE-derived sequences in cCREs in less-constrained genomic contexts; (2) the determinants of copy-level TE accessibility; and (3) sequence preservation at transcription factor binding motif instances derived from ancestral elements.
- We have revised the manuscript to improve accessibility and interpretability for a broader audience. Specifically, we have added (1) broader motivation for each analysis at the beginning of each Results section, (2) more thorough interpretation of each analysis within the Results, and (3) an expanded Discussion section with more generalizable conclusions.

We believe these additions support our claims in the original manuscript, and substantially strengthen its generality.

Contents

Reviewer: 1 .... **3**
COMMENTS 3
Reviewer: 2 .... **18**
MAJOR POINTS 18
MINOR POINTS 31
Reviewer: 3 .... **35**
MAJOR COMMENTS 35
MINOR COMMENTS 56
Reviewer: 4 .... **60**

Reviewer: 1

In this study Yamada et al. leverage multi-omics, single-cell data on cerebellum to investigate the interplay between cerebellar cell-type-specific cis-regulatory elements (CREs) and Transposable elements (TEs). They identify 17 TE families with ancestral sequences that predispose them to regulatory co-option. Notably, they focus on HERVL, showing its accessibility in differentiating granule cells and its contribution to species-specific expression programs. Along the manuscript, the authors do a good job describing the different steps taken during the study and the questions they are addressing with each analysis. Overall, the study is comprehensive and presents valuable findings and an innovative approach, but the manuscript is hard to read, and the biological conclusions are often hidden behind the results. We believe that the manuscript would benefit from substantial revision and editing. Tighter language and shorted sentences would also improve the manuscript. More detailed critiques are listed below:

We thank the reviewer for the thoughtful summary of our work and the constructive feedback. We have substantially revised the manuscript to improve readability, tighten the language, and ensure the biological conclusions are more clearly foregrounded throughout.

COMMENTS

- In general, the manuscript is rich in technical detail but could benefit from stronger narrative cohesion. Currently, the results are densely presented with many specialized terms. To reach a broader audience, consider simplifying some of the jargon and adding brief contextual explanations, e.g., how these TE-driven regulatory patterns compare to known principles of enhancer evolution or cell-type-specific chromatin remodeling.

We have revised the manuscript to improve narrative flow and accessibility. Specifically,

- We have added background information at the beginning of each Results section to clarify the current consensus in the field and to contextualise the analyses presented therein:

  [Page 6, Line 190] *The preferential co-option of certain TE subfamilies into cell-type-specific cCREs can be driven by pre-existing sequence features capable of functioning as regulatory elements, such as TF binding sites, as demonstrated in several TE subfamilies in specific tissues using reporter assays[17,19,20]. However, systematically assessing the cis-regulatory potential—the propensity to be co-opted as CREs based on pre-existing sequence features in their ancestral form—of diverse TE subfamilies across cell types requires more scalable approaches. To address this, we leveraged recent advances in sequence-to-function models predicting cCRE accessibility from DNA sequence[56–59].*

  [Page 7, Line 234] *The vast diversity of neural cell types is generated from a small pool of progenitors through spatiotemporal patterning cues and their downstream transcriptional programs[69,70]. Cell types originating from the same anatomical region or developmental window therefore share portions of their transcriptional programs, driven by common*

TFs[60,71,72]. Consistently, the screened TE subfamilies showed specific accessibility in their respective cell lineages.

[Page 9, Line 307] *Long terminal repeats (LTRs) of endogenous retroviruses function as transcriptional regulatory elements for retroviral genes and harbor a high density of TF binding sites, making them a major source of TE-derived regulatory elements[45,46]. Consistent with this, the majority of TE subfamilies identified in our screening (7 of 12) are LTR elements. The internal protein-coding regions of endogenous retroviruses, by contrast, have received less attention as potential sources of co-opted regulatory elements, given their original protein-coding function, yet our screening identified two such cases: HERVL and ERVL-B4, whose internal sequences show highly specific accessibility in differentiating granule cells (Fig. 2e).*

[Page 10, Line 353] *Although the screened TE subfamilies harbor regulatory potential in their ancestral sequences, individual genomic copies vary in whether they become co-opted as regulatory elements. For instance, despite having high regulatory potential in their ancestral state, only 2.1% of HERVL copies are accessible in differentiating granule cells, raising the question of what distinguishes accessible from inaccessible copies.*

[Page 12, Line 435] *TEs with intrinsic regulatory potential provide a substrate for repeated, independent co-option events, raising the possibility that the same TE subfamily is deployed differently across species to drive lineage-specific gene expression. To test whether this occurs during primate brain development, we analyzed …*

- We have replaced technical jargon with simpler, more intuitive terms in the main text. Specifically:

  1. We have replaced "NMF program" with "program" throughout the manuscript. This terminology is also consistent with the original publication that introduced these 18 chromatin accessibility programs in the developing cerebellum (Sarropoulos et al., *Science*, 2026 [PMID: 41610256])
  2. We have replaced "Kimura divergence" with "divergence of (individual genomic) copies from the consensus sequence."

While these changes do not affect technical precision, detailed explanations of both terms are provided in the Methods section. Accordingly, we have modified the Results sections, and the sentences introducing these terms at first appearance now read as follows:

[Page 4, Line 126] *To characterize cell-state-specific patterns of TE integration into cCREs, we analyzed cCREs associated with 18 programs capturing cell-state- and time-specific chromatin accessibility patterns based on dimensionality reduction by non-negative matrix factorization[39] (Table 1; Methods).*

[Page 5, Line 177] *TE subfamily ages were inferred from the median divergence of individual genomic copies from their consensus sequence, with higher divergence reflecting older insertions[55] (Methods).*

- We have added discussion to provide a stronger narrative thread linking our results. Specifically, we included a broader interpretation of our findings on TE-driven regulatory evolution in comparison to non-TE-driven regulatory evolution. We have added the following text to the Discussion section:

  [Page 14, Line 523] *In addition to these preferential co-option events of specific TE subfamilies, our results suggest that evolutionary constraints have a broad impact on shaping the overall distribution of TE-derived cCREs across cell types. This conclusion is supported by consistent patterns observed in our study and others showing that TE-derived cCREs are enriched in less-constrained genomic contexts: distal elements rather than promoters or exonic elements, species-specific rather than conserved regions, cell-type-specific rather than broadly accessible elements[23], and elements active in later rather than earlier developmental stages[61]. These patterns are consistent with evolutionary processes—mutation, natural selection, and genetic drift—where inserted TE copies serve as raw material for regulatory evolution similarly to other non-TE-derived sequences, rather than providing a "shortcut" via pre-adaptive sequences in their ancestral state. This model is further supported by older TE subfamilies from LINE, SINE, and DNA classes showing higher cCRE enrichment than younger ones, while the absence of this age dependence in TE subfamilies from LTR and ERV-int classes points instead to preferential co-option via pre-existing regulatory sequences. Together, these observations highlight that understanding TE contributions to regulatory evolution requires considering both evolutionary constraint and preferential co-option driven by ancestral sequence features.*

We believe these revisions strengthen the readability of the manuscript and make our findings more accessible to a broader audience.

- The manuscript includes specialized terms (e.g., Kimura divergence, NMF analysis, co-option potential) that may not be familiar to all readers, since Nature Communication reaches a broader audience. Briefly defining such terms in the Results section would improve clarity to a broader audience.

As described in our response to the previous comment, "Kimura divergence" and "NMF analysis" no longer appear in the Results section. Nevertheless, we have added simple, intuitive explanations of these concepts at their first appearance, alongside a definition of "co-option potential." Specifically, we have added text as follows:

- Kimura divergence:
  [Page 5, Line 177] *TE subfamily ages were inferred from the median divergence of individual genomic copies from their consensus sequence, with higher divergence reflecting older insertions[55] (Methods).*

- NMF analysis:
  [Page 4, Line 126] *To characterize cell-state-specific patterns of TE integration into cCREs, we analyzed cCREs associated with 18 programs capturing cell-state- and time-specific*

chromatin accessibility patterns based on dimensionality reduction by non-negative matrix factorization[39] *(Table 1; Methods).*

- Co-option potential (regulatory potential):
  [Page 6, Line 193] *However, systematically assessing the cis-regulatory potential—the propensity to be co-opted as CREs based on pre-existing sequence features in their ancestral form—of diverse TE subfamilies across cell types requires more scalable approaches.*

We believe these modifications improve clarity for a broader audience.

- We are not familiar with non-negative matrix factorization (NMF) analysis, but it appears that the numerical program identifiers may be arbitrary. Could the authors order them to correspond to developmental progression (e.g., smaller numbers = earlier stages)? This would make trends in Figures 1d and 1f easier to interpret visually.

While we recognize that developmental ordering would provide an intuitive visual framework, the program identifiers follow the numbering established in the original reference (Sarropoulos et al., *Science*, 2026 [PMID: 41610256]), and reordering them would create inconsistency with the published resource, potentially causing confusion for readers comparing across studies. To address the concern about visual interpretability, we have clarified the existing visual annotations in the figure legend and added differentiation trajectories within each cell lineage to further improve interpretability (Fig. 1e). This approach preserves consistency with the published data while improving the accessibility of our figures.

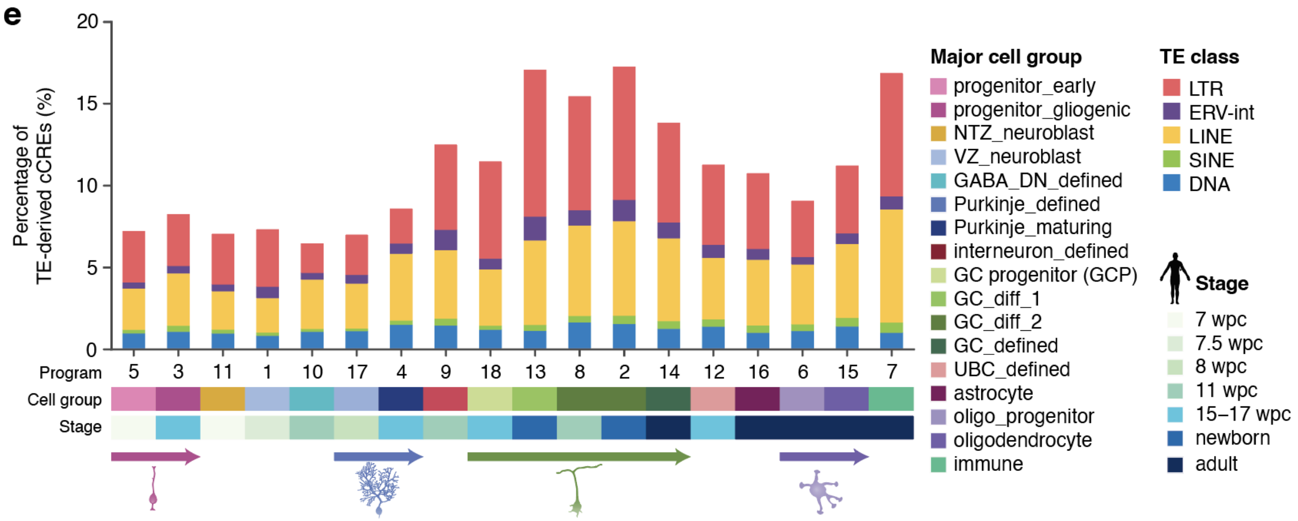

***Fig. 1e** | Percentage of program-specific cCREs that originate from TEs across human cerebellar cell types and developmental stages. For each program, the cell group and developmental stage of highest activity are indicated, along with differentiation trajectories within each cell lineage.*

We have also added the following clarification to the Methods section:

> **[Page 16, Line 572]** *The number of programs was determined to be 18 based on the reconstruction error and the distance between cCREs from human and mouse in the same group. The order of the programs follows that of the original publication to maintain consistency between publications.*

- Additionally, since the NMF programs are central to the study, I suggest including a main-text table summarizing each program's associated cell type and developmental stage. Figure 1c contains this information, but the color-coded labels are difficult to trace, and several colors are too similar—especially between developmental stages. Consider adjusting the palette (e.g., darker shades for later stages) to improve readability.

We have retained the color palette from the original study (Sarropoulos et al., *Science*, 2026 [PMID: 41610256]) to maintain consistency and allow direct comparison across publications. With the addition of Table 1, which explicitly lists each program's associated cell type and developmental stage, the figure should be unambiguous without relying on color alone.

Program	Major cell group	Contributing cell groups, unabbreviated (Developmental stage)
1	VZ_neuroblast	Ventricular zone neuroblast (CS18–19; CS20; CS22–23)
2	GC_diff_2	Differentiating granule cell 2 (newborn; infant); Defined granule cell (infant)
3	progenitor_gliogenic	Early progenitor (11 wpc); Bipotent progenitor (11 wpc; 15–17 wpc); Gliogenic progenitor (11 wpc; 15–17 wpc)
4	Purkinje_maturing	Defined Purkinje cell (11 wpc); Maturing Purkinje cell (15–17 wpc)
5	progenitor_early	Early progenitor (CS18–19; CS20; CS22–23)
6	oligo_progenitor	Oligodendrocyte progenitor cell (15–17 wpc; adult)
7	immune	Immune cell/microglia (adult)
8	GC_diff_2	Differentiating granule cell 1 (15–17 wpc); Differentiating granule cell 2 (11 wpc; 15–17 wpc)
9	interneuron_defined	Differentiating interneuron (11 wpc; 15–17 wpc); Defined interneuron (15–17 wpc)
10	GABA_DN_defined	Defined GABAergic deep nuclei neuron (CS20; CS22–23; 11 wpc)
11	NTZ_neuroblast	Differentiating isthimic neuron (CS18–19); Nuclear transitory zone neuroblast (CS18–19; CS20); Defined glutamatergic deep nuclei neuron (CS22–23)
12	UBC_defined	Differentiating unipolar brush cell (15–17 wpc); Defined unipolar brush cell (15–17 wpc)
13	GC_diff_1	Granule cell progenitor (newborn); Differentiating granule cell (newborn; infant)
14	GC_defined	Defined granule cell (infant; adult)

15	oligodendrocyte	Oligodendrocyte (adult)
16	astrocyte	Astrocyte (adult)
17	Purkinje_defined	Differentiating Purkinje cell (CS20; CS22–23); Defined Purkinje cell (CS22–23)
18	GCP	Granule cell progenitor (11 wpc; 15–17 wpc)

Table 1 | Programs of chromatin accessibility in the developing cerebellum. *Annotation of 18 chromatin accessibility programs across cell types and developmental stages in the developing cerebellum, as identified by non-negative matrix factorization in Sarropoulos et al., 2026[39]. CS, Carnegie stage; wpc, weeks post conception.*

- The authors appear to be using HERVL and ERVL interchangeably, which is confusing since HERVL refers specifically to the human version of the ERVL family. This should be clarified throughout.

We thank the reviewer for raising this point. ERVL refers to a family of endogenous retroviruses, while HERVL is a specific subfamily within this family. Although we note early in the Results that "TEs are classified hierarchically into classes, families, and subfamilies based on replication mechanisms and sequence features," we recognize this alone may not have been sufficient to prevent confusion, particularly given the similarity between the names ERVL and HERVL.

To improve clarity, we have replaced "ERVL family transposons" with "TE subfamilies closely related to HERVL within the ERVL family." This revision maintains technical precision while avoiding confusion arising from the hierarchical naming conventions of TE classification.

- In the section describing enhancer assays, the rationale for using mouse cells to test a human TE should be better justified, as using human or primate cells might provide a more relevant functional context.

We agree that validating the trans-regulatory environment is critical. The suitability of the mouse cerebellar granule cell culture system for studying human regulatory sequences was previously established in Sarropoulos et al. (*Science*, 2026 [PMID: 41610256]). In that study, we demonstrated that enhancer activity for 1:1 orthologous human and mouse CRE sequences is highly correlated, and that the trans-regulatory environment—as reflected by TF expression patterns—is well conserved between mouse granule cell cultures and human cerebellar tissue.

To further confirm this conservation in the context of the current study, we examined the expression of 28 TFs specifically active in these cell states in human, including 18 TFs whose activity is conserved across human, marmoset, and mouse in the gene regulatory network analysis using SCENIC+ (Sarropoulos et al., *Science*, 2026 [PMID: 41610256]; González-Blas et al., *Nat. Methods*, 2023 [PMID: 37443338]). We calculated the average expression of these TFs across samples with the same cell type labels and compared expression levels (log CPM) between mouse granule cell cultures and human primary tissue using 1:1 orthologous genes. The Pearson correlation between mouse granule cell culture and human primary tissues is high (0.90), indicating that TF expression

levels are well preserved (Reviewer Figure 1). These additional analyses further support that the trans-regulatory environment in mouse granule cell cultures closely resembles the in vivo condition in both mouse and human, validating this approach for testing human TE regulatory activity.

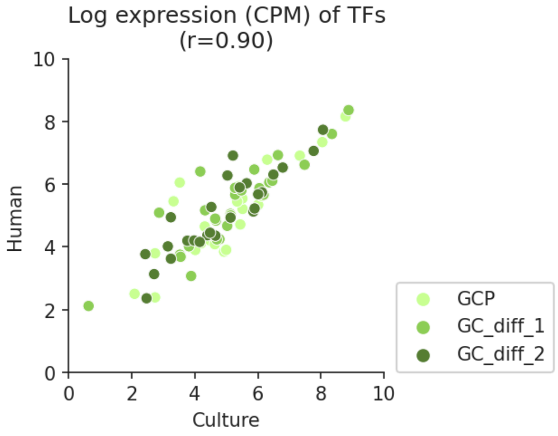

Reviewer Figure 1 | Expression levels of 28 TFs in GCP, GC_diff_1, and GC_diff_2 cell types, comparing mouse granule cell cultures to primary tissues from human. These TFs exhibit cell group-specific activity in at least one of these cell groups in the human gene regulatory network, as characterized in our previous study[1].

While human or primate cerebellar culture systems would provide a more direct test, such systems are not currently established with the same reliability and throughput as the mouse system. Our data demonstrate that the conserved trans-regulatory environment makes the mouse system a suitable proxy for this functional validation.

We have now modified the main text to better explain our rationale for using mouse granule cells as an experimental system:

> **[Page 9, Line 339]** *We previously showed that this system resembles the trans-regulatory environment of human granule cells, observing similar TF expression and enhancer activity for 1:1 orthologous human and mouse CRE sequences*[39].

- When referring to the "17 TE fragments," please clarify whether these represent individual insertions or TE families/subfamilies. Based on Figure 2, it seems they refer to TE families; if so, "family" or "subfamily" would be more accurate than "fragment."

We acknowledge that our use of "fragment" may have caused confusion. In our analysis, we divided TE ancestral consensus sequences into non-overlapping 500-bp windows—the maximum input window for our sequence-based DeepCeREvo model—to assess regulatory potential across 18 cerebellar cell states. When we previously reported 17 TE fragments, we were counting each window–cell state combination separately. We chose this approach to capture the full breadth of TE co-option across cerebellar cell states.

To improve clarity, we now (1) use "500-bp window" instead of "fragment" throughout the manuscript, and (2) report the number of subfamilies (twelve) rather than the number of individual 500-bp window–program combinations (seventeen). For TE subfamilies with one or multiple 500-bp

windows screened across multiple programs, we focused on the window–program combination with the highest $-\log_{10}$ P-value. The regulatory potential and other screening results for all 500-bp window–program combinations are described in Supplementary Table 3, so that no information is lost in practice. To clarify these points, we have revised the Results and Methods sections as follows:

> **[Page 6, Line 209]** *Our screening analysis identified twelve TE subfamilies with high cis-regulatory potential for specific cell types in cerebellum development, with enriched overlap between extant copies and cCREs accessible in the corresponding cell types (Supplementary Fig. 4; Supplementary Table 3).*

> **[Page 21, Line 757]** *We identified 500-bp windows that showed both statistically significant regulatory potential for specific cell types and enriched overlap with cCREs at corresponding positions in extant copies. This combined approach identified seventeen non-overlapping 500-bp window–program combinations from twelve TE subfamilies as candidates for co-option as cell-type-specific regulatory elements. For simplicity, we focused on one 500-bp window–program combination per TE subfamily by selecting the combination with the highest $-\log_{10}$ Q-value. The regulatory potential and other screening results for all 500-bp window–program combinations are described in Supplementary Table 3.*

- Also, while HERVL is described in very high level of detail, the other 16 TE families are not sufficiently discussed. Even a brief description of their biological relevance or cell-type-specific patterns would help readers appreciate the broader landscape of TE co-option.

In the revised manuscript, we have characterized the other 12 TE subfamilies detected in our screening in more detail. Specifically, we added the following analyses, which we describe in detail below:

1. We discuss the attribution profiles of all screened TE subfamilies and characterize the TF binding motif instances identified in their ancestral sequences.
2. Using chromatin accessibility data from the developing human brain in the first trimester (Mannens et al., *Nature*, 2024 [PMID: 38693260]), we characterize the specificity of co-option and its relevance to the associated TFs identified in the attribution analysis.

First, we characterized the attribution profiles of all twelve screened TE subfamilies, in addition to the one highlighted in the original manuscript (MER130, LTR9, HERVL, MER72, and ERVL-B4), and present these results in the Results section and a newly added Supplementary Fig. 5:

> **[Page 6, Line 217]** *The other eleven TE subfamilies also contain TF binding motif instances corresponding to their cell type enrichment. These include LTR9B in early progenitors and MER41B, MER49, and MER72 in ventricular zone neuroblasts with multiple POU motif instances (ATAT(T/G)CA) (Fig. 2d; Supplementary Fig. 5a–c). The POU TFs are known to be active during early embryonic development and their binding motif instances are enriched in LTR sequences, facilitating transcription of proviral sequences in germ lines for intergenerational inheritance[54,62,63]. We also identified HERVL, ERVL-B4, LTR1A2, MER52D, and MamRTE1 in the granule cell lineage with E-box (CAG(C/A)TG; ATOH1) and NFI*

binding motif instances (Fig. 2e; Supplementary Fig. 5d–g). ATOH1 regulates genes essential for the development of cerebellar granule cells[64–66]. Finally, MamTip2b in mature Purkinje cells contains homeodomain motif (TAATTNNNAT; LHX1/5) instances and MLT1M in microglia contains PU.1 binding motif (GGAA) instances (Supplementary Fig. 5h,i)—TFs known to specify their respective cell lineages[67,68]. Collectively, using a deep learning-based approach, we identified TE subfamilies with complex cis-regulatory sequences in their ancestral states that facilitate their preferential co-option as CREs in specific cell types.

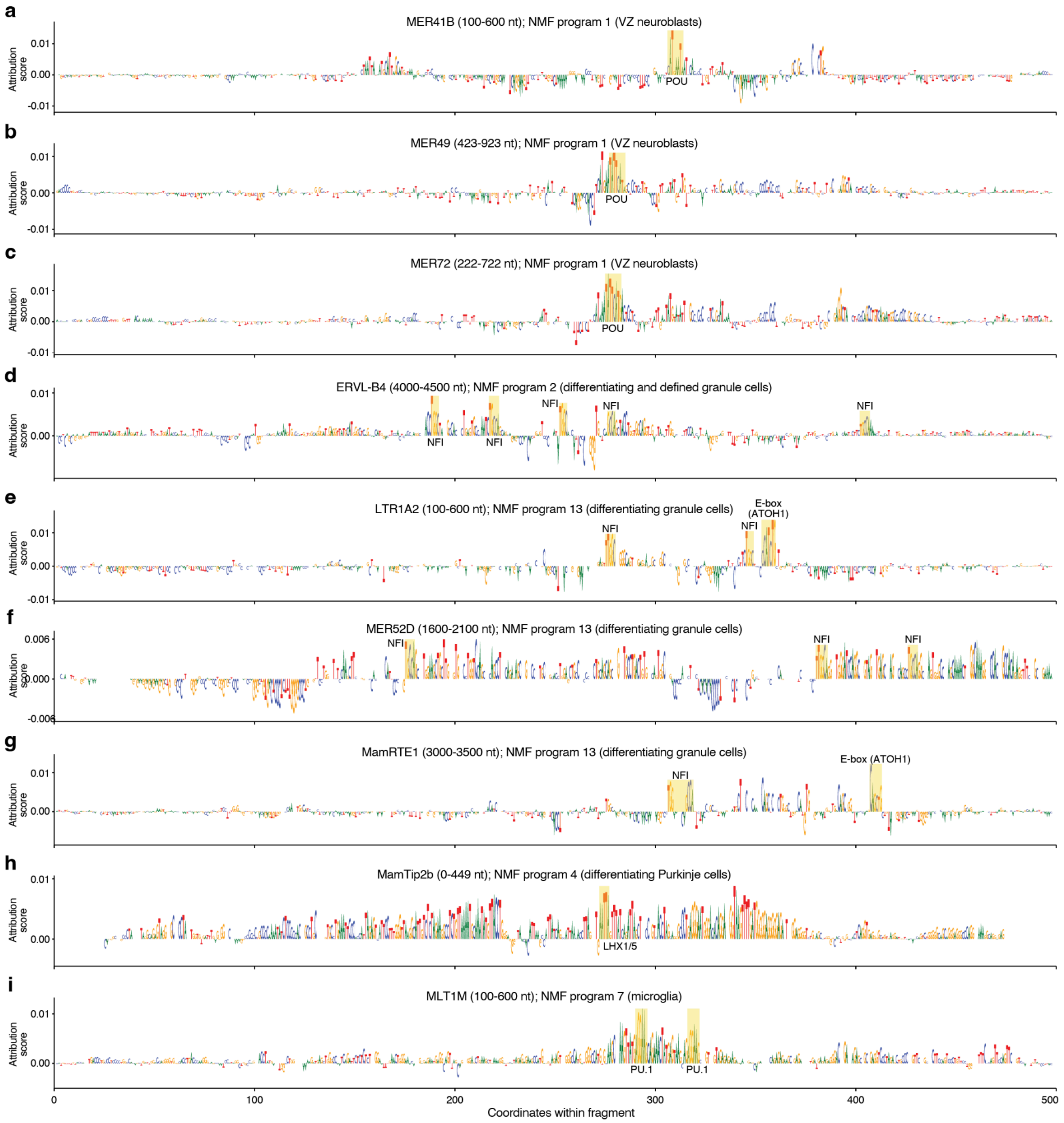

Supplementary Fig. 5 | Attribution profiles of consensus sequences from the screened TE subfamilies. a–i, *DeepExplainer attribution profiles of the consensus sequences from each screened TE subfamily highlighted in Supplementary Fig. 4.*

Second, we examined the accessibility profiles across different cell types during human brain development in the first trimester (Mannens et al., *Nature*, 2024 [PMID: 38693260]) in all screened TE subfamilies other than HERVL, and present these results in the Results section and newly added Supplementary Fig. 6b and Supplementary Fig. 7:

> **[Page 7, Line 255]** *The other TE subfamilies identified as co-opted in the granule cell lineage in our screening (ERVL-B4, LTR1A2, MER52D, MER130, and MamRTE1) also exhibit accessibility specific to rhombic lip–derived glutamatergic neuroblasts, though lineage specificity varies between subfamilies (Supplementary Fig. 6b). Evolutionarily older elements (MER130 and MamRTE1) showed broader enrichment across different cell lineages, particularly in glutamatergic neurons and late neural progenitors. The other TE subfamilies identified in our screening also show lineage-specific accessibility corresponding to their TF binding motifs instances. LTR9B, MER41B, MER49, and MER72, which contain POU binding motif instances and are enriched in cCREs specific to early cerebellar progenitors or ventricular zone neuroblasts (Fig. 2d; Supplementary Fig. 5a–c), are accessible in radial glia and glioblasts across brain regions, as well as in telencephalic glutamatergic neurons (Supplementary Fig. 7a,b). These patterns are consistent with the established roles of POU family TFs, particularly POU3 TFs, in neural progenitors and cortical neurons[78,79]. Among older TEs that predate the mammalian common ancestor, MamTip2b and MLT1M are specifically accessible in the Purkinje cell and microglial lineages, respectively, matching the lineage-specific expression of LHX1 and SPI1, respectively (Supplementary Fig. 7a,b). These results indicate that the identified TE subfamilies are co-opted in specific cell lineages where TFs that recognize their corresponding motifs are active.*

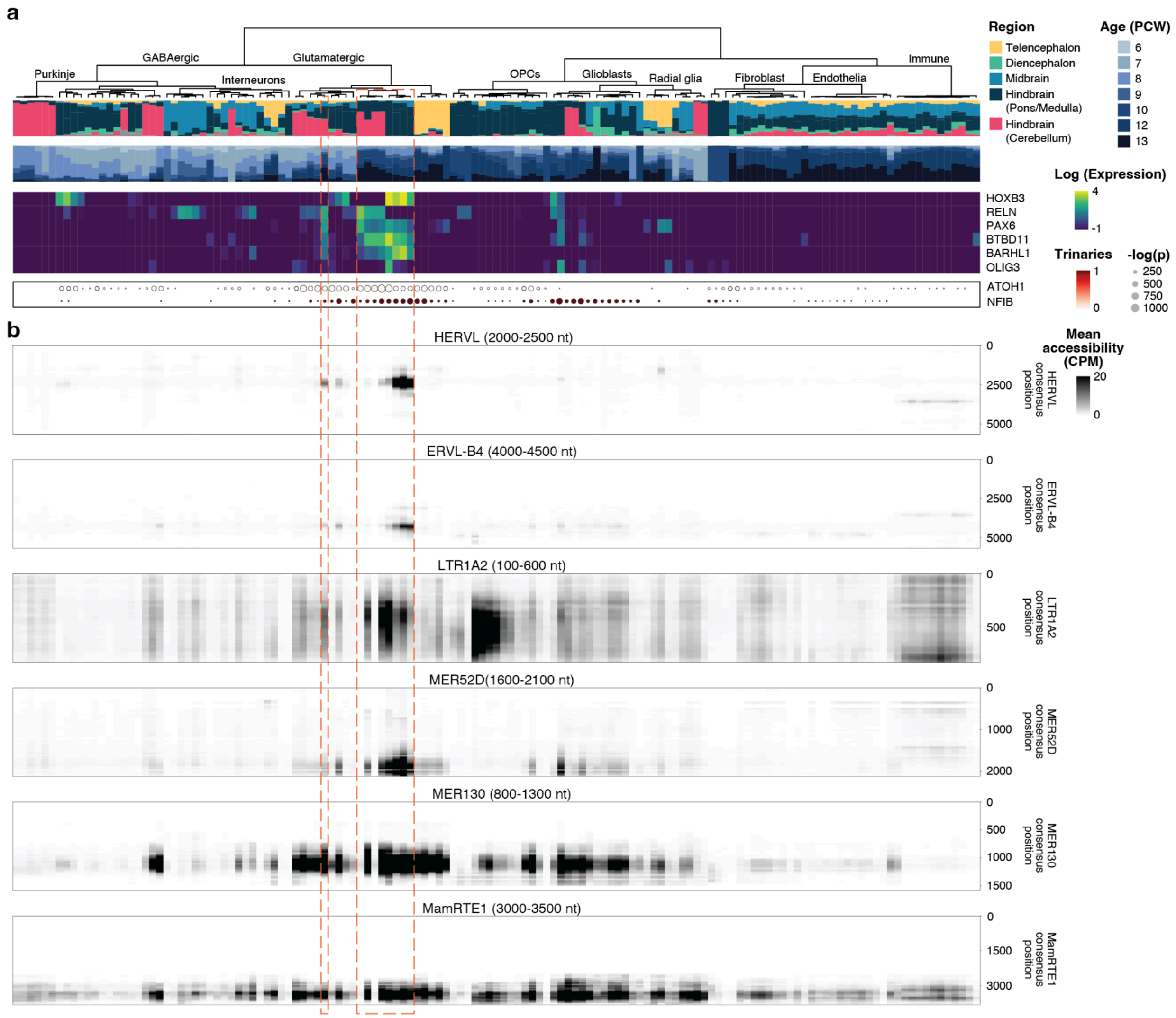

Supplementary Fig. 6 | <u>Hindbrain glutamatergic neuroblast-specific accessibility of the screened TE subfamilies.</u> a, *Top to bottom: hierarchical clustering dendrogram showing relationships between cell types based on highly variable cCREs; anatomical region distribution showing the proportion of each cell type across brain regions; developmental age distribution showing temporal profiles of each cell type; expression heatmap of selected marker genes across cell types; TF binding motif enrichment analysis with dot size representing statistical significance (P-values) and color intensity representing expression levels of the corresponding TFs (trinarization score represents a probabilistic measure of gene expression ranging from 0-1). Highlighted cell types show HERVL accessibility (Fig. 3d). Data from Mannens et al., 2024[2] reanalyzed.* *<u>b. Mean accessibility profiles of the 50 most accessible copies of each of the six TE subfamilies identified as specifically co-opted in the granule cell lineage (Supplementary Fig. 4), across diverse cell types from different brain regions at various developmental stages (data from Mannens et al., 2024[2]), aligned to the consensus sequence of the respective TE subfamily.</u>*

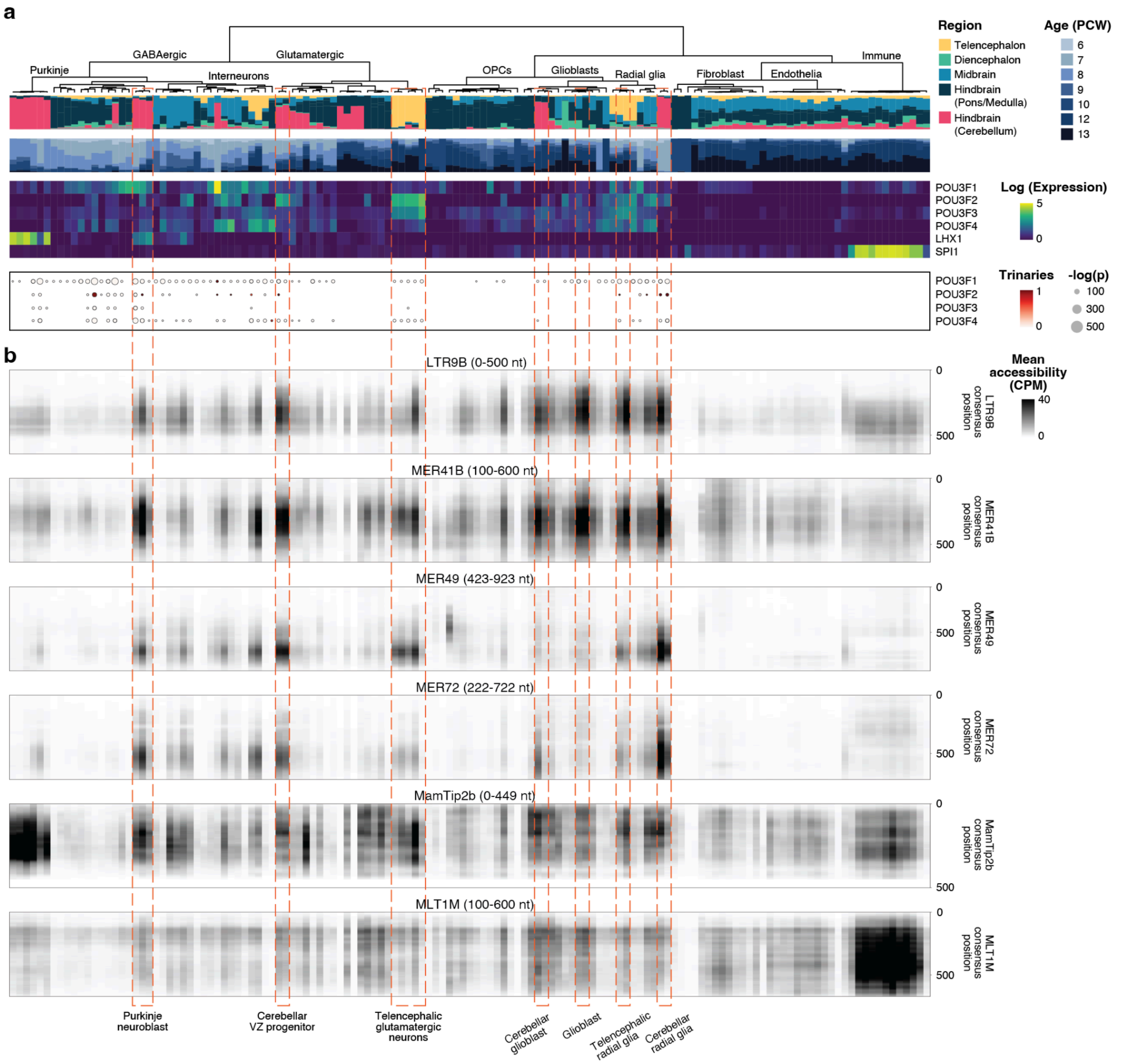

***Supplementary Fig. 7 | Lineage-specific accessibility of the screened TE subfamilies. a,** Cell type characterization as in Supplementary Fig. 6a. **b**, Mean accessibility profiles of the 50 most accessible copies of each of the six TE subfamilies identified as specifically co-opted in neural progenitors/neuroblasts (LTR9B, MER41B, MER49, and MER72), Purkinje cells (MamTip2b), and microglia (MLT1M) (Supplementary Fig. 4), across diverse cell types from different brain regions at various developmental stages (data from Mannens et al., 2024[2]), aligned to the consensus sequence of the respective TE subfamily.*

The increased accessibility in different cell lineages can be explained by the regulatory potential of the corresponding elements in the relevant cell states, as described for HERVL in the original manuscript. In the revised manuscript, we have added a similar analysis for LTR1A2, newly added Supplementary Fig. 8c,d, and extended the discussion as follows:

> **[Page 8, Line 274]** *The same TE subfamily can also be co-opted as regulatory elements in distinct cell types[29]. Microglia and cortical radial glia exhibit distinct accessibility patterns at different positions within the HERVL consensus sequence, a pattern consistent across*

datasets (Fig. 3d). Indeed, the 3,300–3,800 nt position of HERVL, where microglia show enriched accessibility, displays high regulatory potential in microglia and contains two PU.1 binding motif instances (Supplementary Fig. 8a,b). Similarly, LTR1A2 shows enriched accessibility not only in rhombic lip-derived neuroblasts but also in oligodendrocyte precursor cells (Supplementary Fig. 6a,b), and its ancestral sequence exhibits high regulatory potential in the corresponding program and contains binding motif instances for SOX TFs, which are known to be important for oligodendrocyte differentiation[80] (Supplementary Fig. 8c,d). These results suggest that, although we focused on twelve TE subfamilies based on conservative criteria, additional instances of TE co-option likely exist, including multiple co-option events within the same subfamily driven by distinct sequence features.

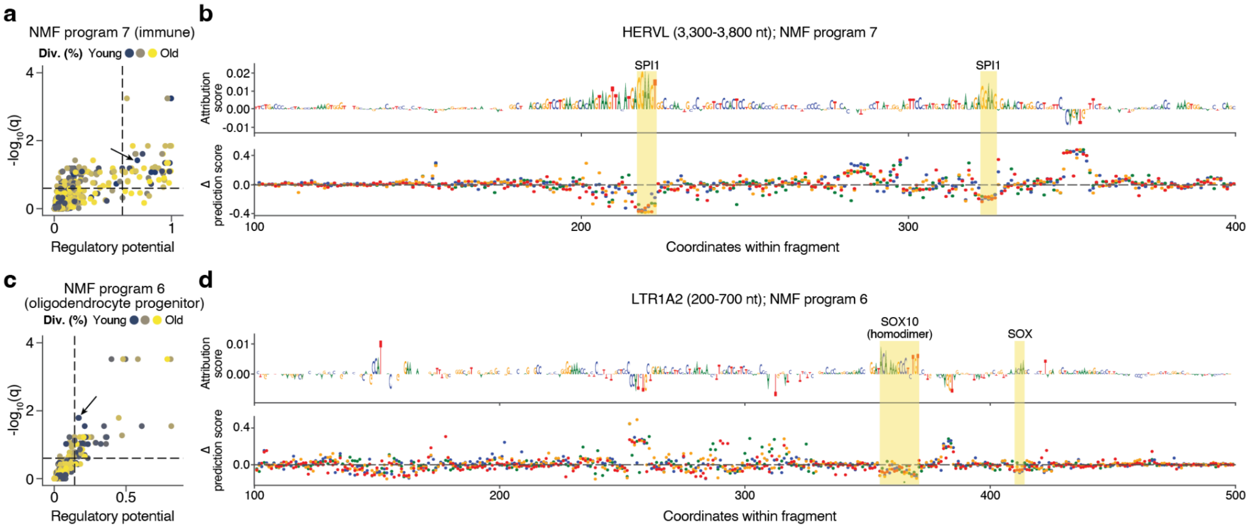

Supplementary Fig. 8 | Sequence features explaining increased accessibility in distinct cell types. a, c, *Regulatory potential scores and associated Q-values in microglia (program 7) (**a**) and oligodendrocyte progenitors (program 6) (**c**) for all TE fragments. In contrast to Supplementary Fig. 4, TE fragments with fewer than 10 overlaps with highly variable cerebellar cCREs are also shown.* ***b, d,*** *DeepExplainer attribution profiles in microglia for HERVL at the 3,300–3,800 nt position (**b**) and in oligodendrocyte progenitors for LTR1A2 at the 200–700 nt position (**d**), which are indicated by an arrow in (**a**) and (**c**), respectively.*

Collectively, these additional analyses on the screened TE subfamilies beyond HERVL strengthen the generalizability of our findings and help readers appreciate the broader landscape of TE co-option. We have also added further analyses on these TE subfamilies, as documented in the response to major point 2 for reviewer #2 (page 25 of this response letter).

- The discussion is short and doesn't really add much to the interpretation of the results.

We have expanded the Discussion to strengthen the interpretation of our results. Specifically, we have:

- More explicitly highlighted the novelty of our finding of preferential TE co-option from internal sequences of endogenous retroviruses:

  [Page 14, Line 510] *Among these, HERVL and ERVL-B4 are internal sequences of endogenous retroviruses (ERV-int), suggesting that readily co-optable regulatory sequences can also arise in TE classes not canonically associated with regulatory activity.*

- Situated our work more explicitly within the context of individual prior studies by incorporating additional citations and discussing their relevance to our findings:

  [Page 14, Line 512] *We also revealed that some of these preferentially co-opted TE subfamilies, as shown for HERVL and LTR1A2, harbor other sequence features that drive co-option in different cell types, as recently reported for the rodent-specific ORR1D2 and ORR1E subfamilies in immune cell types[29]. Furthermore, we used DeepCeREvo to show that preservation of ancestral sequences (and in some cases, acquisition of additional substitutions) and proximity to pre-existing active chromatin contribute to co-option at the individual copy level, in line with observations of RSINE1 co-opted as a circadian enhancer in mouse[20].*

- Included a broader interpretation of our findings on TE-driven regulatory evolution in comparison to non-TE-driven regulatory evolution:

  [Page 14, Line 529] *These patterns are consistent with evolutionary processes—mutation, natural selection, and genetic drift—where inserted TE copies serve as raw material for regulatory evolution similarly to other non-TE-derived sequences, rather than providing a "shortcut" via pre-adaptive sequences in their ancestral state. This model is further supported by older TE subfamilies from LINE, SINE, and DNA classes showing higher cCRE enrichment than younger ones, while the absence of this age dependence in TE subfamilies from LTR and ERV-int classes points instead to preferential co-option via pre-existing regulatory sequences.*

- Leveraged the expanded analyses suggested by the reviewers to draw more generalizable conclusions from our results:

  [Page 14, Line 519] *These lines of evidence further illustrate the multifaceted nature of TE co-option as regulatory elements, while also demonstrating the utility of sequence-based models in understanding this process at cell-type resolution across TE subfamilies.*

[Page 14, Line 536] *Together, these observations highlight that understanding TE contributions to regulatory evolution requires considering both evolutionary constraint and preferential co-option driven by ancestral sequence features.*

- Added a discussion of limitations, particularly the input window constraint of our model, which points to promising directions for future work:

  [Page 15, Line 544] *An additional limitation is the relatively small input window (500 bp) of our sequence-based model, DeepCeREvo. This input size is sufficient to capture local sequence features such as TF binding motif instances, but a longer input window would allow for incorporating longer stretches of ancestral sequence, making it possible to evaluate the regulatory potential of long TE classes (e.g., LINE, ERV-int) as a whole, as well as the surrounding genetic context of extant TE copies, which could shed light on the effect of surrounding regulatory elements more clearly. Applying a sequence-based model with a larger input window, such as Borzoi[97] or AlphaGenome[98] (524 kb and 1 Mb input windows, respectively), to TE studies represents a promising direction for future research.*

We believe these revisions provide richer interpretation for a broader readership and more clearly articulate the contributions of our study relative to existing literature.

Reviewer: 2

The manuscript by Yamada et al. examines the contribution of transposable elements (TEs) to the gene regulatory evolution coordinated by cis-regulatory elements (CREs) within the mammalian cerebellum. These findings add to a growing body of cross-species single-cell multiomics studies evaluating TE regulatory activity (e.g., (Simon et al., Li et al., Zemke et al.). The work substantially leverages the authors' previous work with the DeppCeREvo model. The HERVL case study is the main empirical novelty. As written, the manuscript is quite well written and my concerns are only modest ones on two issues limit generality: (i) enrichment framing without full treatment of caveats, and (ii) heavy emphasis on HERVL relative to the stated aim of generalizable TE principles.

We appreciate the reviewer's positive assessment of our study and the recognition that our findings contribute to the growing body of cross-species single-cell multiomics work on TE regulatory activity. In our revised manuscript, we have provided extensive additional analyses and discussion to address the two concerns raised, specifically regarding the enrichment framing and the generalizability of our findings beyond the HERVL case study.

MAJOR POINTS

1-1) The central patterns rely on overlap/enrichment. Please foreground limitations and add controls. In particular, while it is not uncommon in the field to present simple depletion of enrichment as, itself, a signal (and this is quite plausible biologically), it really isn't a great practice from a statistical or even logical perspective. Perhaps that can be handled textually but there are some improved benchmarking or controls that could be done throughout. E.g., Define background for each comparison and report multiple backgrounds if possible with controls where appropriate (e.g., length matched, distance-to-TSS-matched, etc). Consider replacing some enrichment with regression where possible.

We thank the reviewer for this suggestion to strengthen the statistical rigor of our previous approach. We have now implemented logistic regression models with several control variables (detailed below for each analysis) for all major comparisons, clearly defined backgrounds, and reported odds ratios with confidence intervals and *P*-values. The detailed results of all regression analyses are provided in Supplementary Tables 1 and 5.

Importantly, these extensive updates confirm and strengthen our original biological conclusions, and all major findings remain significant and effect sizes are consistent with our initial observations, demonstrating the robustness of our results.

Below we detail the improvements for each analysis in the order they appear in the manuscript.

1. TE depletion in cCREs compared to genomic background

   We replaced our previous approach of comparing observed TE overlap (17.6%) to genome-wide abundance (45%) with a rigorous logistic regression framework. We have revised the manuscript as follows:

   [Page 3, Line 93] *To determine whether this represents TE depletion compared to genomic abundance of TEs (~45%)[21], we compared cCREs to length-matched random genomic regions using logistic regression, controlling for GC content, mappability, and distance to the transcription start site of the nearest gene. After accounting for these genomic features, cCREs showed significant TE depletion (odds ratio [OR] = 0.38, $P < 10^{-15}$), representing a 62% reduction in the odds of TE overlap relative to the genomic background (Supplementary Table 1).*

2. TE enrichment by regulatory element type

   We replaced simple percentage comparisons with logistic regression modeling TE overlap as a function of regulatory element type, controlling for conservation category and GC content. We have revised our manuscript as follows:

   [Page 3, Line 99] *Within cCREs, TE overlap varied significantly by regulatory element type. Distal cCREs exhibited the highest TE overlap (23.8%) (Fig. 1b). Relative to distal elements, exonic cCREs showed the strongest TE depletion (OR = 0.27, $P < 10^{-15}$), followed by promoters (OR = 0.67, $P < 10^{-15}$) and intronic elements (OR = 0.78, $P < 10^{-15}$) (Supplementary Table 1). These patterns reflect the differential functional constraints of different regulatory element classes[41].*

3. TE enrichment in species-specific cCREs

   We replaced simple percentage comparisons with logistic regression modeling TE overlap within cCREs as a function of conservation level, with cCREs classified as human-specific, primate-specific, mammalian-conserved (shared across human, marmoset, and mouse), or unclassified (reference category), controlling for peak type and GC content. We have revised our manuscript as follows:

   [Page 3, Line 106] *To investigate the relationship between TE contributions to regulatory elements and evolutionary constraint, we used cerebellar cCREs from marmoset (n = 505,724) and mouse (n = 498,119)[39] to classify human cCREs as human-specific, primate-specific, mammalian-conserved, or unclassified. Using logistic regression controlling for peak type and GC content, lineage-specific cCREs showed strong TE enrichment relative to unclassified cCREs: human-specific cCREs had 62% TE overlap (OR = 6.25, $P < 10^{-15}$) and primate-specific cCREs had 29% TE overlap (OR = 1.82, $P < 10^{-15}$) (Fig. 1c; Supplementary Table 1). Conversely, mammalian-conserved cCREs showed only 5% TE overlap (OR = 0.24, $P < 10^{-15}$), though the contributions of older TEs to conserved cCREs may be underestimated due to difficulty of annotating highly degenerated TEs[42].*

   We have also updated Fig. 1c to match the comparison in the regression framework:

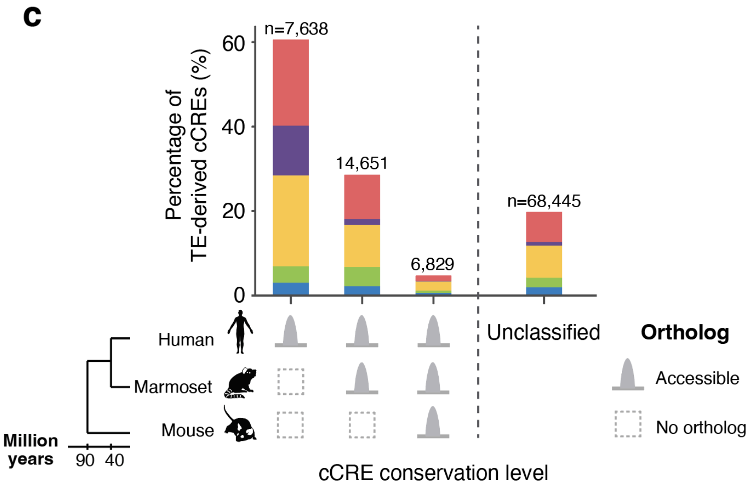

Fig. 1c | Percentage of TE-derived human cCREs across accessibility conservation levels between human, marmoset, and mouse.

4. TE enrichment in cell type-specific cCREs

We replaced developmental stage comparisons based on raw percentages with logistic regression modeling TE overlap as a function of developmental accessibility state, controlling for peak type, GC content, and conservation (accessibility conservation category for cerebellar data; sequence conservation for the multi-organ dataset). We have revised our manuscript as follows:

[Page 4 Line 132] *Using logistic regression to model TE overlap while controlling for peak type, conservation, and GC content, we found that TE content decreases progressively as cCREs are shared across more cell types. Elements shared across 2 programs showed 15% lower odds of TE overlap than cell-type-specific elements (OR = 0.85, P < 10^{-15}), with the reduction increasing to 70% for elements shared across more than 9 programs (OR = 0.30, P < 10^{-15}) (Fig. 1d; Supplementary Table 1).*

5. TE enrichment in earlier developmental stages

We replaced developmental stage comparisons based on raw percentages with logistic regression modeling TE overlap as a function of developmental accessibility state, controlling for peak type, conservation category, and GC content. We have revised our manuscript as follows:

[Page 4, Line 142] *The contributions of TEs to cell-type-specific cCREs vary markedly across developmental stages (Fig. 1e). After controlling for peak type, GC content, and conservation category, cCREs accessible during embryonic development (~8 weeks post conception) showed 58% lower odds of TE overlap than adult-accessible elements (OR = 0.42, P < 10^{-15}), and fetal-accessible cCREs (9–40 weeks post conception) showed 41% lower odds of TE overlap (OR = 0.59, P < 10^{-15}) (Supplementary Table 1). To determine whether this pattern applies more broadly across cell types and organs, we re-analyzed data from a previously published single-cell chromatin accessibility dataset[48] using logistic regression*

controlling for peak type, GC content, and sequence conservation. This analysis confirmed that fetal-accessible cCREs exhibit 18% lower odds of TE overlap than adult-accessible elements (OR = 0.82, $P < 10^{-15}$) (Fig. 1f; Supplementary Table 1).

6. Predictors of HERVL regulatory activity

We implemented multivariate logistic regression to quantify the relative contributions of multiple factors to HERVL accessibility, modeling accessibility status as a function of sequence features (regulatory prediction score, GC content, length, divergence), genomic context (distance to TSS, distance to nearest cell-type-specific cCRE), KRAB-ZNF binding, and A/B compartment positioning. We have added Fig. 5m and the following sentences to the manuscript:

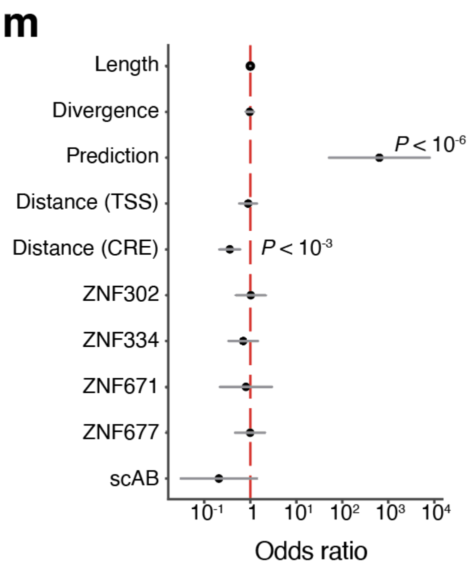

***Fig. 5m** | Odds ratios and 95% confidence intervals for each covariate in logistic regression models predicting accessibility of individual HERVL copies.*

[Page 11, Line 395] *To integrate these observations and quantify the relative contributions of all factors examined above, we performed multivariate logistic regression. This analysis confirmed that DeepCeREvo prediction score (OR = 1,205, $P < 10^{-6}$) and proximity to cell-type-specific cCREs (OR = 0.28 per log-unit increase in distance, $P < 10^{-4}$) are the dominant predictors of HERVL accessibility, while other factors showed no significant independent effects (Fig. 5m; Supplementary Table 5).*

1-2) Some of the multiple testing control is not clear enough.

Thank you for raising this point. We have revised our multiple testing correction procedure to improve rigor and clarity.

Because our screening examines overlapping 500-bp windows tiled at 100-bp intervals along TE consensus sequences (Fig. 2a), adjacent fragments share 80% (400 bp) of their input sequence, resulting in highly correlated *P*-values between neighboring tests. For instance, for program 3 (progenitor_gliogenic), $-\log_{10}$ *P*-values of adjacent fragments show a Pearson correlation of 0.66 (Reviewer Figure 2). The effective number of independent tests is therefore smaller than the actual number of tests, and applying standard multiple testing correction to all tests would therefore be overly conservative, as many tests are not independent. To account for this, we used the effective number of independent tests (m_eff), a concept widely applied in genome-wide association studies and other multilocus analyses (Li and Ji, *Heredity*, 2005 [PMID: 16077740]; Hahn, *Mol Biol Evol.*, 2006 [PMID: 16407459]).

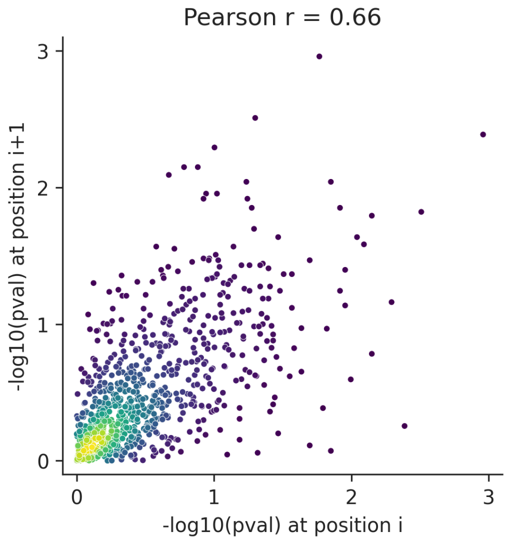

Reviewer Figure 2 | Correlation of -$\log_{10}$ *P* values between adjacent positions of TE fragments for program 3 (progenitor_gliogenic).

In the revised manuscript, we now compute Benjamini–Hochberg adjusted *Q*-values and report these FDR-controlled values directly rather than applying a modified *P*-value threshold. In addition, we have replaced the previous ad hoc correction for test dependence with a principled, empirically estimated effective number of tests (m_eff) based on the autocorrelation structure of test statistics across adjacent TE fragments (Hahn, *Mol Biol Evol.*, 2006 [PMID: 16407459]; Bayley and Hammersley, *J. Roy. Stat. Soc.*, 1946). The estimated correction factors (M/m_eff) range from 1.6 to 7.2 across programs. We then incorporated m_eff into the modified Benjamini–Hochberg procedure following the previous study (Li and Ji, *Heredity*, 2005 [PMID: 16077740]). We call fragments significant at FDR < 20%, a lenient threshold appropriate for exploratory genomic screens, though eleven of the twelve identified TE subfamilies also meet the more stringent threshold of FDR < 10%. With this revised statistical framework, we identified the same seventeen 500-bp window–program combinations from twelve TE subfamilies as in our previous approach (Supplementary Fig. 4; Supplementary Table 3). The updated Methods section now describes this procedure in detail, and we have updated the Supplementary Fig. 4 accordingly:

[Page 20, Line 710] *To determine statistical significance, we shuffled each TE fragment 1,000 times and computed regulatory potential for these shuffled sequences. By comparing regulatory potential between original and shuffled fragments, we calculated empirical P-values and applied the Benjamini–Hochberg procedure to compute Q-values. Because our screening examines overlapping 500-bp windows tiled at 100-bp intervals along TE consensus sequences, adjacent fragments share 80% (400 bp) of their input sequence, resulting in highly correlated P-values between neighboring tests. Applying standard multiple testing correction to all tests would therefore be overly conservative, as many tests are not independent[100,101].*

To estimate the effective number of independent tests (m_{eff}), we used the classical autocorrelation-based formula for temporally or spatially correlated data[102], which has been applied to genomic analyses with similar correlation structure[101]. This method exploits the natural linear ordering of fragments along the TE consensus sequence and does not require multiple observations per test to estimate pairwise correlations. The formula is:

$$m_{eff} = M / (1 + 2\sum \rho(k)),$$

where M is the total number of tests and $\rho(k)$ is the autocorrelation at lag k. For each program, we ordered TE fragments by their position along the consensus sequence and computed the autocorrelation of $-\log_{10}$ P-values. Following standard practice, we summed autocorrelations over positive lags until the first non-positive value, which serves as a natural truncation point[103]. The resulting correction factors (M/m_{eff}) ranged from 1.6 to 7.4 across programs.

We incorporated m_{eff} into the Benjamini–Hochberg procedure following the previous study[100]. Briefly, in the standard procedure, ordered P-values are compared against thresholds that increase linearly from α/M to α, where α is the target FDR level (e.g., 0.10 for 10% FDR). To account for correlated tests, we replaced the starting threshold α/M with α/m_{eff} while keeping the endpoint at α, thereby relaxing the correction to reflect the reduced number of truly independent tests. We called fragments significant at FDR < 20%, though eleven of the twelve identified TE subfamilies also meet the more stringent threshold of FDR < 10%.

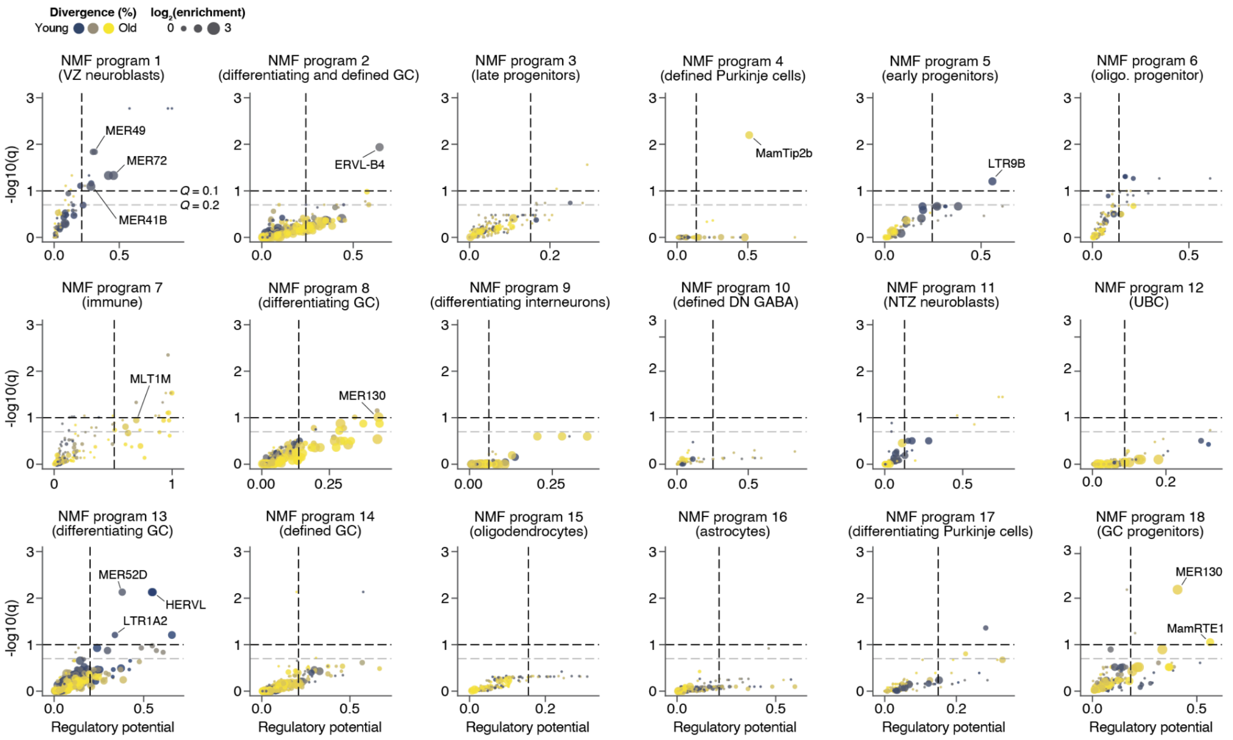

Supplementary Fig. 4 | In silico screening of transposable element fragments with high regulatory potential in human cerebellar development. *Screening results for TE fragments showing regulatory potential scores and associated* $-\log_{10}$ *Q-values (P-values corrected by Benjamini–Hochberg procedure), alongside enrichment in cell-type-specific cCREs for each program. The regulatory potential threshold was set at the 95th percentile. The Q-value threshold was set at 0.10 and 0.20, corresponding to a false discovery rate (FDR) of 10% and 20%, respectively. The screened TE subfamilies are highlighted by the fragment with the highest* $-\log_{10}$ *Q-value. VZ, ventricular zone; NTZ, nuclear transitory zone; GC, granule cell; UBC, unipolar brush cell; DN GABA, deep nuclei GABAergic neuron.*

In addition, we now report the number of subfamilies (twelve) instead of the number of 500-bp window–program combinations (seventeen) for clarity. We have revised the Results and Methods sections as follows:

> **[Page 6, Line 209]** *Our screening analysis identified twelve TE subfamilies with high cis-regulatory potential for specific cell types in cerebellum development, with enriched overlap between extant copies and cCREs accessible in the corresponding cell types (Supplementary Fig. 4; Supplementary Table 3).*
>
> **[Page 21, Line 757]** *We identified 500-bp windows that showed both statistically significant regulatory potential for specific cell types and enriched overlap with cCREs at corresponding positions in extant copies. This combined approach identified seventeen non-overlapping 500-bp window–program combinations from twelve TE subfamilies as candidates for co-option as cell-type-specific regulatory elements. For simplicity, we focused on one 500-bp window–program combination per TE subfamily by selecting the combination with the highest* $-\log_{10}$ *Q-value. The regulatory potential and other screening results for all 500-bp window–program combinations are described in Supplementary Table 3.*

> *We identified 500-bp windows that showed both statistically significant regulatory potential for specific cell types and enriched overlap with cCREs at corresponding positions in extant copies. This combined approach identified seventeen non-overlapping 500-bp window–program combinations from twelve TE subfamilies as candidates for co-option as cell-type-specific regulatory elements. For simplicity, we focused on one 500-bp window–program combination per TE subfamily by selecting the combination with the highest* $-\log_{10}$ *P-value. The regulatory potential and other screening results for all 500-bp window–program combinations are described in Supplementary Table 3.*

2) As initially described, this is a general paper, but the narrative is unusually focused on a specific use case. That use case (HERVL) is interesting and well described but not prominent or well grounded enough to be a paper. I think the manuscript should probably be expanded beyond a single case study. There are other TE fragments suggested in the figures and the same minimal functional validation would be valuable for, perhaps, two more.

We have now added detailed characterization of the other screened TE subfamilies. The additional analyses—spanning attribution profiling, chromatin accessibility characterization, determinants of copy-level co-option, and cross-species divergence—extend our findings well beyond the HERVL case study and show that our findings generalize across multiple TE subfamilies with diverse evolutionary origins, cell-type specificities, and TF dependencies.

We first characterized the attribution profiles of all screened TE subfamilies along with the TF binding motif instances identified in their ancestral sequences, and then examined their accessibility patterns comprehensively using chromatin accessibility data from the developing human brain in the first trimester (Mannens et al., *Nature*, 2024 [PMID: 38693260]). These analyses are documented in detail in the response to a comment from reviewer #1 (page 15 of this response letter). Briefly, we have added DeepCeREvo attribution analyses for all twelve identified TE subfamilies (Page 6, Line 218; Supplementary Fig. 5) and characterized the cell type-specificity of TE co-option in the developing human brain during the first trimester (Mannens et al., *Nature*, 2024 [PMID: 38693260]) (Page 7, Line 258; Supplementary Figs. 6, 7).

We then investigated determinants of copy-level TE co-option for the screened TE subfamilies other than HERVL. We have added the following text, Supplementary Figs. 11–13, and Supplementary Tables 5 and 6 to our revised manuscript:

> **[Page 11, Line 404]** *We next examined whether these patterns extend to other screened TE subfamilies. Multivariate logistic regression revealed that DeepCeREvo prediction scores are the strongest predictors for all subfamilies, that proximity to non-TE cCREs contributes significantly in 75% of subfamilies, and that KZFP binding shows significant effects in only 25% (Supplementary Fig. 12; Supplementary Table 5). The predictive power of DeepCeREvo scores reflects ancestral motif preservation: in young TE subfamilies, sequences at ancestral TF binding motif positions are recurrently used and significantly more preserved in*

accessible copies (Supplementary Fig. 13a–e; $P < 0.05$, linear regression), whereas in older subfamilies, these positions show significantly stronger purifying selection across 447 mammalian genomes[86–88] (Supplementary Fig. 14a–d, $P < 0.05$, linear regression) (Supplementary Table 6).

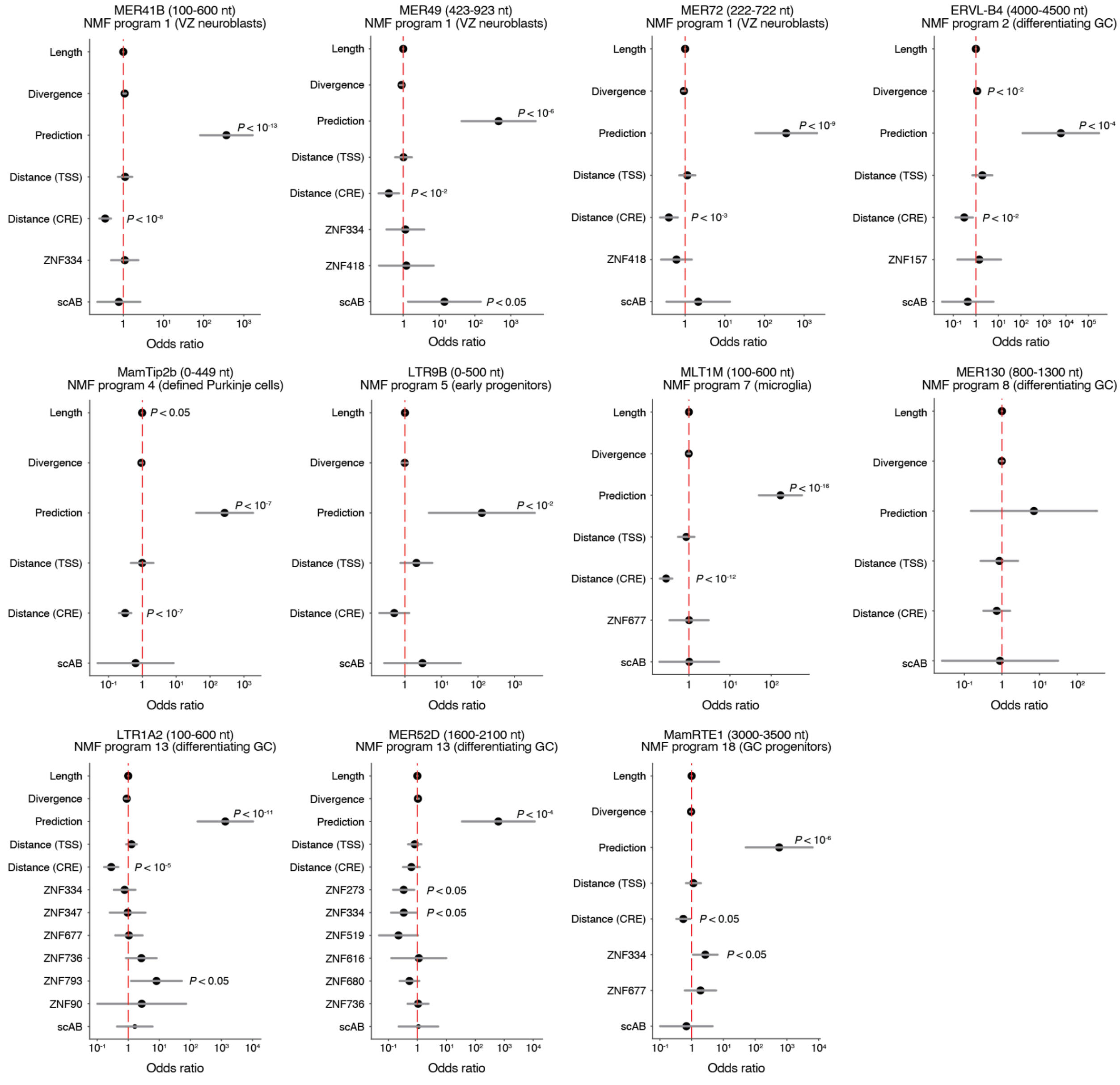

Supplementary Fig. 12 | Determinants of copy-level TE co-option for the screened TE subfamilies. Odds ratios and 95% confidence intervals for each covariate in logistic regression models predicting accessibility of individual copies of the screened TE subfamilies.

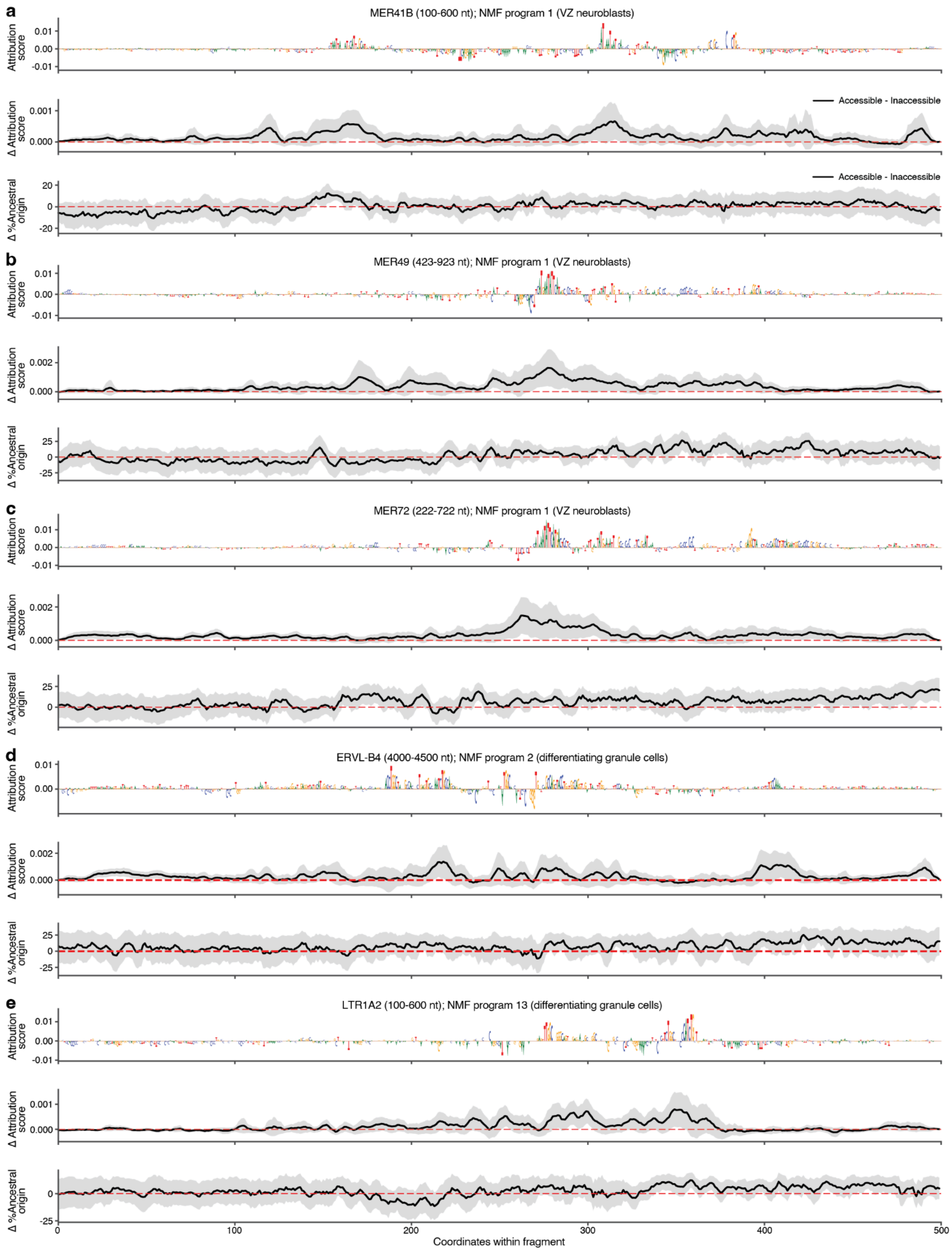

Supplementary Fig. 13 | Recurrent usage of ancestral TF motif instances and preservation of ancestral nucleotides in accessible copies. a–e. *DeepExplainer attribution profile of the consensus sequence of the TE subfamily (top). Difference in attribution profiles between accessible and inaccessible copies, aligned to the*

*consensus sequence, with the 95% confidence interval shown (middle). Difference in the percentage of nucleotides matching the consensus sequence between accessible and inaccessible copies, aligned to the consensus sequence (bottom). Plots are shown for MER41B (**a**), MER49 (**b**), MER72 (**c**), ERVL-B4 (**d**), and LTR1A2 (**e**).*

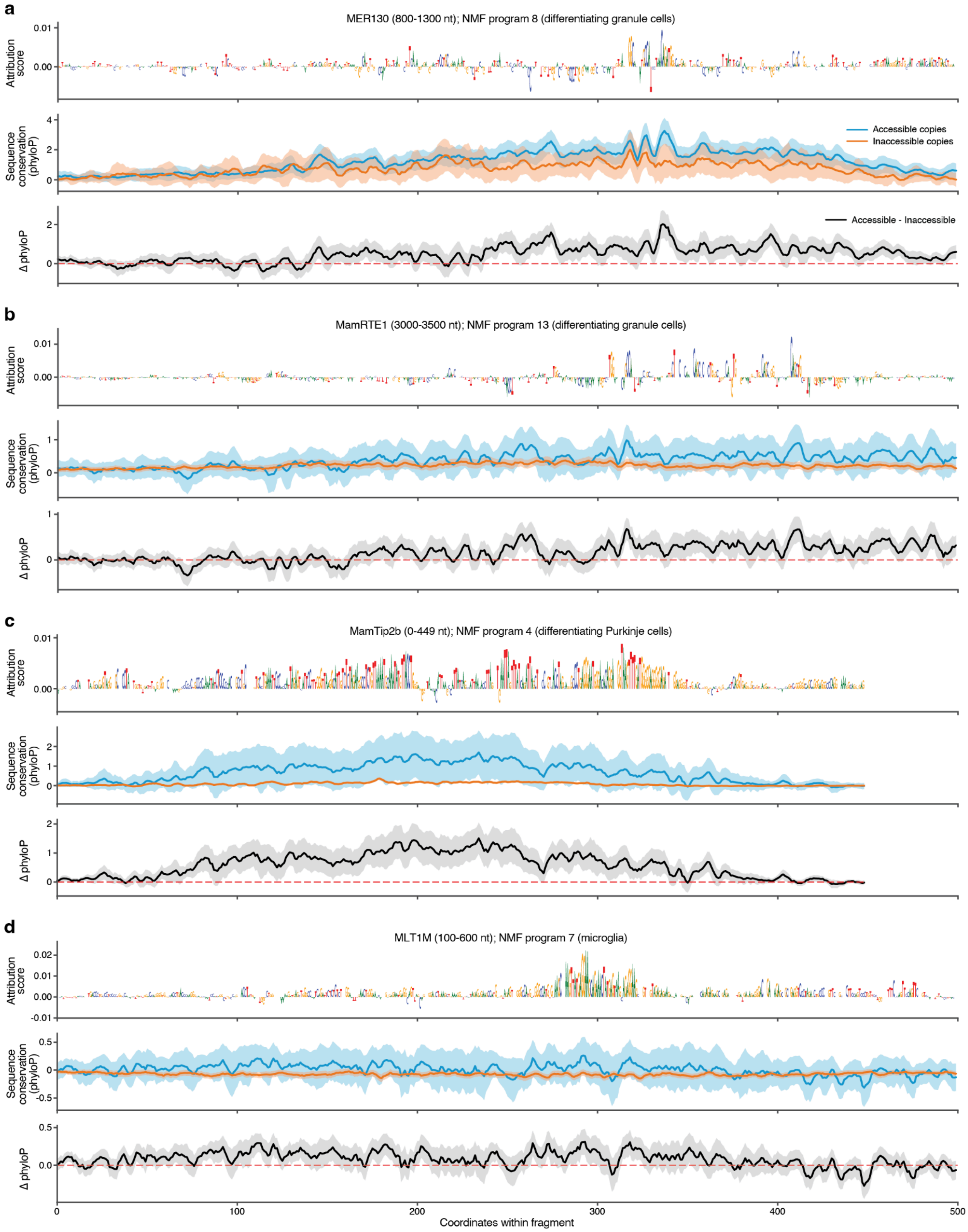

Supplementary Fig. 14 | Cross-mammalian conservation of sequences at ancestral TF binding motif instances in older screened TE subfamilies. a–d, *DeepExplainer attribution profile of the consensus sequence of the TE subfamily (top). Attribution profiles of accessible and inaccessible copies (middle) and the difference between them (bottom), aligned to the consensus sequence, with the 95% confidence interval shown. Plots are shown for MER130 (**a**), MamRTE1 (**b**), MamTip2b (**c**), and MLT1M (**d**).*

We also examined the impact of species-specific insertions from the screened TE subfamilies on gene expression divergence. We focused on LTR1A2 and MER52D in addition to HERVL — all three being primate-specific elements enriched in differentiating granule cells, a cell state well-captured across all four species. We have revised our manuscript as follows:

> **[Page 12, Line 437]** *To test whether this occurs during primate brain development, we analyzed our previously generated single-cell transcriptome and chromatin accessibility datasets from macaque, marmoset, and mouse cerebellum, focusing on HERVL, LTR1A2, and MER52D—primate-specific elements enriched in differentiating granule cells, a cell state well-captured across all four species[39].*

We show that all three TE families exhibit similar properties to HERVL: accessible copies are predominantly species-specific, and these lineage-specific insertions contribute to gene expression divergence between species. Accordingly, we have added Supplementary Figs. 17, 18, and 20.

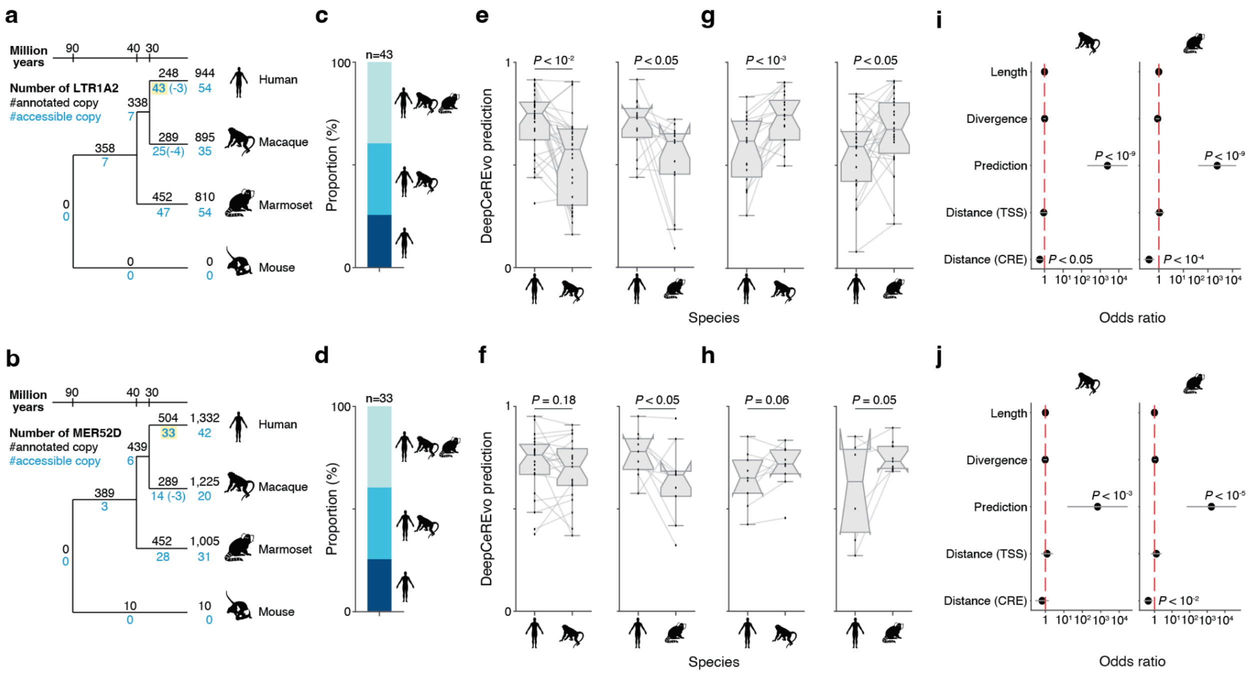

Supplementary Fig. 17 | Species-specific co-option of LTR1A2 and MER52D in differentiating granule cells. a, b, *Number of annotated and accessible copies of LTR1A2 (**a**) and MER52D (**b**) in differentiating granule cells (GC_diff_1) across human, macaque, marmoset, and mouse.* ***c, d,*** *Insertion age distribution of LTR1A2 (**c**) and MER52D (**d**) copies that are accessible in differentiating granule cells specifically in human.* ***e, f,*** *DeepCeREvo prediction scores for differentiating granule cells (program 13) comparing accessible copies of LTR1A2 (**e**) and MER52D (**f**) in human with their corresponding orthologous sequences in macaque*

(left) and marmoset (right). ***g, h,*** *DeepCeREvo prediction scores for differentiating granule cells (program 13) comparing accessible copies of LTR1A2 (**g**) and MER52D (**h**) in macaque (left) and marmoset (right) with their corresponding orthologous sequences in human.* ***i, j,*** *Odds ratios and 95% confidence intervals for each covariate in logistic regression models predicting accessibility of individual LTR1A2 (**i**) and MER52D (**j**) copies in macaque (left) and marmoset (right).*

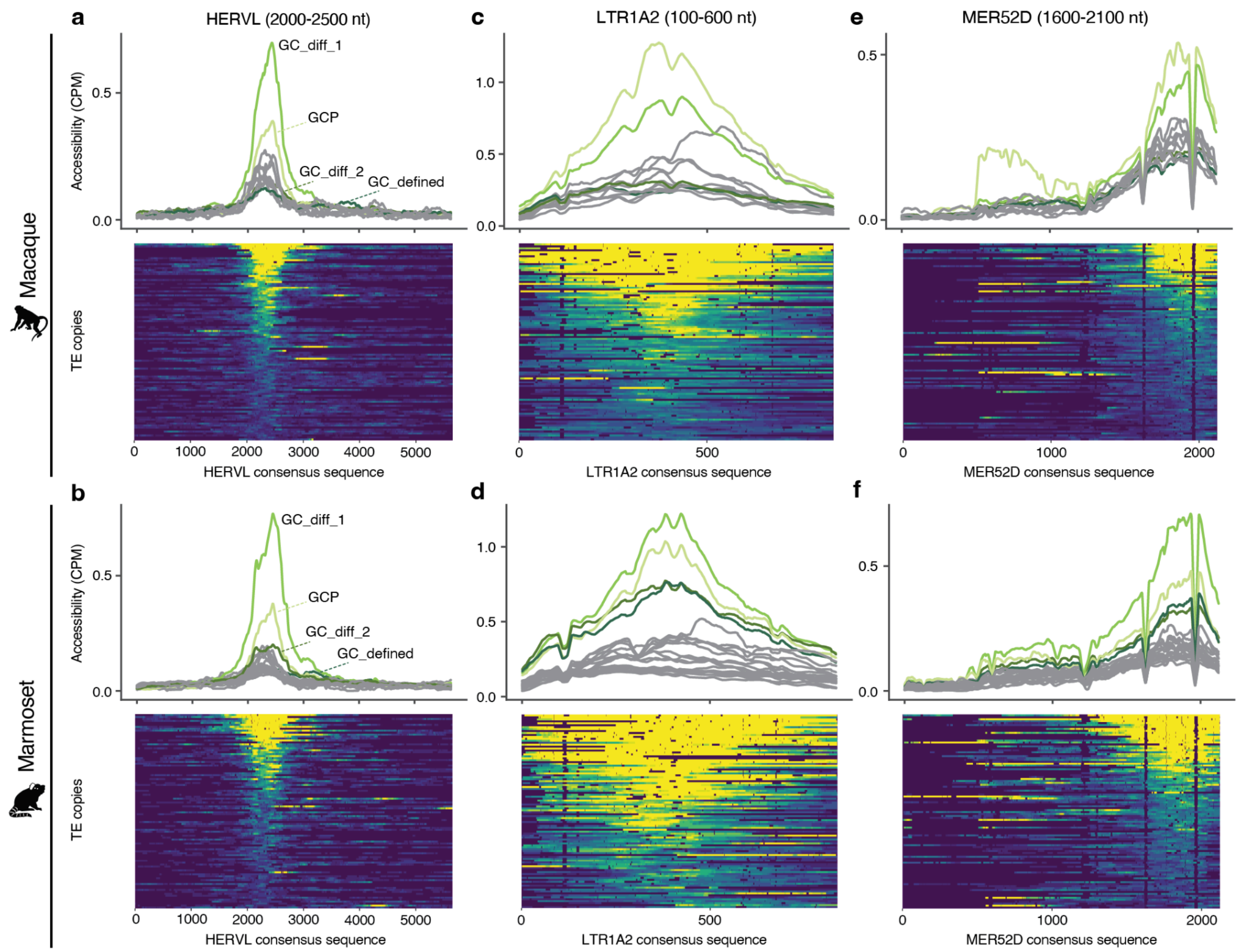

Supplementary Fig. 18 | Accessibility patterns of HERVL, LTR1A2, and MER52D across cerebellar cell types in macaque and marmoset. a–f, *Top: Mean chromatin accessibility profiles of the 30 most accessible HERVL (**a, b**), LTR1A2 (**c, d**), and MER52D (**e, f**) copies across cell groups in macaque (**a, c, e**) and marmoset (**b, d, f**) cerebellar development, aligned to the respective consensus sequence. Bottom: Chromatin accessibility patterns of the 100 most accessible HERVL (**a, b**), LTR1A2 (**c, d**), and MER52D (**e, f**) copies in differentiating granule cells (GC_diff_1) in macaque (**a, c, e**) and marmoset (**b, d, f**), aligned to the consensus sequence.*

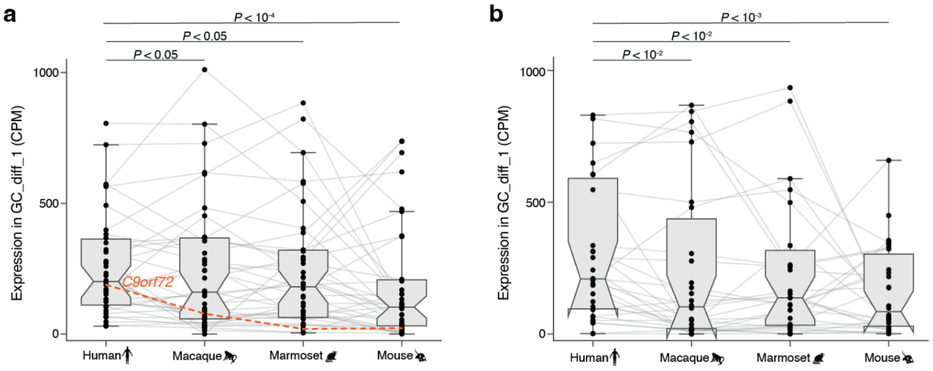

Supplementary Fig. 20 | Human-specific LTR1A2 and MER52D copies contribute to human-specific gene expression. a, b, *Expression levels of 1:1 orthologous genes located near LTR1A2 (**a**) and MER52D (**b**) copies that are accessible in differentiating granule cells (GC_diff_1) in human.*

MINOR POINTS

- The method is included somewhat by reference in this manuscript. Probably spelling out aspects of the training and evaluation needs to be more clearly explained.

We agree and have now added a methods section titled "DeepCeREvo training and architecture" that describes the key aspects of the model architecture, training strategy, and how it was applied in the current study. The added text reads as follows:

> **[Page 18, Line 667]** *DeepCeREvo is a deep learning model previously developed to predict CRE assignment to programs based on DNA sequence[39]. The model is a multiclass multilabel classifier with a hybrid convolutional and recurrent neural network architecture. The model takes 500 bp one-hot encoded DNA sequences as input, which are processed through: (i) a convolutional layer with 512 kernels (size 24), of which 285 were initialized with JASPAR 2020 motifs; (ii) a max-pooling layer; (iii) a time-distributed dense layer combined with a bidirectional LSTM layer (256 neurons); and (iv) final dense layers with sigmoid activation to predict probabilities for each program.*
>
> *The original model was trained on a mixed human-mouse dataset containing highly variable CREs assigned to programs, supplemented with putatively inactive intergenic regions and lowly variable CREs, using an 80:10:10 training-validation-test split. Training employed data augmentation via sliding windows (500 bp windows, 50 bp stride) and was performed using the Adam optimizer with binary crossentropy loss. Model performance was evaluated using auROC and auPR metrics. In this study, we applied the trained DeepCeREvo model to systematically assess the regulatory potential of ancestral TE sequences (described in the section 'Assessing regulatory potential of TE subfamilies').*

- Proximity of HERVL copies needs a permutation matched by distance control.

We thank the reviewer for this important suggestion. To address the concern that the observed proximity between accessible HERVL copies and cell-type-specific cCREs might be confounded by distance to transcription start sites (TSS), we performed linear regression analysis controlling for TSS distance and other genomic features.

The analysis confirmed that accessible HERVL copies remain significantly closer to cCREs even after controlling for distance to TSS, GC content, element length, sequence divergence, and DeepCeREvo prediction score (β = -0.52, 95% CI: -0.73 to -0.32, $P < 10^{-6}$). This corresponds to a 48% reduction in distance for accessible copies compared to inaccessible ones. We have added this result to the manuscript:

> **[Page 11, Line 384]** *Accessible HERVL copies are located significantly closer to non-TE-derived cell-type-specific cCREs (median 64 kb versus 221–234 kb; $P < 10^{-4}$, Mann–Whitney U test) (Fig. 5i), with the strongest enrichment within 50 kb ($P < 10^{-5}$, Mann-Whitney U test) (Fig. 5j). This proximity effect persists after controlling for genomic and sequence features including distance to TSS (β = -0.52, $P < 10^{-6}$, linear regression).*

- Missing confidence intervals on some of the reporting.

We have now added confidence intervals to plots where they improve interpretability. Specifically, we added confidence intervals to Fig. 3a, 3d, 4b, 5e, 5f, and 5j.

In Figs. 3a, 3d, and 4b, we performed bootstrapping analysis to estimate confidence intervals. To avoid overcrowding of shades, we added confidence intervals only to the cell lineages highlighted within the panels. These updated figures now more clearly show that the mean signals reflect broad contributions across copies, rather than being driven by a few outlier loci with disproportionately high accessibility.

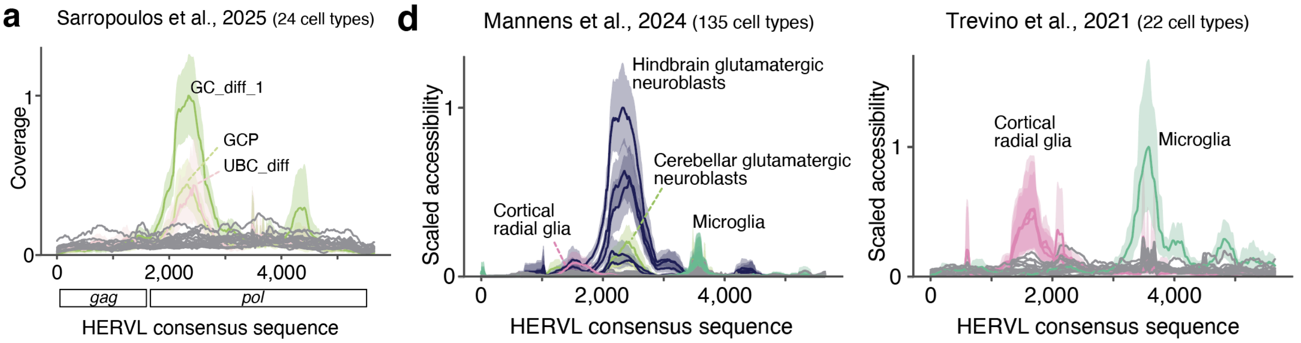

***Fig. 3a, d** | **a**, Mean chromatin accessibility profiles of the 50 most accessible HERVL copies across 24 cell groups in human cerebellar development, aligned to the consensus sequence, with the 95% confidence intervals based on bootstrapping analysis for the highlighted cell types. **d**, Mean accessibility profiles of the 50 most accessible HERVL copies across diverse cell types from different brain regions at various developmental stages (data from Mannens et al., 2024[61] and Trevino et al., 2021[73]), aligned to the HERVL consensus sequence, with the 95% confidence intervals based on bootstrapping analysis for the highlighted cell lineages.*

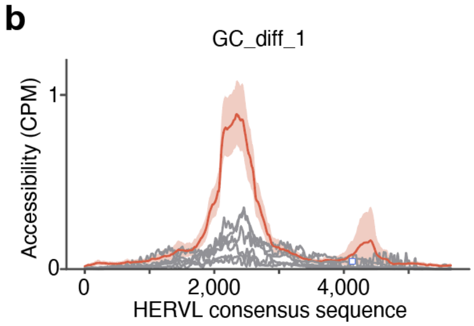

Fig. 4b | ***b,*** *Mean accessibility profile of the 50 most accessible copies of internal regions from HERVL and other transposon subfamilies within the ERVL family, with the 95% confidence intervals based on bootstrapping analysis for HERVL.*

Fig. 5e and 5f now more clearly show that sequences at positions corresponding to ancestral TF binding motif instances are recurrently used across copies and significantly more likely to be preserved in accessible copies.

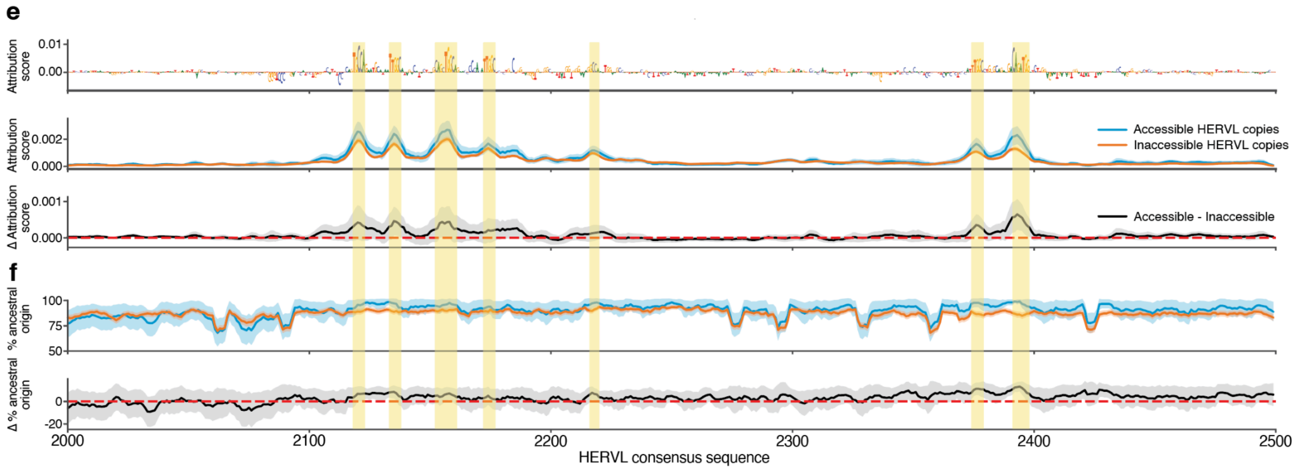

Fig. 5e, f | ***e,*** *DeepExplainer attribution profile of the HERVL consensus sequence (top); mean DeepExplainer attribution profiles of accessible and inaccessible HERVL copies (middle) and the difference between them (bottom), with the 95% confidence interval shown. TF binding motif instances in the consensus sequence are highlighted.* ***f,*** *Percentage of nucleotides identical to the consensus sequence in accessible versus inaccessible HERVL copies (top), and the difference between them (bottom), with the 95% confidence interval shown.*

Additionally, Fig. 5j better depicts the uncertainty of our measure, owing to the relatively low number of accessible copies with DeepCeREvo prediction scores larger than 0.5 (n = 35). We also note that, upon revision, we identified that Fisher's exact test had been applied incorrectly in Fig. 5j. We have corrected this to a Mann-Whitney U test, which is appropriate for comparing the continuous distributions in this analysis. The *P* values are slightly different from those reported in the original manuscript, but the conclusions remain unchanged.

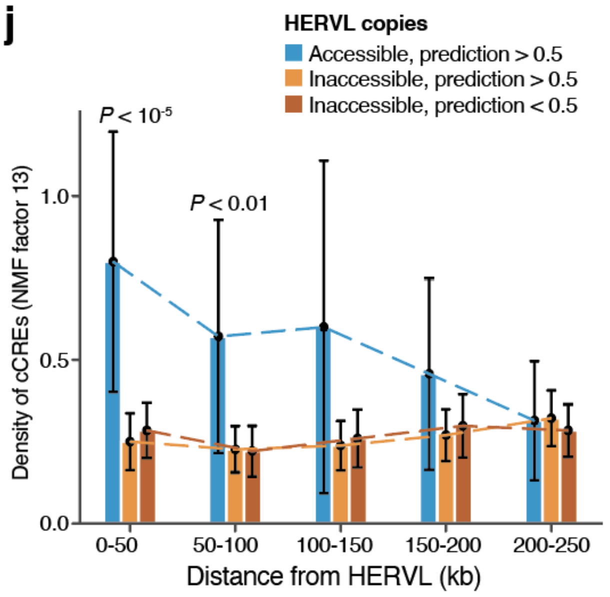

Fig. 5j | j, *Density of non-TE-derived cCREs around HERVL copies, with the 95% confidence interval shown.*

Reviewer: 3

Yamada et al. conducted a comprehensive analysis of the relationship between transposable elements (TEs) and cis-regulatory elements (CREs) during cerebellar development, focusing primarily on primates and including comparative analyses in mouse. Their study systematically examined how TEs contribute to the acquisition of cell-lineage-specific CREs and to regulatory innovation during human evolution.
In particular, they demonstrated that distinct sequence motifs within HERVL elements contribute to the emergence of transcription-factor (TF) binding sites in different cell lineages. The TF-binding motifs identified in the 2,000–2,500 nt region of HERVL are derived from proto-motifs present in ancestral sequences and were functionally reinforced through human-specific substitutions that enhance enhancer activity. The authors further showed that the regulatory activity of HERVL-derived CREs depends not only on the presence of TF-binding motifs but also on the density of neighboring non-TE-derived CREs, highlighting the role of local chromatin context in enabling HERVL activation. Finally, they demonstrated that the sets of active HERVL copies and their target genes differ across species, suggesting that HERVL insertions have contributed to the evolution of species- and cell-type-specific gene-expression programs in primates.
This study provides important insights into how TEs have been co-opted as cell-type-specific CREs and how these events have influenced gene-expression patterns in the primate cerebellum. It represents a significant contribution to our understanding of the interplay between TEs, genome evolution, and species diversification.
Overall, the manuscript is clearly written and presents a coherent and well-supported set of analyses. Addressing the points raised below would further refine the work and enhance its impact.

We thank the reviewer for the positive evaluation of our work and the constructive feedback. We have carefully addressed each point raised, and we believe the revisions have strengthened the manuscript.

MAJOR COMMENTS

1) The section "TE contributions to species- and cell-type-specific cis-regulatory elements in mammalian cerebellum development" compellingly shows that TE-derived elements are enriched among species-specific cCREs, with particularly strong contributions in primates (~60%) compared to mouse (~34%). This supports the general idea that TEs contribute to lineage-specific regulatory innovation. However, it remains unclear whether the authors interpret this as a broadly mammalian trend or as a phenomenon primarily driven by primate evolution. In primates, TE-derived cCREs are clearly enriched relative to the genomic TE background, whereas in mouse this proportion may be lower or even below the genomic expectation.

Moreover, the evidence for species-specific TE contributions in mouse appears comparatively limited throughout the study—largely serving as a reference or phylogenetic comparison rather than providing parallel mechanistic insights. If TE-driven co-option in mouse is indeed weaker or not statistically enriched, the current framing and title ("mammalian cerebellum development") might

slightly overstate the generality of the findings. Conversely, if the lower proportion in mouse reflects faster TE sequence decay or longer divergence time rather than a genuine biological difference, clarifying this point would substantially strengthen the interpretation.

I would encourage the authors to explicitly report TE enrichment values relative to genomic coverage in each species and to clarify whether the conclusions are intended to describe mammalian-wide mechanisms or primarily primate-specific innovations. Depending on this clarification, the title might be adjusted to more accurately reflect the scope of the presented evidence.

We thank the reviewer for this thoughtful observation regarding the interpretation of TE contributions across mammalian species. We have revised the manuscript to address these concerns. Specifically, we have:

1. implemented rigorous logistic regression analyses for all three species, showing consistent patterns across mammals whereby species-specific elements are enriched for overlap with TEs after controlling for peak type and GC content;
2. clarified in the Results section that technical and evolutionary factors—faster substitution rates, broader phylogenetic classification, and different divergence timescales—likely contribute to lower TE proportions in mouse-specific cCREs; and
3. changed the title to "Gene regulatory innovations from transposable elements in primate cerebellum development" to accurately reflect that primates are our primary focus.

The detailed changes are described below.

First, regarding whether TE-mediated regulatory innovation represents a broadly mammalian trend, we can confirm that this is indeed the case based on our statistical analyses. We have added logistic regression analyses modeling TE overlap within cCREs as a function of conservation level, controlling for peak type and GC content, separately for human, marmoset, and mouse. The revised manuscript reads as follows:

> **[Page 3, Line 109]** *Using logistic regression controlling for peak type and GC content, lineage-specific cCREs showed strong TE enrichment relative to unclassified cCREs: human-specific cCREs had 62% TE overlap (OR = 6.25, $P < 10^{-15}$) and primate-specific cCREs had 29% TE overlap (OR = 1.82, $P < 10^{-15}$) (Fig. 1c; Supplementary Table 1). Conversely, mammalian-conserved cCREs showed only 5% TE overlap (OR = 0.24, $P < 10^{-15}$), though the contributions of older TEs to conserved cCREs may be underestimated due to difficulty of annotating highly degenerated TEs[42]. This pattern of TE enrichment in lineage-specific elements generalizes to marmoset and mouse cCREs. Specifically, 63% of marmoset-specific cCREs (OR = 5.86, $P < 10^{-15}$) and 34% of mouse-specific cCREs (OR = 5.89, $P < 10^{-15}$) overlap with TEs, whereas fewer than 5% of cCREs with conserved accessibility across species overlap with TEs in both marmoset and mouse (Supplementary Fig. 1a,b; Supplementary Table 1).*

Second, regarding the lower absolute TE proportion in mouse (~34%) compared to human or marmoset (~60%), this likely reflects faster TE sequence decay and longer sequence divergence time rather than a genuine biological difference. Specifically, (1) the mouse genome has an approximately two-fold higher substitution rate per unit time than primate genomes, leading to faster TE sequence decay and reduced annotation sensitivity for ancient insertions; and (2) due to sparser phylogenetic sampling in Glires compared to primates, our "mouse-specific" category necessarily encompasses elements ranging from truly mouse-specific up to broader Glires-specific (rodent + lagomorph) elements—and since older, more conserved elements show lower TE enrichment (as shown in manuscript Fig. 1c and Supplementary Fig. 1), their inclusion reduces the overall proportion. These factors complicate direct cross-species comparisons of absolute proportions, whereas the within-species enrichment patterns remain consistent. We have added the following clarification in the Results:

> **[Page 4, Line 119]** *The overall lower contribution of TEs in mouse cCREs likely reflects a lower genomic TE background in the mouse genome, faster TE sequence decay due to higher substitution rate in this lineage, and longer divergence time since the split from the common ancestor of the lineage encompassing primates and glires.*

Third, regarding the scope and framing of the manuscript, we agree with the reviewer that our study focuses primarily on human and other primates, with mouse serving as an evolutionary outgroup. We have changed the title to "Gene regulatory innovations from transposable elements in primate cerebellum development" and revised the framing throughout the manuscript to accurately reflect this scope while acknowledging that the core mechanisms appear to be broadly mammalian.

2) In Figure 1f, several TE subfamilies—notably from the ERV1 class but also including certain LINE, SINE, and DNA transposon families—show enrichment across multiple NMF programs. This pattern appears to contrast with the notion that cell-type-specific cCREs are more frequently TE-derived. It would be helpful if the authors could clarify whether these shared enrichments reflect (i) recurrent co-option of distinct copies from the same TE subfamily in different cell types, (ii) the presence of broadly active promoter-like motifs within TEs, or (iii) background accessibility biases associated with ancient TE insertions. A short discussion of these possibilities would help reconcile the apparent overlap of TE enrichment across programs with the conclusion of cell-type-specific TE co-option.

We investigated this pattern in detail and found that it can be explained by three main reasons:

1. High enrichment signals in low-abundance TE subfamilies (those with few overlapping cCREs) can be unreliable, as even a small number of additional overlaps substantially inflates enrichment scores.
2. Enrichment across multiple programs within the same cell lineage reflects the correlated accessibility of TE copies across related cell types.

3. Enrichment across programs from distinct cell lineages can arise from distinct ancestral sequence features within the same TE subfamily that drive preferential co-option in different lineages.

To clarify these points, we have modified existing analyses, added new ones, and revised our manuscript accordingly. The detailed changes are described below.

First, we noticed that some TE subfamilies showing high enrichment signals across multiple programs have only a small number of overlaps with cCREs. For these low-abundance subfamilies, even a few additional overlaps can substantially inflate enrichment scores, making them unreliable. To address this, we have added an additional filtering step requiring TE subfamilies with high enrichment scores ($\log_2$ enrichment > 2) to have more than 10 overlaps with program-specific cCREs. Since we already focus on TE subfamilies with at least 10 overlaps in the in silico screening for ancestral sequence-driven co-option, this additional criterion does not affect any of the downstream analyses. Accordingly, we have updated Fig. 1g (Fig. 1f in the original manuscript) and added a paragraph to the Methods section as follows:

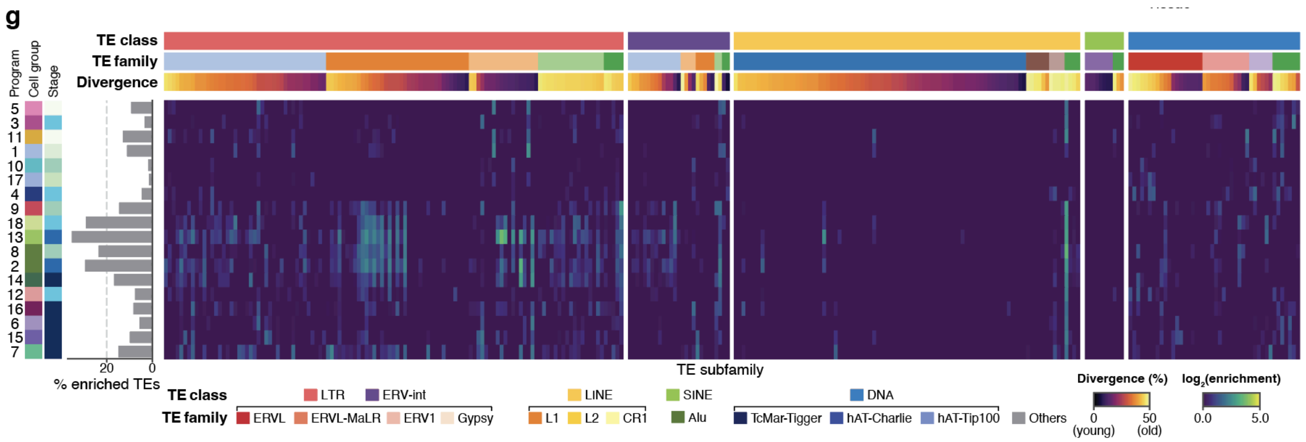

Fig. 1g | *Subfamily-specific TE enrichment within program-specific cCREs. (Left) bar plot indicates the proportion of each enriched TE subfamily contributing to TE-derived cCREs. Note that highlights for LTR1A2 and MamRTE1 are only added in this response.*

[Page 18, Line 646] *When analysing TE subfamily enrichment across program-specific cCREs, we first filtered out TE subfamilies with no more than ten cCREs across programs, retaining 322 of 902 subfamilies. After computing enrichment scores, we noticed that some TE subfamilies showing high enrichment signals have only a small number of overlaps with cCREs. For these low-abundance subfamilies, even a few overlaps can substantially inflate enrichment scores, making them unreliable. To address this, we implemented an additional filtering step requiring TE subfamilies with high enrichment scores ($\log_2$ enrichment > 2) to have more than 10 overlaps with program-specific cCREs. This filtering step excluded 31 TE subfamilies, resulting in 291 TE subfamilies for enrichment analysis across programs.*

Second, we observe cell lineage-specific patterns of TE enrichment across multiple programs. This reflects the correlated accessibility of individual TE copies within related cell lineages—when a TE subfamily is co-opted in one lineage-specific program, its copies tend to be accessible in related programs as well. To clarify this, we have added a pairwise correlation analysis of log_2 enrichment scores across programs and modified our manuscript accordingly, adding Supplementary Fig. 3:

> **[Page 5, Line 171]** *These lineage-specific patterns are reflected in the correlated enrichment of TE subfamilies across related programs: pairwise correlation analysis of TE enrichment scores revealed clustering within the granule cell lineage and among neural progenitors and neuroblasts (Supplementary Fig. 3), suggesting that individual TE subfamilies are frequently co-opted across multiple programs within related cell groups.*

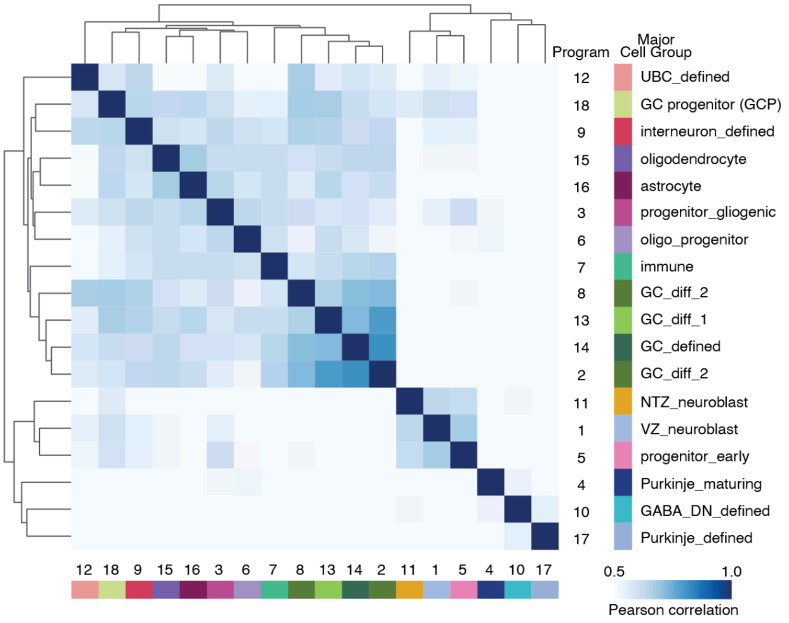

Supplementary Fig. 3 | Pairwise correlation analysis of TE subfamily enrichment in program-specific cCREs. *Pairwise correlation matrix of log_2 enrichment of TE overlap with cCREs specific to individual programs. Rows and columns are ordered by hierarchical clustering of the correlation matrix.*

Third, enrichment of a TE subfamily across programs from different cell lineages can reflect preferential co-option driven by distinct sequence features within the same subfamily. For instance, LTR1A2 shows enrichment not only in the granule cell lineage (programs 18, 13, 8, 2, 14) but also in the oligodendrocyte lineage (programs 6 and 15). We discuss this subfamily in detail in the manuscript and show that (1) increased accessibility in these different cell states maps to distinct positions within the consensus sequence (Supplementary Fig. 6b) and (2) both patterns are associated with high regulatory potential at the respective positions in the ancestral sequence (Supplementary Fig. 8c,d). Furthermore, at the copy level, accessible copies are largely distinct between the granule cell and oligodendrocyte lineages, and even within the same lineage, the same copies are not always accessible across related programs (Supplementary Fig. 9). Accordingly, we have added LTR1A2 analyses to our revised manuscript, complementing the original HERVL analysis, and added Supplementary Figs. 6b, 8c,d and 9 with the following text:

[Page 8, Line 279] *Similarly, LTR1A2 shows enriched accessibility not only in rhombic lip-derived neuroblasts but also in oligodendrocyte precursor cells (Supplementary Fig. 6a,b), and its ancestral sequence exhibits high regulatory potential in the corresponding program and contains binding motif instances for SOX TFs, which are known to be important for oligodendrocyte differentiation[80] (Supplementary Fig. 8c,d).*

[Page 8, Line 297] *Similar patterns were observed in LTR1A2, with distinct clusters identified for microglia, oligodendrocyte precursors, and rhombic lip-derived neuroblasts, and finer heterogeneity observed within the rhombic lip-derived neuroblasts (Supplementary Fig. 9). Collectively, these results illustrate the flexibility of TE co-option: the same TE subfamily can be co-opted by multiple cell states in the same cell lineage sharing regulatory programs, or by distinct cell states through different motif instances within the ancestral sequence, with variation also observed at the level of individual genomic copies.*

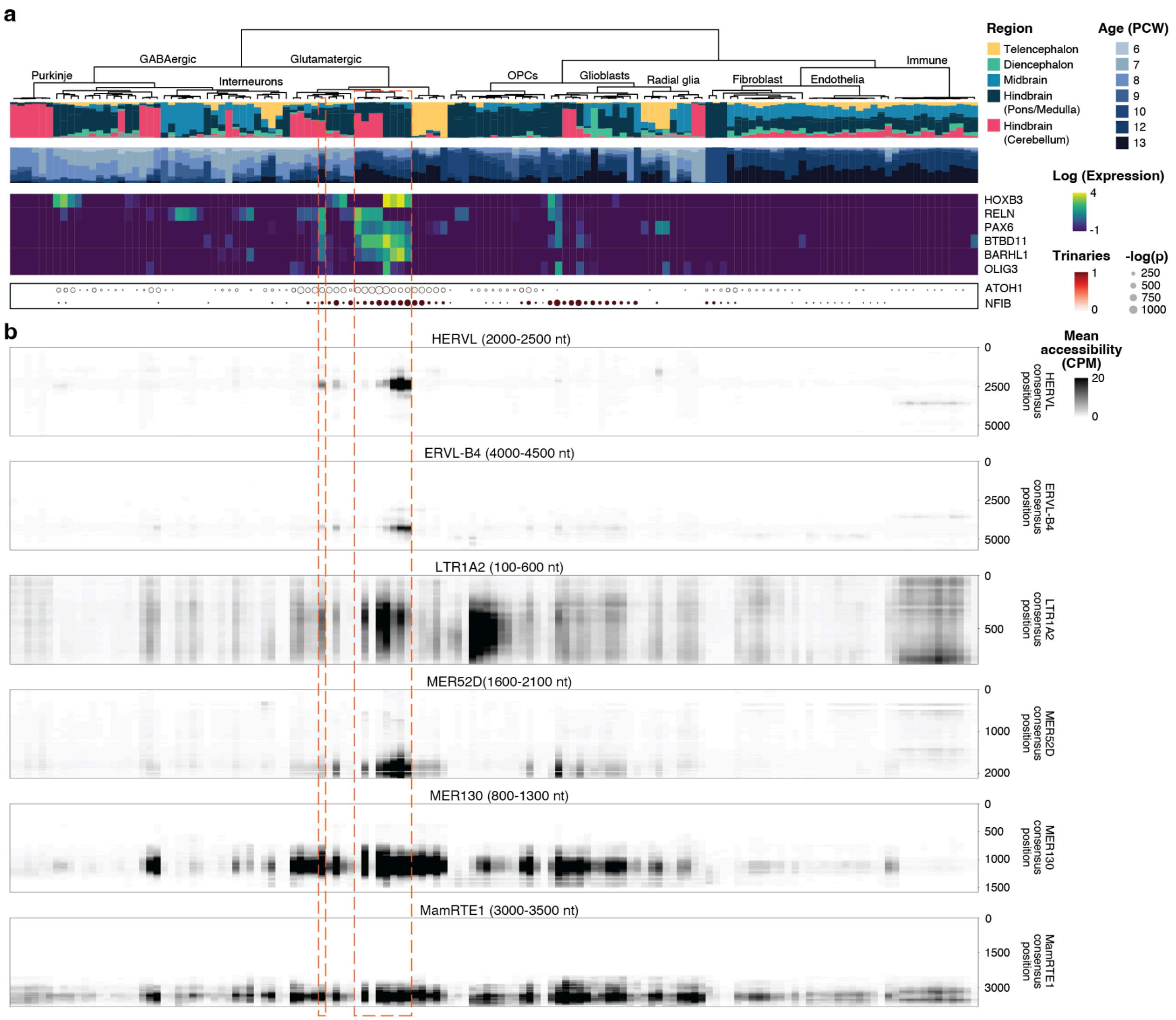

Supplementary Fig. 6 a,b | Hindbrain glutamatergic neuroblast-specific accessibility of the screened TE subfamilies. a, *Top to bottom: hierarchical clustering dendrogram showing relationships between cell types based on highly variable cCREs; anatomical region distribution showing the proportion of each cell type across brain regions; developmental age distribution showing temporal profiles of each cell type; expression heatmap of selected marker genes across cell types; TF binding motif enrichment analysis with dot size representing statistical significance (P-values) and color intensity representing expression levels of the corresponding TFs (trinarization score represents a probabilistic measure of gene expression ranging from 0-1). Highlighted cell types show HERVL accessibility (Fig. 3d). Data from Mannens et al., 2024[2] reanalyzed.* ***b****, Mean accessibility profiles of the 50 most accessible copies of each of the six TE subfamilies identified as specifically co-opted in the granule cell lineage (manuscript Supplementary Fig. 4), across diverse cell types from different brain regions at various developmental stages (data from Mannens et al., 2024[2]), aligned to the consensus sequence of the respective TE subfamily.*

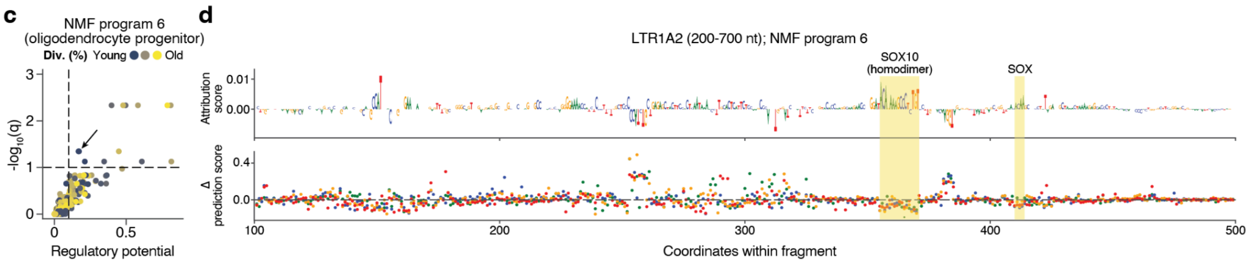

Supplementary Fig. 8 | Sequence features explaining increased accessibility in distinct cell types. c, *Regulatory potential scores and associated Q-values in oligodendrocyte progenitors (program 6) for all TE fragments.* ***d,*** *DeepExplainer attribution profiles in oligodendrocyte progenitors for LTR1A2 at the 200–700 nt position, which is indicated by an arrow in (****c****).*

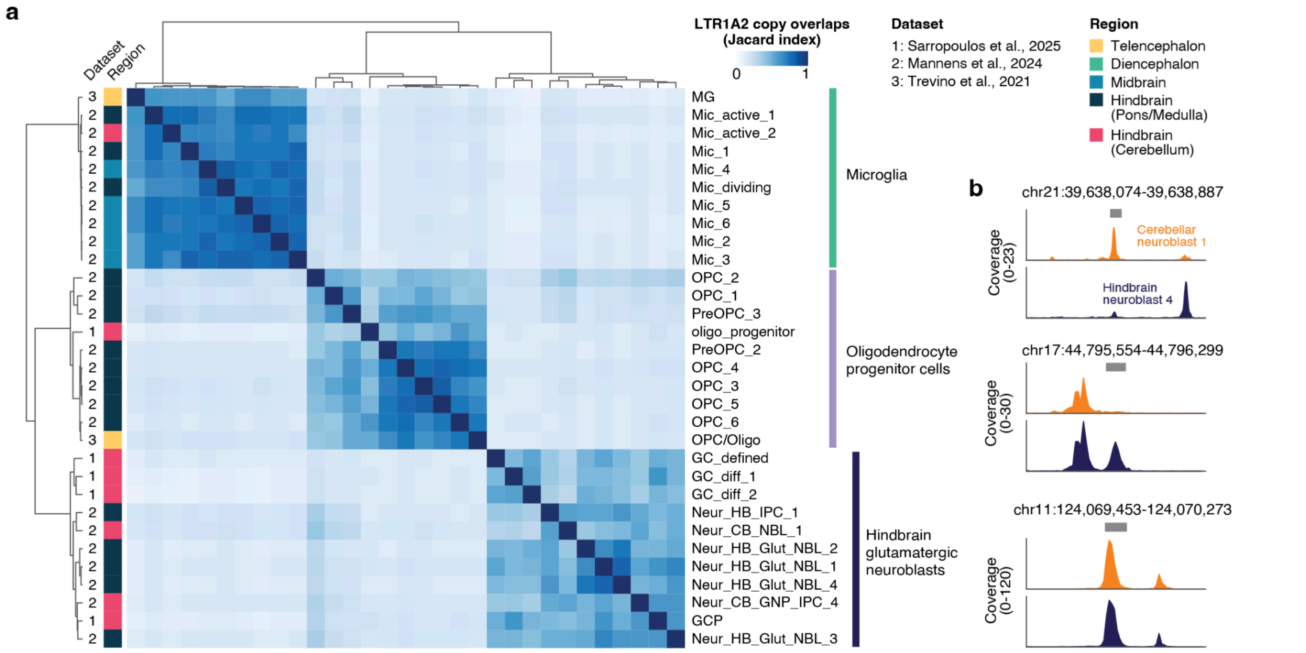

Supplementary Fig. 9 | Heterogeneity of LTR1A2 accessibility at the copy level. a, *Pairwise Jaccard similarity index matrix of the 50 most accessible LTR1A2 copies across cell types.* ***b,*** *Examples of LTR1A2-overlapping cCREs specific to cerebellar neuroblasts 1 (Neur_CB_NBL_1; top), hindbrain*

glutamatergic neuroblasts 4 (Neur_HB_Glut_NBL_4; middle), or shared between these two cell types (bottom). Annotated LTR1A2 copies are shown as gray bars.

3) The authors conclude that pre-existing cis-regulatory motifs in ancestral TE sequences facilitate their preferential co-option as CREs. This interpretation is compelling, but it assumes that the relevant TF motifs were already present in ancestral states. However, convergent emergence of TF-binding motifs after insertion has also been documented for several TE families. It would therefore be informative to test whether ancestral TE fragments that already contained motifs show stronger cCRE enrichment than motif-lacking fragments, to distinguish between pre-adaptive and convergent modes of regulatory evolution.

We thank the reviewer for this thoughtful comment regarding the distinction between pre-adaptive and convergent modes of regulatory evolution. We believe our data provide multiple lines of evidence supporting the pre-adaptive model for HERVL:

1. No convergently acquired sequence features with high attribution across accessible HERVL copies: Our original analysis showed that many accessible HERVL copies retain the same TF binding site repertoire as the ancestral sequence (Supplementary Fig. 11). We extended this analysis by computing attribution scores for all accessible and inaccessible HERVL copies and mapping these scores to ancestral coordinates. This revealed that sequence features assigned high attribution in accessible HERVL copies predominantly overlap with TF binding sites in the ancestral sequence. Together, these findings indicate that retention of ancestral TF binding sites, rather than convergent evolution of novel motifs, is the primary determinant of regulatory activity.

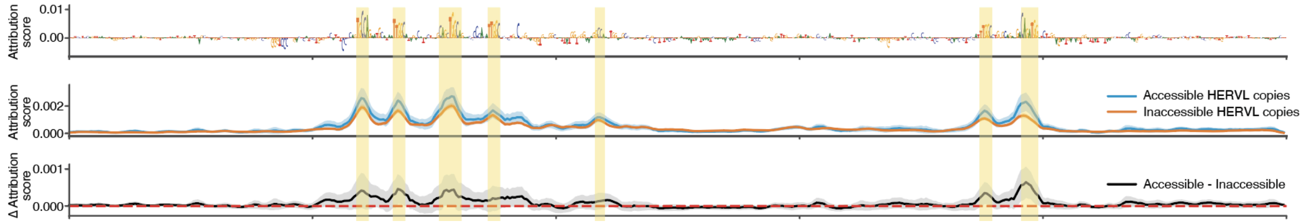

***Fig. 5e** | DeepExplainer attribution profile of the HERVL consensus sequence (top); mean DeepExplainer attribution profiles of accessible and inaccessible HERVL copies (middle) and the difference between them (bottom), with the 95% confidence interval shown. TF binding motif instances in the consensus sequence are highlighted.*

2. Accessible copies have lower scores than the ancestral consensus: Our DeepCeREvo analysis revealed that accessible HERVL copies have a median prediction score (0.72) that is comparable to that of the reconstructed ancestral consensus sequence (0.74) (manuscript Fig. 5d). This finding is inconsistent with convergent evolution toward optimized regulatory sequences and instead indicates that regulatory activity does not require sequence improvement beyond the ancestral state. Accessible copies simply need to preserve sufficient ancestral motif content, not evolve enhanced versions.

3. Functional validation of the consensus sequence: We showed that the reconstructed ancestral HERVL sequence itself possesses enhancer activity in reporter assays (manuscript Fig. 4d), confirming that regulatory potential was present in the ancestral state prior to genomic insertion of individual copies.

Collectively, these analyses strongly suggest that the regulatory activity of HERVL elements represents pre-adaptive co-option of ancestral motifs, with individual copy accessibility determined primarily by the degree of preservation of these pre-existing motifs rather than by convergent evolution of new regulatory sequences. We have revised the manuscript and modified Fig. 5e to make this interpretation more explicit as follows:

> **[Page 10, Line 362]** *The median score of accessible copies (0.72) was comparable to that of the consensus sequence (0.74), suggesting that HERVL accessibility reflects preservation of pre-adaptive sequences from the ancestral element rather than convergent acquisition of regulatory motif instances through mutations after insertion. Consistently, sequences at positions corresponding to ancestral TF binding motif instances are recurrently used across copies and significantly more likely to be preserved in accessible copies (Fig. 5e,f; Supplementary Fig. 11; $P < 10^{-13}$, linear regression), with no consistently acquired high-attribution positions beyond ancestral motifs. Together with the luciferase assay results (Fig. 4d), these findings indicate that HERVL harbored "ready-to-use" pre-adaptive regulatory sequences at the time of insertion.*

Notably, not all subfamilies identified in our screening follow the HERVL pattern of "ready-to-use" pre-adaptive sequences. Using LTR1A2 as an example, we have added Supplementary Figs. 15 and 16 with the following text:

> **[Page 11, Line 413]** *Notably, not all subfamilies identified in our screening follow the HERVL pattern of "ready-to-use" pre-adaptive sequences. Comparison of DeepCeREvo prediction scores between accessible copies and the ancestral sequence revealed that extant LTR1A2 copies frequently possess higher prediction scores than the ancestral sequence (Supplementary Figs. 15, 16a). Attribution analysis further showed that accessible copies frequently acquired additional TF binding motif instances (Supplementary Fig. 16b). Moreover, some of these additional motif instances were acquired at orthologous positions across independent copies that are distributed throughout the LTR1A2 phylogenetic tree, confirming that the motifs arose independently through convergent evolution (Supplementary Fig. 16c). These convergently acquired motif sequences in extant copies can be explained by transition mutations, which are known to occur at higher rates than transversions[89], particularly at CpG sites via spontaneous deamination of methylated cytosine[90] (Supplementary Fig. 16d). These findings suggest that the ancestral sequences of LTR1A2 provided proto-regulatory potential that was subsequently augmented through convergent acquisition of new motif instances, likely facilitated by mutation bias acting on proto-motif sequences in the ancestral TE[20].*

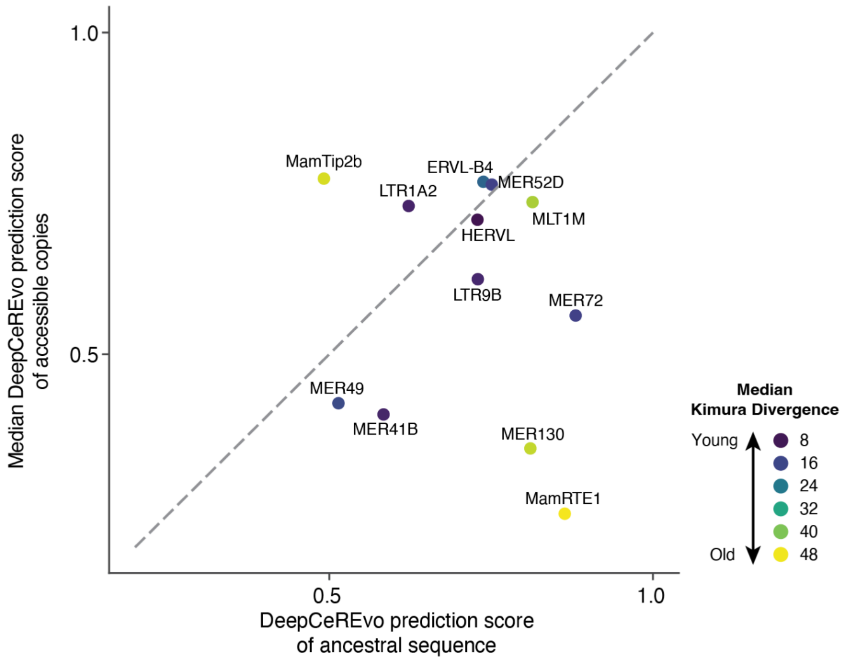

Supplementary Fig. 15 | Comparison of DeepCeREvo prediction scores between extant copies and ancestral sequences. *Scatter plot showing the DeepCeREvo score of the ancestral sequence (x-axis) against the median DeepCeREvo score of extant accessible copies (y-axis) for each TE subfamily. The dotted line indicates where ancestral and extant scores are equal; subfamilies falling above it have extant accessible copies with higher median DeepCeREvo scores than their ancestral sequence.*

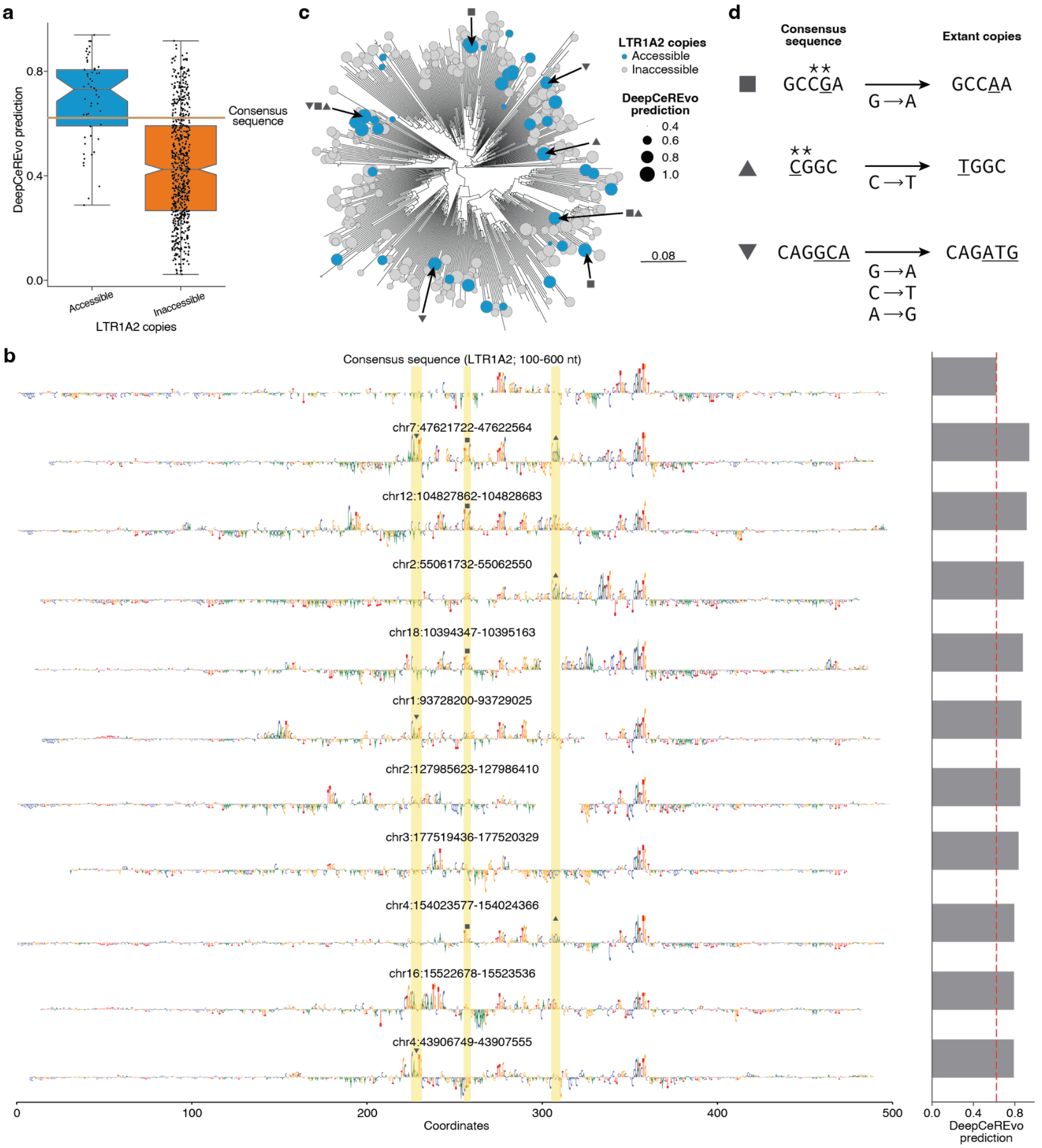

***Supplementary Fig. 16 | Convergent acquisition of TF binding motif instances in accessible LTR1A2 copies. a**, Comparison of DeepCeREvo prediction scores (program 13) between accessible and inaccessible LTR1A2 copies in differentiating granule cells, relative to the score of the consensus sequence. **b**, DeepExplainer attribution profiles (left) and DeepCeREvo prediction scores (right) for the LTR1A2 consensus sequence and the 10 LTR1A2 copies accessible in differentiating granule cells (GC_diff_1) with the highest prediction scores among those that preserved the ancestral TF binding motif instance. The DeepCeREvo prediction score of the consensus sequence is indicated for reference (dotted red line). TF binding motif*

instances absent from the consensus sequence but shared across different copies are highlighted and marked. ***c****, Phylogenetic relationship of LTR1A2 copies in the human genome, annotated with chromatin accessibility status in differentiating granule cells (GC_diff_1) and corresponding DeepCeREvo prediction scores (program 13). Accessible copies with TF binding motif instances marked in (****a****) are indicated by arrows and their corresponding marks.* ***d****, For each convergently acquired TF motif instance, the consensus sequence and the corresponding sequences in extant copies are shown, along with the substitution events underlying acquisition of each motif instance. Substituted nucleotides are underlined, and CpG dinucleotides are marked by asterisks.*

4) The authors propose that pre-existing TF binding motifs in ancestral TE sequences facilitate their co-option as cCREs. However, Figure 1g and related analyses show that older LINE and SINE subfamilies exhibit stronger enrichment in cCREs than younger ones. This pattern may be more consistent with post-insertion sequence evolution, whereby mutations accumulated over time created novel TF binding sites and regulatory potential, rather than preservation of ancestral motifs.
It would be helpful if the authors could clarify whether such classes represent cases of motif emergence through sequence drift, as opposed to ancestral motif retention, and how this distinction fits within their overall model of TE-driven regulatory innovation.

We agree with the reviewer that the observed age-dependency of TE-cCRE overlap enrichment is more consistent with evolutionary processes over time—allowing inserted copies time to acquire regulatory function while non-functional copies degenerate—rather than preferential co-option due to pre-adaptive ancestral sequence features. To clarify this interpretation, we have modified the Results and Discussion sections as follows:

> **[Page 5, Line 179]** *Older TE subfamilies in LINE, SINE, and DNA classes show higher cCRE overlap than younger ones (Fig. 1h), likely reflecting more time for inserted copies to acquire regulatory function and for non-functional copies to degenerate. In contrast, younger LTR and ERV-int subfamilies are as enriched as older ones (Fig. 1h), suggesting these TE classes are more readily co-opted as regulatory elements, in line with previous reports*[14,44,46].

> **[Page 14, Line 529]** *These patterns are consistent with evolutionary processes—mutation, natural selection, and genetic drift—where inserted TE copies serve as raw material for regulatory evolution similarly to other non-TE-derived sequences, rather than providing a "shortcut" via pre-adaptive sequences in their ancestral state. This model is further supported by older TE subfamilies from LINE, SINE, and DNA classes showing higher cCRE enrichment than younger ones, while the absence of this age dependence in TE subfamilies from LTR and ERV-int classes points instead to preferential co-option via pre-existing regulatory sequences. Together, these observations highlight that understanding TE contributions to regulatory evolution requires considering both evolutionary constraint and preferential co-option driven by ancestral sequence features.*

5) The attribution analysis in Fig. 2 suggests that specific TF binding motifs within ancestral TE fragments contribute to their predicted regulatory potential. However, attribution maps indicate sequence importance in silico and do not necessarily imply biological relevance. To support the conclusion that these motifs facilitate co-option, it would be valuable to assess whether the predicted motif sequences are evolutionarily conserved — either across genomic TE copies or between species. Demonstrating significant conservation of these motifs would strengthen the argument that they are functionally constrained and relevant for TE-derived CRE activity.

We agree that demonstrating evolutionary conservation would strengthen our argument that the predicted motifs are biologically relevant. We have now addressed this in two complementary ways: comparing preservation of ancestral sequence across genomic TE copies (within-genome) and across primate species (cross-species).

First, regarding the within-genome comparison, the original manuscript showed that accessible HERVL copies preserve ancestral sequences at positions corresponding to transcription factor binding motifs more than inaccessible copies, but lacked confidence intervals and formal statistical testing. We have now added 95% confidence intervals, showing that ancestral nucleotides are better preserved in accessible copies than in inaccessible ones (Fig. 5e,f). We also performed a linear regression to compare the proportion of ancestral sequence preservation between accessible and inaccessible HERVL copies at high-attribution positions (top 10%), while controlling for nucleotide identity. The results strongly support the biological relevance of the predicted motifs: accessible HERVL copies preserve ancestral sequences at high-attribution positions significantly more than inaccessible copies (coefficient = 0.03, $P < 10^{-7}$), after controlling for nucleotide composition. These patterns are also observed across other screened TE subfamilies (Supplementary Fig. 13). Together, this indicates that positions identified as important by the attribution model distinguish accessible from inaccessible TE copies, directly supporting the biological meaningfulness of our computational predictions. We have updated the Results and Methods sections, modified Fig. 5e,f, and added Supplementary Fig. 13 with Supplementary Table 6 as follows:

> **[Page 10, Line 366]** *Consistently, sequences at positions corresponding to ancestral TF binding motif instances are recurrently used across copies and significantly more likely to be preserved in accessible copies (Fig. 5e,f; Supplementary Fig. 11; $P < 10^{-13}$, linear regression), with no consistently acquired high-attribution positions beyond ancestral motifs.*
>
> **[Page 11, Line 408]** *The predictive power of DeepCeREvo scores reflects ancestral motif preservation: in young TE subfamilies, sequences at ancestral TF binding motif positions are recurrently used and significantly more preserved in accessible copies (Supplementary Fig. 13a–e; $P < 0.05$, linear regression), …*
>
> **[Page 24, Line 875]** *To test whether sequences at high-attribution sites are significantly more preserved in accessible copies relative to inaccessible copies, we performed a linear regression analysis. For each TE subfamily, we first computed per-position attribution scores on the ancestral consensus sequence using DeepExplainer with 100 shuffled background sequences. Positions were classified as high-attribution (attribution score > 90th percentile)*

or low-attribution (absolute attribution score < 90th percentile). Sequence conservation at each position was defined as the proportion of copies matching the ancestral nucleotide, and the conservation difference between accessible and inaccessible copies was computed as the outcome variable. We then fit an ordinary least squares regression model with the conservation difference as the dependent variable, a binary indicator for high- versus low-attribution positions as the primary predictor, and one-hot encoded nucleotide identity as a covariate to control for base composition effects. A significant positive coefficient for the high-attribution indicator would indicate that accessible copies preferentially preserve ancestral sequences at positions with high model attribution compared to positions with low attribution.

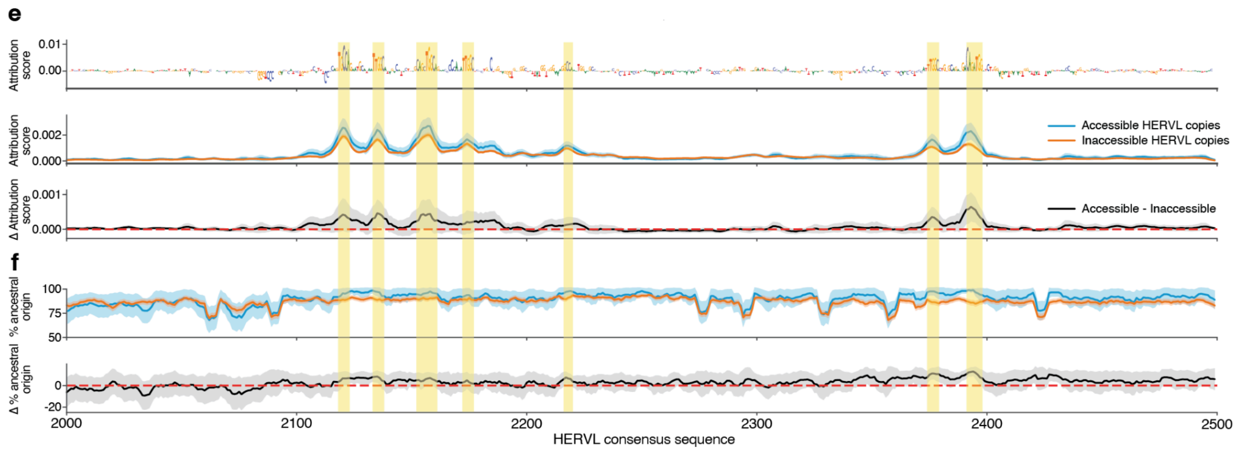

Fig. 5e, f* | *e, *DeepExplainer attribution profile of the HERVL consensus sequence (top); mean DeepExplainer attribution profiles of accessible and inaccessible HERVL copies (middle) and the difference between them (bottom), with the 95% confidence interval shown. TF binding motif instances in the consensus sequence are highlighted.* ***f***, *Percentage of nucleotides identical to the consensus sequence in accessible versus inaccessible HERVL copies (top), and the difference between them (bottom), with the 95% confidence interval shown.*

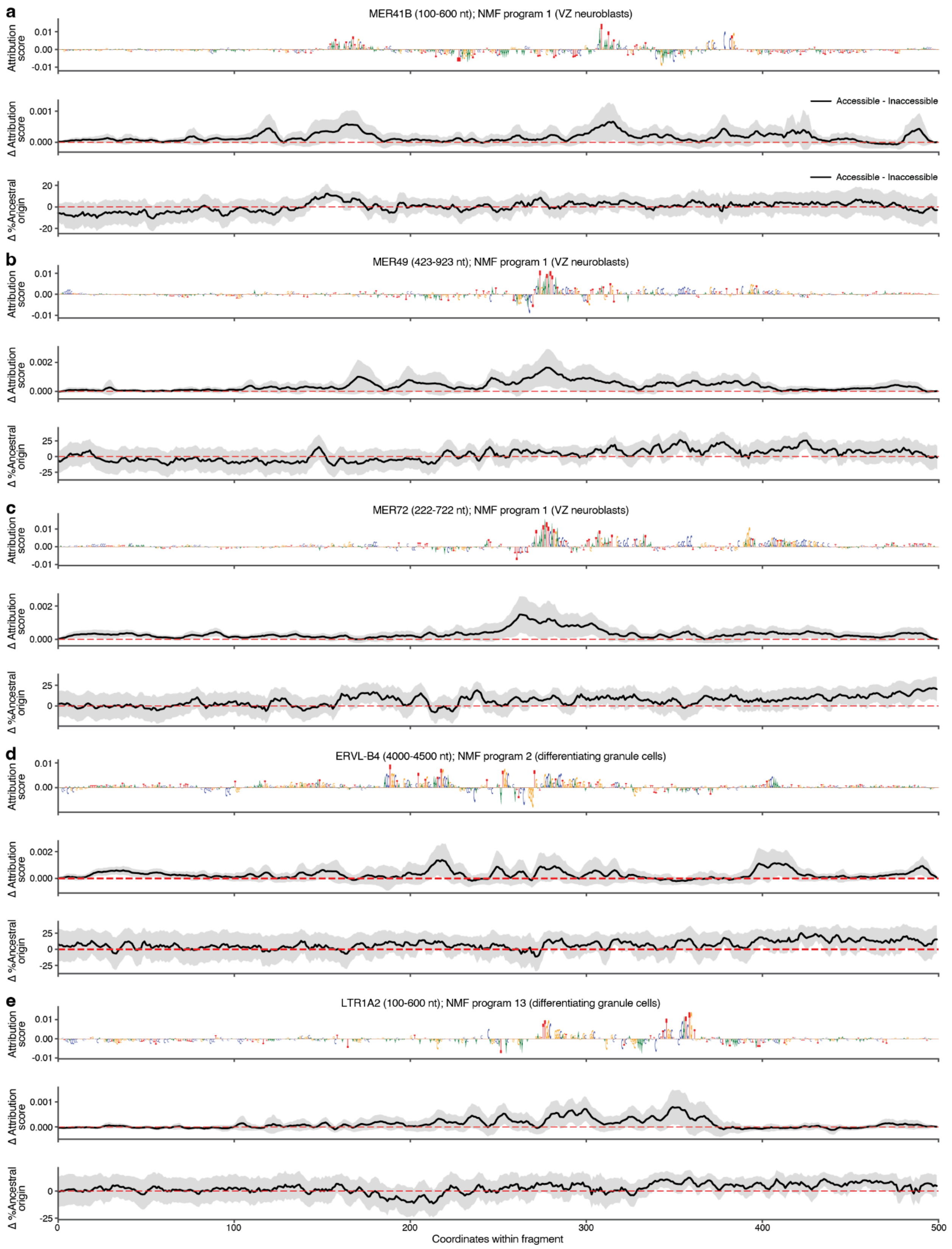

***Supplementary Fig. 13 | Recurrent usage of ancestral TF motif instances and preservation of ancestral nucleotides in accessible copies. a–e**. DeepExplainer attribution profile of the consensus sequence of the TE*

*subfamily (top). Difference in attribution profiles between accessible and inaccessible copies, aligned to the consensus sequence, with the 95% confidence interval shown (middle). Difference in the percentage of nucleotides identical to the consensus sequence between accessible and inaccessible copies, aligned to the consensus sequence (bottom). Plots are shown for MER41B (**a**), MER49 (**b**), MER72 (**c**), ERVL-B4 (**d**), and LTR1A2 (**e**).*

Second, to examine cross-species conservation, we computed phastCons conservation scores across 30 primate species for all HERVL copies and mapped these to the consensus sequence. However, interpreting these results is subject to important limitations. First, the sample size of accessible HERVL copies is small (~40). Second, HERVL insertions are relatively recent, with more than 80% of human HERVL copies absent in New World monkeys, limiting the evolutionary depth available for conservation analysis. Third, accessibility itself is highly lineage-specific (Fig. 6a), making it difficult to identify consistent conservation signals across species. Together, these factors make it challenging to definitively establish the presence or absence of purifying selection using cross-species data. With these caveats in mind, the phastCons values in accessible HERVL copies are not particularly high, reflecting the neutral-to-conserved scale (0–1) of the metric and the constraints noted above. Nevertheless, using linear regression we detected a statistically significant difference in conservation between accessible and inaccessible copies (coefficient = 0.018, $P < 10^{-3}$), though the effect size is modest and the 95% confidence intervals show substantial overlap (Reviewer Figure 3). We therefore consider the cross-species conservation evidence inconclusive but consistent with our attribution-based findings.

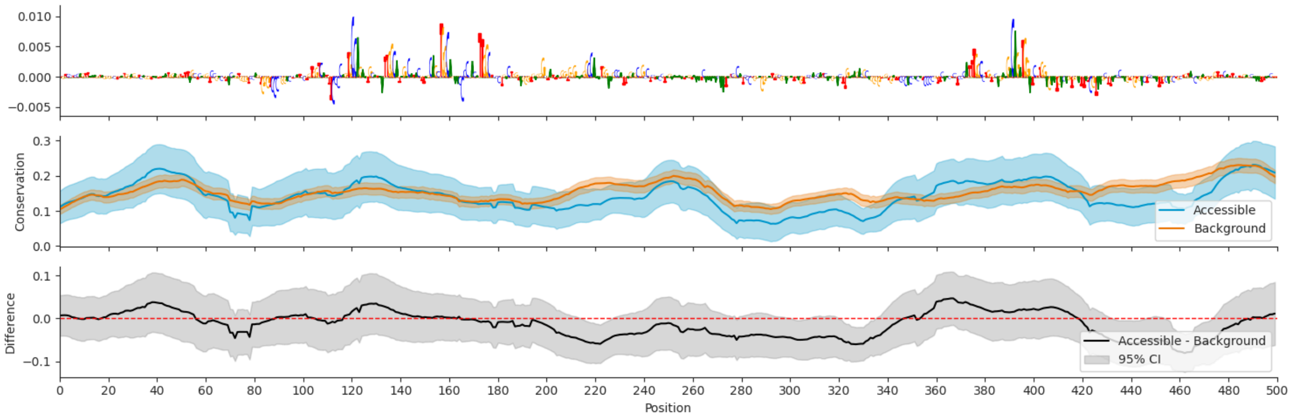

Reviewer Figure 3 | DeepExplainer attribution profile of the HERVL consensus sequence (top); mean phastCons scores from 30 primate species in accessible versus inaccessible HERVL copies (middle), and the difference between them (bottom), with the 95% confidence interval shown.

Importantly, in a new analysis of screened TE subfamilies whose insertion events predate the mammalian common ancestor, we find that sequences at ancestral TF binding motif positions exhibit stronger purifying selection across 447 mammalian genomes based on phyloP scores (Pollard et al., *Genome Res.*, 2010 [PMID: 19858363]; Zoonomia Consortium, *Nature*, 2020, [PMID: 33177664]; Kuderna et al., *Science*, 2023 [PMID: 37262161]) (Supplementary Fig. 14). This further supports the

biological meaningfulness of our computational predictions. We have added Supplementary Fig. 14 with Supplementary Table 6 and updated the Results section accordingly:

> **[Page 11, Line 408]** *The predictive power of DeepCeREvo scores reflects ancestral motif preservation: in young TE subfamilies, sequences at ancestral TF binding motif positions are recurrently used and significantly more preserved in accessible copies (Supplementary Fig. 13a–e; $P < 0.05$, linear regression), whereas in older subfamilies, these positions show significantly stronger purifying selection across 447 mammalian genomes[86–88] (Supplementary Fig. 14a–d, $P < 0.05$, linear regression) (Supplementary Table 6).*

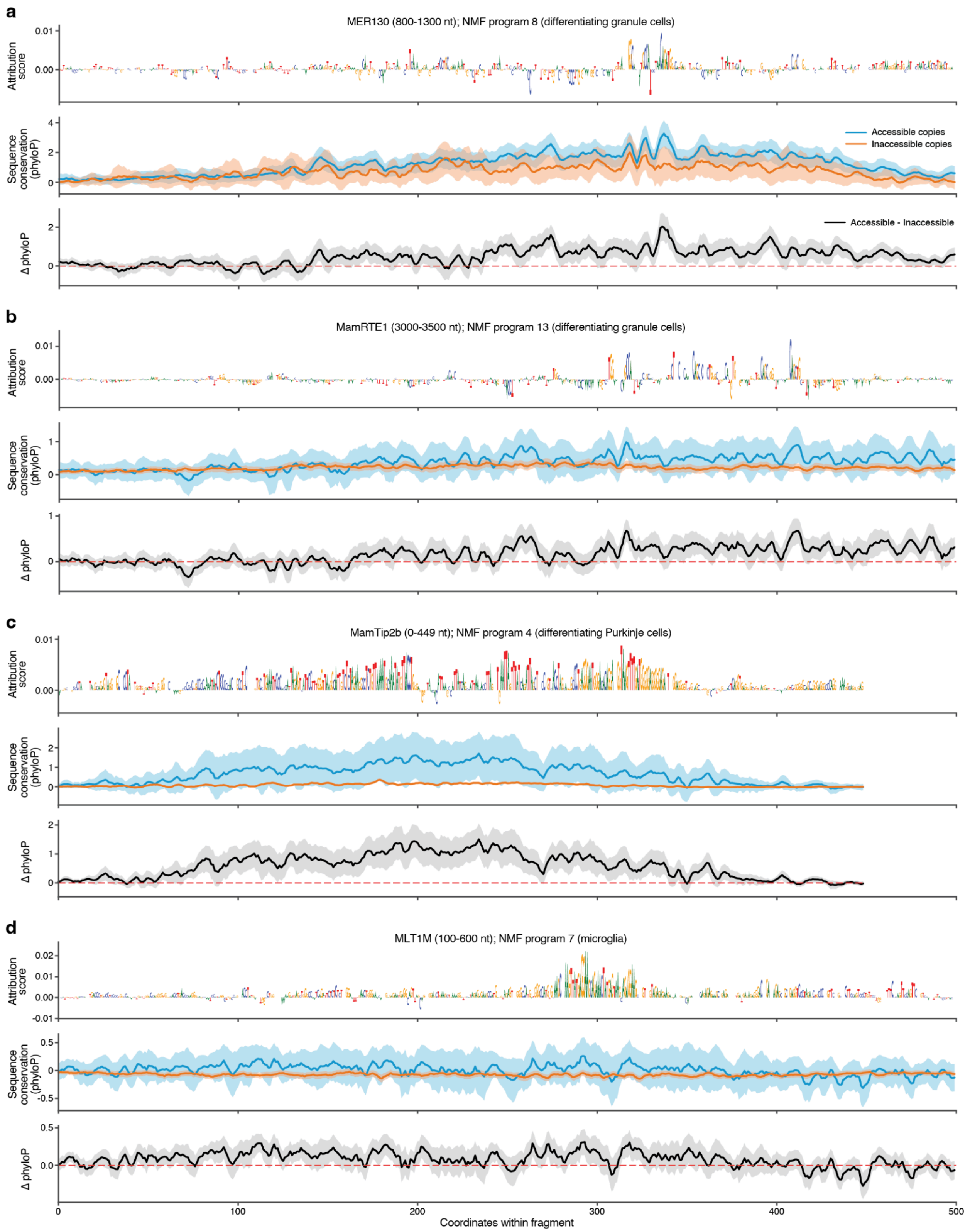

Supplementary Fig. 14 | Cross-mammalian conservation of sequences at ancestral TF binding motif instances in older screened TE subfamilies. a–d, *DeepExplainer attribution profile of the consensus sequence of the TE subfamily (top). Attribution profiles of accessible and inaccessible copies (middle) and the difference*

*between them (bottom), aligned to the consensus sequence, with the 95% confidence interval shown. Plots are shown for MER130 (**a**), MamRTE1(**b**), MamTip2b (**c**), and MLT1M (**d**).*

6) In Figure 5, the authors conclude that HERVL accessibility depends not only on motif preservation but also on the local chromatin context, such as the density of nearby cCREs. While this is biologically plausible, it raises an important conceptual issue regarding the interpretation of DeepCeREvo prediction scores. If accessibility is largely determined by the surrounding regulatory landscape, then high DeepCeREvo scores for non-accessible HERVL copies might reflect an overestimation of intrinsic enhancer potential.
Clarifying whether DeepCeREvo captures purely sequence-intrinsic potential or integrates features that indirectly correlate with genomic context would help readers interpret the discrepancy between predicted and observed accessibility.

DeepCeREvo takes solely 500-bp DNA sequences as input and is therefore unaware of anything outside this genomic window. It is expected that the local regulatory potential evaluated by DeepCeREvo does not fully explain accessibility profiles across the genome, given the influence of long-range interactions driven by dynamic genome organization. This limitation is also recognized in one of the state-of-the-art sequence-based models for chromatin accessibility, ChromBPNet, which uses a 2,114-bp input window (Pampari et al., bioRxiv, 2025 [DOI: 10.1101/2024.12.25.630221]]). Through newly implemented multivariate logistic regression analyses, we show that surrounding accessible cCREs can explain additional variation in accessibility beyond local (500-bp) DNA sequence features assessed by DeepCeREvo (Fig. 5m). Importantly, as demonstrated across multiple TE subfamilies, local sequence features remain by far the strongest predictor of TE accessibility (Fig. 5m; Supplementary Fig. 12). Although models with sm

all input windows are effective at detecting local sequence features contributing to chromatin accessibility, we acknowledge that larger input windows capable of modeling long-range chromatin interactions could improve genome-wide accessibility prediction, but this is still an area under active development. To clarify these points, we modified a sentence in the Results and added a limitation in the Discussion as follows:

> **[Page 6, Line 197]** *Specifically, we applied DeepCeREvo, a deep-learning model trained on human and mouse cCRE sequences that predicts program-specific cCREs based solely on 500 bp DNA sequences[39] (Fig. 2a).*
>
> **[Page 15, Line 544]** *An additional limitation is the relatively small input window (500 bp) of our sequence-based model, DeepCeREvo. This input size is sufficient to capture local sequence features such as TF binding motif instances, but a longer input window would allow for incorporating longer stretches of ancestral sequence, making it possible to evaluate the regulatory potential of long TE classes (e.g., LINE, ERV-int) as a whole, as well as the surrounding genetic context of extant TE copies, which could shed light on the effect of surrounding regulatory elements more clearly. Applying a sequence-based model with a larger input window, such as Borzoi[97] or AlphaGenome[98] (524 kb and 1 Mb input windows, respectively), to TE studies represents a promising direction for future research.*

7) Figure 6 suggests that species-specific accessible HERVL elements contribute to divergence in gene expression. However, given that accessibility appears to depend on insertion into pre-existing active chromatin regions enriched for non-TE cCREs (Fig. 5), it remains possible that these genomic neighborhoods were already primed for high activity before HERVL integration. In that case, HERVL insertions may have amplified rather than initiated species-specific expression programs. Clarifying whether HERVL served as a causal driver of regulatory divergence or acted as an enhancer of pre-existing regulatory trajectories would strengthen the interpretation.

We believe our data support the interpretation that HERVL insertions amplified pre-existing regulatory programs rather than initiating entirely new ones. We offer the following evidence:

1. Based on deep-learning model predictions applied to orthologous sequences, cCREs in the neighborhoods of accessible HERVL copies are inferred to be evolutionarily older than those surrounding inaccessible copies. This suggests that regulatory activity predated HERVL insertion in regions where these elements became accessible, consistent with integration into pre-existing active chromatin landscapes.
2. While genes near accessible human-specific HERVL elements show elevated expression in human compared to other species, they remain expressed, albeit at lower levels, in species lacking the HERVL insertion. This indicates that HERVL does not drive binary on/off expression switches but rather augments the expression of genes that were already part of active regulatory programs.

Together, these observations suggest that HERVL insertions became accessible in genomic contexts already primed for regulatory activity, where they contributed to species-specific regulatory divergence by augmenting existing gene expression programs. To clarify this interpretation, we have modified the Results section as follows:

> **[Page 11, Line 389]** *To assess whether accessible copies activate neighboring chromatin upon insertion or reside in pre-existing permissive environments, we examined the evolutionary history of surrounding cCREs using DeepCeREvo prediction scores across 228 mammalian species. cCREs near accessible HERVL copies have significantly older evolutionary origins than those near inaccessible copies (Fig. 5l), suggesting that accessible copies tend to reside in chromatin environments that were already active prior to HERVL insertion.*

> **[Page 13, Line 478]** *Despite this elevated expression in species with accessible copies, these genes still show detectable expression in species where the orthologous TE copy is inaccessible, consistent with our earlier finding that accessible copies tend to reside in pre-existing active chromatin environments. This suggests that TE insertions augment rather than establish regulatory activity.*

MINOR COMMENTS

- In Fig.1, the definition of TE-derived cCREs as requiring ≥50% (≥250 bp) overlap with a TE annotation seems quite conservative. Because many TE co-option events involve only partial fragments or individual motif remnants, this threshold might underestimate the true extent of TE-derived regulatory sequences. Please clarify the rationale for this choice, or comment on whether more relaxed thresholds (e.g., ≥50–100 bp) yield consistent trends.

We acknowledge that our ≥50% (≥250 bp) overlap criterion is stringent and may exclude TE co-option events involving shorter fragments or individual motif remnants. This conservative threshold maintains consistency with our deep learning model analysis, which evaluates regulatory potential of TE ancestral sequences at 500-bp resolution (the model's input window). For that analysis, it is critical that TEs contribute substantially to the regulatory element to minimize confounding effects from flanking non-TE sequences. By applying a conservative threshold in the general pattern analysis shown in the manuscript Fig. 1, we ensure that the TE-overlapping cCREs we characterized are conceptually aligned with those examined mechanistically in subsequent analyses. This criterion is also consistent with previous studies examining TE-derived regulatory elements (Judd et al., *Genome Biol.*, 2021 [PMID: 34187518]; Du et al., *Nat. Commun.*, 2024 [PMID: 39217141]). We clarified this rationale in the Methods section of the revised manuscript as follows:

> **[Page 16, Line 595]** *We intersected these curated transposable element annotations with CRE annotations using bedtools (v2.30.0)[101] and only retained overlaps longer than 250 bp (i.e., half of the cCRE length).* <u>*This conservative ≥50% overlap threshold ensures that TE sequences constitute the majority of each TE-derived cCRE and is consistent with previous studies examining TE-derived regulatory elements*[16,20].</u>

- In Figure 2b, the use of a whole-body chromatin accessibility atlas as background is conceptually sound. However, because TE enrichment can be influenced by genomic context and sequence composition, the analysis would benefit from additional controls matched for GC content, CpG density, peak length, mappability, conservation, and regulatory context (e.g., promoter versus distal). Stratified or nearest-neighbor matching, or complementary permutation-based background models (e.g., circular chromosome randomization), would provide a more rigorous assessment that the observed positional enrichments reflect biologically specific co-option rather than underlying genomic biases.

We appreciate the reviewer's suggestion to control for sequence features of the background cCRE set. We agree that such controls are important for genome-wide cCRE–TE enrichment analyses, and we have implemented them for our global overlap analyses (Fig. 1a–f; see also our response to Reviewer #2, Comment 1-1, page 18 of this response letter), where we used shuffled genomic background regions and logistic regression controlling for GC content and cCRE type.

For the analysis in Fig. 2b, however, feature-matched background construction is not straightforward. The unit of analysis is a 500-bp sliding window tiled across each TE consensus

sequence, and for each window we compare the overlap rate with cell-type-specific cCREs against that with whole-body atlas cCREs. Because the windows are defined by TE sequence coordinates rather than by cCRE properties, constructing a background cCRE set matched for features such as peak length, GC content, or regulatory context would require constraining the background in ways that may not always have appropriate matched counterparts for every TE window across the 123 screened subfamilies.

Importantly, our analytical framework already addresses the reviewer's core concern—whether the observed enrichments could be driven by sequence composition biases—through a complementary approach. The DeepCeREvo analysis evaluates the cis-regulatory potential of each 500-bp TE window by comparing model predictions against dinucleotide-shuffled control sequences, which explicitly controls for nucleotide and dinucleotide composition (Fig. 2a). The convergence between this sequence-composition-controlled approach and the overlap-based enrichment analysis (Fig. 2b) provides strong evidence that the identified positional enrichments reflect genuine regulatory potential encoded in the TE sequences rather than systematic biases in the underlying sequence composition of the TE windows.

Furthermore, we chose the whole-body chromatin accessibility atlas as the background because our goal is to identify cell-type-specific TE co-option. This atlas captures actual accessibility across diverse fetal and adult tissues, providing the biologically appropriate contrast for identifying cerebellar-specific regulatory activity. Permutation-based approaches such as circular chromosome randomization would test whether TEs are enriched in accessible chromatin in general but would not capture the tissue-specific co-option that is central to this analysis.

Finally, the biological validity of the identified enrichments is supported by orthogonal evidence: the predicted motif instances correspond to known transcription factors expressed in the relevant cell types (Fig. 3; Supplementary Figs. 6,7), ancestral motif positions are preferentially preserved in accessible copies (Fig. 5e,f; Supplementary Figs. 13,14), and functional reporter assays confirm enhancer activity of the ancestral sequences of HERVL (Fig. 4d).

- (This point is related to Major comment 5.) The authors conclude that HERVL-specific substitutions generated TF-binding motifs underlying enhancer activity. This is a compelling model; however, the evidence would be considerably strengthened by showing conservation of these motif sites across genomic HERVL-derived cCRE copies and among different primate species. A multiple sequence alignment or conservation plot demonstrating that the key motif nucleotides are consistently preserved among active copies would provide direct support for the functional relevance of these substitutions.

In the original manuscript, we built a multiple sequence alignment of HERVL copies in the human genome with the ancestral sequence as a reference and showed that accessible HERVL copies preserve ancestral sequences at positions corresponding to transcription factor binding motifs more than inaccessible copies (Reviewer Figure 4). We chose this visualisation instead of directly plotting the MSA for two reasons: (1) the large number of HERVL copies (~500) makes a full multiple

sequence alignment visually unintuitive, and (2) even in inaccessible copies, these sequences are reasonably well preserved (~80%) due to the relatively short time since insertion and the small number of substitutions from the ancestral sequence, making it difficult to discern differences in nucleotide preservation between accessible and inaccessible copies from a raw MSA.

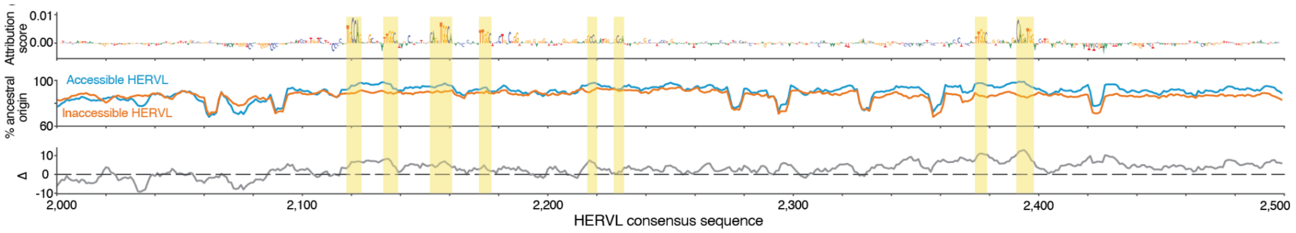

Reviewer Figure 4 (Fig. 5e of the original manuscript) | Sequence conservation analysis of HERVL copies showing DeepExplainer attribution profile of the HERVL ancestral (consensus) sequence (top), percentage of nucleotides identical to the ancestral sequence in accessible versus inaccessible HERVL copies (middle), and differential conservation pattern between accessible and inaccessible copies (bottom). TF binding sites in the consensus sequence are highlighted.

To provide formal statistical support for this preservation pattern, we have now added 95% confidence intervals to the proportions of nucleotide preservation (Fig. 5e,f) and performed formal statistical testing using linear regression while controlling for nucleotide identity. The results demonstrate that accessible HERVL copies preserve ancestral sequences at high-attribution positions significantly more than inaccessible copies (coefficient = 0.03, $P < 10^{-7}$), directly supporting the functional relevance of these motif sequences.

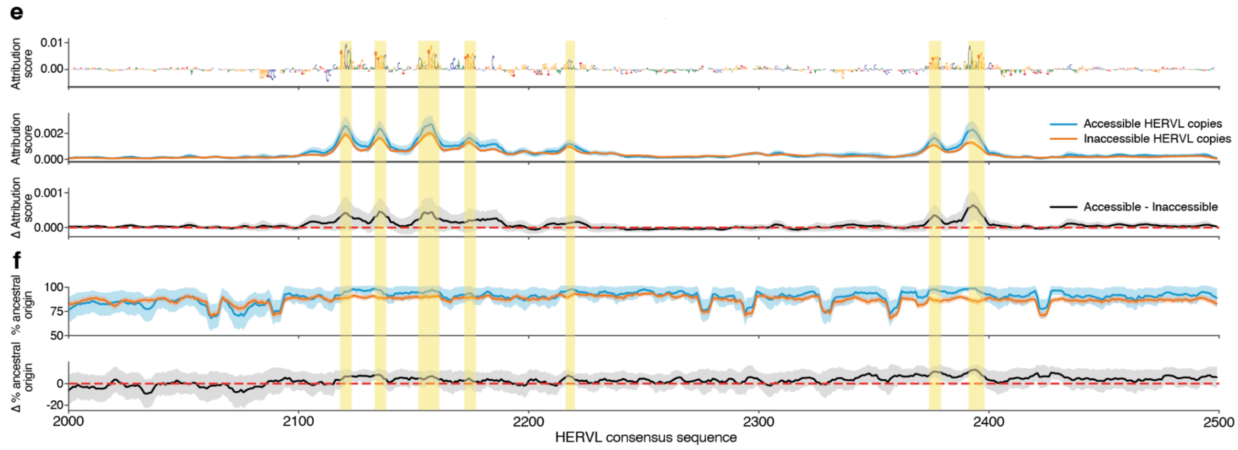

***Fig. 5e, f* | *e*, *DeepExplainer attribution profile of the HERVL consensus sequence (top); mean DeepExplainer attribution profiles of accessible and inaccessible HERVL copies (middle) and the difference between them (bottom), with the 95% confidence interval shown. TF binding motif instances in the consensus sequence are highlighted. **f**, Percentage of nucleotides identical to the consensus sequence in accessible versus inaccessible HERVL copies (top), and the difference between them (bottom), with the 95% confidence interval shown.*

Regarding cross-species conservation, we also analysed phastCons scores across 30 primate species. While we detected a statistically significant difference in conservation between accessible and

inaccessible HERVL copies, the effect size is modest and we consider this analysis inconclusive, owing to the recent origin of HERVL insertions and the lineage-specific nature of chromatin accessibility. Please see our detailed response to Major Comment #5 for the full analysis and interpretation (page 48 of this response letter).

- Recent work by Sekine et al. (Communications Biology, 2023) has also demonstrated that transposable elements shape cell-type-specific open chromatin landscapes in the mouse brain. Citing this study would help appropriately contextualize the novelty of the present findings within the broader literature on TE-mediated regulatory evolution.

Thank you for this valuable suggestion. We agree that Sekine et al. *Commun. Biol.*, 2023 [PMID: 37301950] is an important study worth citing in this context. We have now added this reference to the introduction and modified the text as follows:

> **[Page 2, Line 67]** *Finally, most previous studies, with some recent exceptions[28,29], relied on bulk tissue or isolated cell populations[14,16–20,30–32] or lacked insights into the mechanisms of cell-type-specific TE co-option[33–36], limiting our understanding of TE contributions to gene regulation, which fundamentally operates at the level of individual cell types within tissues.*

- Figure panel cross-references appear to be mismatched in several places. Please verify and correct the following:

• Lines 254–255: "Fig. 3a" ↔ "Fig. 3b" appear swapped

o l254: change Fig. 3a → Fig. 3b

o l255: change Fig. 3b → Fig. 3a

• Lines 298–301: References to Fig. 3e,f seem off

o l298: change Fig. 3e → Fig. 3g

o l301: change Fig. 3e,f → Fig. 3g,h

• Lines 320–324: Fig. 4a/b appear reversed

o l320: change Fig. 4a → Fig. 4b

o l324: change Fig. 4b → Fig. 4a

We thank the reviewer for carefully identifying these mismatched panel references. Following substantial revision of the manuscript, the figure panel numbering has changed considerably from the original version. However, we have thoroughly verified that all figure references in the revised text correctly correspond to their intended panels.

Reviewer: 4

I co-reviewed this manuscript with one of the reviewers who provided the listed reports. This is part of the Nature Communications initiative to facilitate training in peer review and to provide appropriate recognition for Early Career Researchers who co-review manuscripts.

We thank Reviewer #4 for their time and effort in co-reviewing our manuscript.

Response to the referees #2

We thank the referees for their thorough re-evaluation of our manuscript and for their positive and encouraging feedback. We have carefully addressed the remaining minor suggestions, as detailed below.

Reviewer: 1

The authors have addressed all our critique and the manuscript has been substantially revised. We are happy with the changes.

We are grateful for your kind and encouraging words. We greatly appreciate your recognition of our revisions and your support for publication. Your comments have been invaluable in helping us improve the manuscript.

Reviewer: 2

I thank the authors for a comprehensively satisfying set of revisions in answer to my originally raised questions.

We are grateful for your kind and encouraging words. We greatly appreciate your recognition of our revisions and your support for publication. Your comments have been invaluable in helping us improve the manuscript.

Reviewer: 3

The authors have responded carefully and convincingly to all of my comments. The revised manuscript is substantially improved by additional analyses that clarify the scope of the study, extend the findings beyond the original HERVL-focused case study, and strengthen the statistical support for the major conclusions. In particular, the analyses of additional TE subfamilies now better distinguish different modes of TE co-option, including ancestral regulatory potential and convergent acquisition of new motif instances. I also appreciate the more nuanced interpretation that TE-derived elements may amplify pre-existing regulatory programs rather than simply create new regulatory circuits.

Overall, I am satisfied with the revision and recommend publication. I have only a few minor suggestions.

We sincerely thank you for your thorough and thoughtful evaluation of our revised manuscript. We are pleased that the additional analyses addressing your comments have strengthened the study, and we greatly appreciate your recognition of the improvements made. We also thank you for your continued engagement with our work and for the few remaining minor suggestions, which we address below.

MINOR SUGGESTIONS

1) The LTR1A2 convergent evolution finding (Supp Fig. 16) is one of the most interesting additions—consider giving it slightly more prominence in the Discussion as it elegantly demonstrates that "TE co-option" is not a monolithic phenomenon.

This is a great suggestion. We have now revised the Discussion to give greater prominence to the LTR1A2 convergent evolution finding, which now reads as follows:

> **[Page 15, Line 518]** *Furthermore, we used DeepCeREvo to show that preservation of ancestral sequences and proximity to pre-existing active chromatin contribute to co-option at the individual copy level, in line with observations of RSINE1 co-opted as a circadian enhancer in mouse*[20]*. Notably, this ancestral sequence preservation operates through distinct modes: HERVL harbors "ready-to-use" pre-adaptive sequences, whereas LTR1A2 possesses proto-regulatory potential that was subsequently augmented through convergent acquisition of new TF binding motif instances across independent copies. These lines of evidence further illustrate the multifaceted nature of TE co-option as regulatory elements, while also demonstrating the utility of sequence-based models in understanding this process at cell-type resolution across TE subfamilies.*

2) I noticed that the colors in the TE family legend in Fig. 1g may not fully match the colors shown in the corresponding plot, although this could be a PDF conversion issue. I suggest that the authors check the final figure carefully during production.

We thank the reviewer for catching this issue. We have confirmed that this was an error in the figure and have now updated Fig. 1g so that the colors in the legend fully correspond to those in the plot.

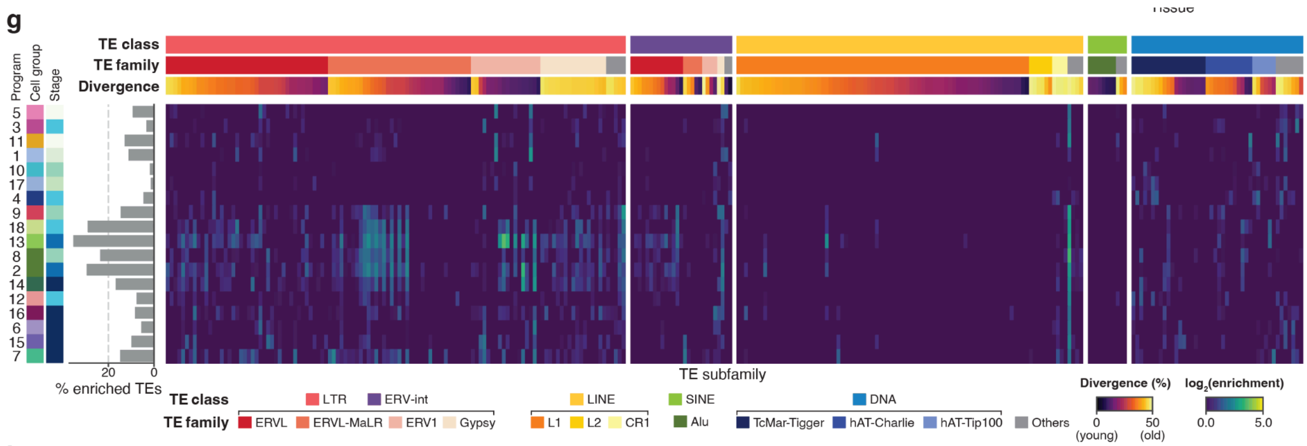

Fig. 1g | Subfamily-specific TE enrichment within program-specific cCREs. (Left) bar plot indicates the proportion of each enriched TE subfamily contributing to TE-derived cCREs. Note that highlights for LTR1A2 and MamRTE1 are only added in this response.

Reviewer: 4

I co-reviewed this manuscript with one of the reviewers who provided the listed reports. This is part of the Nature Communications initiative to facilitate training in peer review and to provide appropriate recognition for Early Career Researchers who co-review manuscripts.

We thank the reviewer for their contribution to the peer review of this manuscript
